# Supplementary material for: Bilateral Remote Ischaemic Conditioning in Children (BRICC) trial: protocol for a two-centre, double-blind, randomised controlled trial in young children undergoing cardiac surgery
Source: BMJ Open. 2020 Oct 7;10(10):e042176. doi: 10.1136/bmjopen-2020-042176 (PMC7542918; doi:10.1136/bmjopen-2020-042176)
Supplement: Supplementary data [file bmjopen-2020-042176supp001.pdf]

# PARENT/GUARDIAN INFORMATION SHEET

**The Bilateral Remote Ischaemic Conditioning in Children trial**

Chief Investigator: Mr Nigel Drury, Consultant in Paediatric Cardiac Surgery, BCH

*An invitation to participate in research*: The Heart Surgery team at Birmingham Children's Hospital would like to invite your child to take part in a research study. Before you decide, you need to understand why the research is being done and what it would involve for you and your child. Please read the following information carefully and take time to decide whether or not you would like your child to take part. If there is anything that is not clear or you would like more information, please ask.

## Why is my child being invited to take part?

Your child has been referred to the Heart Surgery team for an operation to repair one of two common congenital heart conditions: Tetralogy of Fallot (TOF) or a Ventricular Septal Defect (VSD). We are performing a clinical trial in young children with these conditions and have approached you to see if you would like your child to take part.

## What is the aim of the study?

Children like yours are born with congenital heart disease and often need operations to correct the abnormality that they were born with to improve their survival. The surgery is complex and usually involves a period of support on a heart-lung machine (cardiopulmonary bypass) whilst the defect is repaired. The surgery puts a strain on your child's heart and may potentially cause damage (called ischaemia-reperfusion injury) when the blood supply to the heart is interrupted and restored. In this study, we wish to determine if a simple technique of inflating and deflating a blood pressure cuff immediately before the operation can reduce damage to the heart during surgery for two common conditions, to improve the outcomes of children's heart surgery.

## What procedure is being tested in this study?

We are testing whether a simple procedure, the inflation and deflation of blood pressure cuffs on both legs immediately before heart surgery, can help to protect the heart from injury during surgery for different types of congenital heart disease. No drugs are being tested in this study, only the effects of cuff inflation.

## How might inflating blood pressure cuffs on the legs help to protect the heart?

It has been shown that reducing the blood flow to the arms or legs for a short period can protect the heart, lung and kidneys from injury in adults and children undergoing different types of surgery. The temporary stoppage of blood to the limb activates a reflex known as remote ischaemic pre-conditioning (RIC). Researchers in several countries, including Canada & Australia, have shown that this may reduce the extent of heart damage in young children after surgery for congenital heart disease. We are performing this study to see whether there is a difference between children who have low oxygen levels in the blood (cyanosis) and those who do not.

## Will my child undergo the blood pressure cuff treatment?

This study is a double-blind, randomised controlled trial. This means that if you agree for your child to take part, they will be allocated by a computer with a 50% chance of receiving RIC with the blood pressure cuffs and a 50% chance of not receiving RIC. All other aspects of the anaesthetic, surgery and post-operative care will be the same and *neither* you *nor* the surgical team will know whether your child has received RIC. At the end of the study, the code will be revealed to see which children were in which group. This is a standard technique for preventing those doctors and nurses involved in conducting a clinical trial from potentially influencing the results.

## What will happen if I agree for my child to take part?

In addition to the standard operation and post-operative care, if you agree for your child to take part in the study, the following will occur:

- Your child's Paediatric Cardiologist and with your permission, your child's GP will be informed of their participation.
- Your child will be allocated to either the RIC group or the control group by chance.
- Once they are asleep under anaesthesia, if they are in the RIC group, a blood pressure cuff will be placed around each of their upper thighs and inflated to a

level approximately 50mmHg higher than their own blood pressure – this will not cause any pain. The cuffs will remain inflated for 5 minutes then deflated for 5 minutes and will be repeated two more times. If they are in the control group, the blood pressure cuffs will not be placed on their legs.

- Prior to surgery, all children have small plastic lines (tubes) inserted into their blood vessels to make measurements and take blood samples. Children in the study will have additional blood samples taken from these lines (no extra needles) over the first 24 hours after surgery to detect any injury to the heart.
- During surgery, in order to repair the defect, the heart is opened through a cut in the side of the right atrium. In children in the study, two small biopsies of the atrium will be taken from the edge of the cut by the operating surgeon. Once the heart defect is repaired, the cut will be closed in the normal way; taking the biopsies does not increase the risk of the operation in any way but will allow us to understand how RIC may improve protection of the heart.
- In children with TOF, bands of muscle inside the heart which blocks the flow of blood to the lungs are routinely removed during surgery; if your child is in the study, these bands of muscle will be kept for analysis rather than be thrown away.
- After surgery, your child will be discharged home and kept under regular follow-up in the clinic; you will not need to attend any additional clinic visits for the study.

## What are the benefits?

There may not be any benefits for your child. Whilst some previous studies have shown that RIC helps to protect children's hearts from injury during surgery, we do not know whether it is beneficial to all children with all types of congenital heart disease - that is why we are conducting this study. We do not know whether being in the study will make your child's surgery safer but we are conducting it in order to understand how to improve the outcomes of children's heart surgery in the future.

## Are there any risks?

Previous studies have shown that RIC is safe. There have been no complications reported related to the use of a blood pressure cuff for RIC in either children or adults undergoing *any* type of surgery. The operation itself carries a risk for your child, as will have been discussed with you by your Surgeon and Cardiologist, but being involved in this study does not increase that risk in any way.

## How many children will be taking part in this study?

We aim to recruit up to 120 children undergoing surgery for congenital heart disease in Birmingham and Leeds to take part in this study over a 4 year period.

## Does my child have to take part?

Taking part in the study is entirely voluntary – you decide. This parent information sheet gives you information about the study and we can answer any questions that you may have after reading it. Before your child's surgery, one of the research team will ask you whether you wish your child to participate in this study and if so, to sign a consent form. Your child will only be included in the study if you give your express permission. Indeed, you are free to withdraw your child at any time, without giving a reason – their surgery will proceed as planned, without any additional measurements and tests, and it will not affect the standard of care that your child receives.

## What if something goes wrong?

The standard care of children undergoing heart surgery involves intensive monitoring and we do not expect the study itself to cause any problems. Complications of surgery can occur and these will be dealt with in the normal manner, regardless of the research study. Your child's safety during and after surgery is paramount. In the unlikely event that any harm should occur as a result of taking part in this study, we want you to be informed of your rights. There are no special compensation arrangements but you may have the right to claim damages in a court of law; this would require you to prove fault on the part of the NHS Trust, University or any manufacturer involved. The standard NHS complaints mechanisms are available to you; further information can be obtained from the Patient Advice & Liaison Service (PALS) at Birmingham Children's Hospital on 0121 333 8611.

## What happens to my child's information and samples?

All information collected on children who participate in this study will be securely stored on Hospital and University computers. Paper copies of the data will be stored in a locked office at the Hospital. The information from the study will be analysed, presented at scientific meetings and published in medical journals to inform other doctors and health professionals of the research findings. All data will be coded and kept confidential, ensuring that your child's identity will not be revealed at any time.

All necessary measures will be taken to keep your child's data safe and to comply with the Data Protection Act. Following completion of the study, the data will be kept for 25 years then destroyed in accordance with national guidance. All of the blood and tissue samples collected during the study will stored in secure laboratories at the hospital, collaborating hospitals & University in accordance with Human Tissue Act. Once analysed, any remaining samples may be kept and used in future research studies which conform to all relevant legal, governance and ethical requirements.

### What happens at the end of the study?

At the end of the study, your child's treatment and follow-up continues as would that of a child who had not been involved in the study.

### Will I ever know if the trial worked and which treatment my child received?

Yes – but not until the whole study has finished and we have analysed the results. We will work with *Young at Heart*, a charity that offers help and support to families of babies and children diagnosed with heart defects, to produce a newsletter with the findings of the study to send to the parents of all children involved. At this stage, we will be able to tell you which group your child was in.

### Who is organising and funding this research?

This study has been organised & developed by the teams at Birmingham Children's Hospital, Leeds Teaching Hospitals NHS Trust and University of Birmingham. It is funded by the **British Heart Foundation** & sponsored by University of Birmingham.

### Who has reviewed this research study and leaflet?

The study has been reviewed by the British Heart Foundation and the Research & Development teams at Birmingham Children's Hospital, Leeds Teaching Hospitals NHS Trust and the University of Birmingham. It has been given a favourable opinion for conduct in the NHS by the West Midlands Solihull Research Ethics Committee. This Parent Information Sheet has been reviewed and revised by the parents of children who have had heart surgery, through the children's charity *Young at Heart*.

**Questions?** Contact Mr. Nigel Drury, Consultant in Paediatric Cardiac Surgery by email: xxxxx.xxxxx@nhs.net or via the BCH switchboard on xxxx xxx xxxx.

## Flow chart for the Bilateral Remote Ischaemic Conditioning in Children trial

You will have been given this information sheet by your Consultant or a member of the research team in the clinic or been sent it in the post.

↓

Please read it carefully and consider whether you would like your child to take part. If you have any questions, please feel free to ask.

↓

We will contact you either at the pre-operative assessment clinic or when your child is admitted for their operation.

↓

If you are happy for your child to take part, you will be asked to sign a Consent form by a member of the research team.

↓

On the day of surgery, you may go along to theatre with your child as usual. After they have gone to sleep, the computer will allocate them to a group and they will receive *either* RIC *or* no RIC just before their operation. During surgery, the blood & tissue samples will be removed for analysis.

↓

After the operation, they will be transferred to the Paediatric Intensive Care Unit (PICU) as usual and you will be able to see them there.

↓

Whilst they are recovering on PICU, several additional blood tests will be performed using the lines already in place – no new needles required.

↓

When they are well enough, your child will be transferred to the ward and then discharged from hospital once they are ready to go home.

↓

After discharge, they will be seen regularly in the outpatient clinic but there will be no additional follow-up appointment related to the study.

↓

Once the trial has completed, we will send you a newsletter with the results.

**Thank you for reading this information & considering your child's participation**
