# Supplementary material for: Bilateral Remote Ischaemic Conditioning in Children (BRICC) trial: protocol for a two-centre, double-blind, randomised controlled trial in young children undergoing cardiac surgery
Source: BMJ Open. 2020 Oct 7;10(10):e042176. doi: 10.1136/bmjopen-2020-042176 (PMC7542918; doi:10.1136/bmjopen-2020-042176)
Supplement: Supplementary data [file bmjopen-2020-042176supp003.pdf]

1 BRICC trial – ISRCTN12923441 IRAS: 200876 Version 1.9a: 22/11/2019

British Heart Foundation | UNIVERSITY OF BIRMINGHAM | BCTU Birmingham Clinical Trials Unit | NHS Birmingham Women's and Children's NHS Foundation Trust

PARENT/GUARDIAN CONSENT FORM

The Bilateral Remote Ischaemic Conditioning in Children trial

Chief Investigator: Mr Nigel Drury, Consultant in Paediatric Cardiac Surgery, BCH

Please initial boxes

I confirm that I have read and understand the parent/guardian information sheet (version 1.9a, dated 22/11/2019) for the above study. I have had the opportunity to consider the study information, ask questions and have had these answered satisfactorily. ☐

I understand that my child's participation is voluntary and that I am free to withdraw at any time, without giving a reason and without my child's care or legal rights being affected. ☐

I understand that relevant sections of any of my child's medical notes and data collected during the study may be looked at by responsible individuals from the NHS Trusts, the University of Birmingham or the regulatory authorities, where it is relevant to my taking part in this research. I give permission for these individuals to have access to my child's records. ☐

I consent to the storage, including electronic, of personal information for the purposes of this study. I understand that any information that could identify me or my child will be kept strictly confidential and that no personal information will be included in the study report or other publication. ☐

I understand that blood and tissue samples will be kept for the purposes of research and I give permission for these samples to be taken and stored. ☐

I understand that any remaining samples may be stored beyond the end of this trial and used in future research which conforms to all relevant legal, governance and ethical requirements. ☐

I agree to my child's GP and/or other doctors involved in their care, being informed of my child's participation in the study. ☐

Name of Child: Date of birth:

Name of Parent/Guardian	Signature of Parent/Guardian	Date signed
Name of Investigator	Signature of Investigator	Date signed

Once completed: 1 for parent(s), 1 for medical notes and 1 (original) for trial site file
