# Supplementary material for: Bilateral Remote Ischaemic Conditioning in Children (BRICC) trial: protocol for a two-centre, double-blind, randomised controlled trial in young children undergoing cardiac surgery
Source: BMJ Open. 2020 Oct 7;10(10):e042176. doi: 10.1136/bmjopen-2020-042176 (PMC7542918; doi:10.1136/bmjopen-2020-042176)
Supplement: Supplementary data [file bmjopen-2020-042176supp005.pdf]

Data Monitoring Committee (DMC) Charter

1. INTRODUCTION

Title: The Bilateral Remote Ischaemic Conditioning in Children (BRICC) trial: a two-centre, double-blind, randomised controlled trial of remote ischaemic preconditioning in normoxic and hypoxic children undergoing cardiac surgery – ISRCTN12923441.

Chief Investigator: Mr Nigel Drury, Consultant in Paediatric Cardiac Surgery, BCH

TMC Chair: Mr Timothy Jones, Consultant Paediatric Cardiac Surgeon, BCH

IRAS: 200876, protocol: v1.6, 05/02/18, approved by REC: 28/02/18, HRA: 28/02/18.

Sponsor: University of Birmingham (RG_14-025), in partnership with Birmingham Women's and Children's NHS Foundation Trust (1845) and Leeds Teaching Hospitals NHS Trust (PA17/67348).

Funder: British Heart Foundation (FS/15/49/31612)

Objectives: The BRICC trial is a two-centre, double-blind, randomised controlled trial to assess whether adequately delivered remote ischaemic preconditioning is cardioprotective in young children undergoing surgical repair of two common congenital heart defects, through clinical and biochemical endpoints. It will also evaluate the effects of preconditioning in the presence or absence of chronic preoperative hypoxia by evaluating the myocardial metabolic phenotype.

Scope: The purpose of this document is to describe the roles and responsibilities of the independent DMC for the BRICC trial, including the terms of reference, the frequency and format of meetings, methods of providing information to and from the DMC, statistical issues and relationship with the Trial Management Committee.

2. ROLES & RESPONSIBILITIES

Aims: To safeguard the interests of trial participants & their families, assess the safety and efficacy of the interventions during the trial and monitor the overall conduct of the clinical trial.

Terms of reference: The DMC should receive and review the progress and accruing data from the trial and make recommendations on the conduct of the trial to the Trial Management Committee. Meetings will be scheduled approximately every 8 months and key outputs will be made available for review; changes to this schedule may be made by the DMC as required. Review(s) will include updated figures on recruitment, data quality, efficacy outcomes and safety data. Specifically, the DMC will:

- Assess data quality, including completeness
- Monitor recruitment and losses to follow-up
- Monitor compliance with the protocol by participants and investigators
- Monitor evidence for treatment differences in the main efficacy outcomes
- Monitor evidence of treatment harm (limb complications, SAEs)
- Decide whether to recommend that the trial continues or whether recruitment should be terminated for some or all of the treatment groups
- Suggest additional data analyses
- Advise on protocol modifications suggested by the Investigators
- Assess the impact and relevance of external evidence
- Monitor compliance with previous DMC recommendations

Furthermore, members of the DMC will not disclose interim results or use them for financial gain, nor share blinded data with anyone outside of the DMC, including the Chief Investigator. All DMC and TMC members will sign a Conflict of Interest form.

3. BEFORE OR EARLY IN THE TRIAL

Members of the DMC were invited to participate in the committee in the spring of 2016 and therefore were not involved in development of the trial protocol. The DMC were asked to meet before or early in the trial (within 3 months of commencing recruitment) to review this Charter, revise as required and approve the working version; this initial meeting took place on 18th November 2016.

4. COMPOSITION

The members of the independent DMC for the BRICC trial are:

- Prof Gavin Murphy, BHF Chair of Cardiac Surgery, University of Leicester (Chair)
- Dr Katherine Brown, Consultant in Cardiac Intensive Care, GOSH, London
- Dr Peter Nightingale, Statistician, Queen Elizabeth Hospital Birmingham

At their initial meeting, the members of the DMC nominated Prof Murphy as Chair with responsibility for facilitating discussion and communicating review outcomes.

5. RELATIONSHIPS

In addition to the independent DMC, the conduct of the trial is overseen by members of the Trial Management Committee, who have developed and approved the trial protocol and have executive responsibility for the conduct of the trial; there is no Trial Steering Committee. The DMC has an advisory role and will make recommendations regarding efficacy and the safety of participants to the Trial Management Committee.

6. ORGANISATION

The DMC discussed the first version of this Charter and made recommendations based on best practice which have been implemented. During the trial, they will meet every 8 months to review efficacy and safety data. Analysis of hs-troponin-T for the primary outcome will be performed prior to each meeting and the unblinded data made available. Meetings may be held via teleconference, without the need to meet face-to-face unless deemed necessary, and will employ the following format:

1. *Open session*: Introduction, presentation of progress report
2. *Closed session*: Discussion of closed parts of the report
3. *Open session*: Discussion of any matters arising from previous sessions
4. *Closed session*: Further discussion, if required.

Closed sessions will be attended only by members of the DMC and others whom are specifically invited as discussion will include unblinded efficacy and safety data by treatment group. In open sessions, they will be joined by the Chief Investigator, Trial Statistician and other interested parties which may include Research Nurse(s) and representatives of the R&D Office, sponsor, funder or regulator, as required.

7. DOCUMENTATION

The Chief Investigator and Trial Statistician will produce a progress report for each DMC review, which should be received by members at least 2 weeks beforehand, documenting the key outputs of the trial including graphs showing expected and observed recruitment, CONSORT diagrams, descriptions of the trial cohort, protocol compliance, procedural and outcome measures, and adverse events reported by group. Specifically it will include:

- *Primary efficacy outcome*: area under the time-concentration curve for hs-troponin-T release in the first 24 hours after aortic cross-clamp release, calculated by the trapezoid rule from samples taken at baseline, 3, 6, 12 and 24 hours.
- *Primary safety outcome*: frequency of expected Serious Adverse Events (SAEs) reported to the Sponsor: death; requirement for extracorporeal life support (ECLS); evidence of a major neurological event; or the need for further surgery in the early post-operative period, such as for bleeding or a residual VSD.
- *Secondary efficacy outcomes*: peak hs-troponin-T in the first 12 hours; vasoactive inotrope score in the first 12 hours; arterial lactate and central venous oxygen saturations in first 12 hours; and lengths of stay in the paediatric intensive care unit and the hospital. Cardiac index in the first 12 hours is an exploratory outcome.

DMC members should store their papers and reports safely after each meeting so that they can cross-check with later reports. After the trial is finally reported, all interim reports should be destroyed.

8. DECISION MAKING

The DMC should consider the following possible recommendations following interim analysis of the conduct of the trial and the safety and efficacy data:

1. No action needed, trial continues as planned.
2. Early stopping due to clear treatment benefit or harm, futility or external evidence.
3. Stopping recruitment within a subgroup.
4. Extending recruitment (based on actual control arm response rates being different to predicted rather than on emerging differences) or extending follow-up.
5. Sanctioning and/or proposing protocol changes.

The DMC agreed that no firm statistical criteria (often called *stopping rules*) would be defined, rather DMC recommendations would be based on the ability of the trial to test its primary hypothesis and the overall interpretation of the safety data and secondary outcomes that include important safety endpoints. The DMC will consider the balance of primary risks and benefits, the internal consistency of results, the consistence with and nature of external evidence, and the likelihood that results would affect clinical practice. Decisions should be achieved by consensus and be unanimous when possible, using both informal and formal decision-making strategies, as required.

If the DMC recommends stopping recruitment, an urgent face-to-face meeting should be arranged between the DMC Chair, Chief Investigator, key members of the Trial Management Committee and the Sponsor to reach a decision on continuing the trial.

9. REPORTING

The DMC will report its recommendations in writing to the Trial Management Committee and Sponsor. A formal record should also be made of both closed and open sessions, documenting the major points of discussion, any decisions and actions and their reasons, and any additional information needed for future meetings; however, names do not need to be attributed to all comments.

10. AFTER THE TRIAL

Responsibility for reporting the findings of the trial lies with the Chief Investigator and Trial Management Committee, although the DMC should encourage timely reporting. The DMC Chair should approve the accuracy of trial manuscripts submitted for publication in which all members of the DMC will be acknowledged.

REFERENCES

- DAMOCLES Study Group. A proposed charter for clinical trial data monitoring committees: helping them to do their job well. *Lancet* 2005; 365: 711-22.
- National Research Ethics Service. Data Monitoring Committees in Clinical Trials. Guidance for Research Ethics Committees. NPSA, May 2010
