# Supplementary material for: Bilateral Remote Ischaemic Conditioning in Children (BRICC) trial: protocol for a two-centre, double-blind, randomised controlled trial in young children undergoing cardiac surgery
Source: BMJ Open. 2020 Oct 7;10(10):e042176. doi: 10.1136/bmjopen-2020-042176 (PMC7542918; doi:10.1136/bmjopen-2020-042176)
Supplement: Author's manuscript [file bmjopen-2020-042176.draft_revisions.pdf]

BMJ Open

BMJ Open is committed to open peer review. As part of this commitment we make the peer review history of every article we publish publicly available.

When an article is published we post the peer reviewers' comments and the authors' responses online. We also post the versions of the paper that were used during peer review. These are the versions that the peer review comments apply to.

The versions of the paper that follow are the versions that were submitted during the peer review process. They are not the versions of record or the final published versions. They should not be cited or distributed as the published version of this manuscript.

BMJ Open is an open access journal and the full, final, typeset and author-corrected version of record of the manuscript is available on our site with no access controls, subscription charges or pay-per-view fees (http://bmjopen.bmj.com).

If you have any questions on BMJ Open's open peer review process please email info.bmjopen@bmj.com

BMJ Open

The Bilateral Remote Ischaemic Conditioning in Children (BRICC) trial: protocol for a two-centre, double-blind, randomised controlled trial in young children undergoing cardiac surgery

Journal:	*BMJ Open*
Manuscript ID	bmjopen-2020-042176
Article Type:	Protocol
Date Submitted by the Author:	27-Jun-2020
Complete List of Authors:	Drury, Nigel ; Birmingham Children's Hospital, Paediatric Cardiac Surgery; University of Birmingham, Institute of Cardiovascular Sciences<br>Bi, Rehana; Birmingham Children's Hospital, Paediatric Cardiac Surgery; Birmingham Children's Hospital, Paediatric Intensive Care<br>Woolley, Rebecca; University of Birmingham, Birmingham Clinical Trials Unit; University of Birmingham, Institute of Applied Health Research<br>Stickley, John; Birmingham Children's Hospital, Paediatric Cardiac Surgery<br>Morris, Kevin; Birmingham Children's Hospital, Paediatric Intensive Care; University of Birmingham, Institute of Applied Health Research<br>Montgomerie, James; Birmingham Children's Hospital, Paediatric Cardiac Anaesthesia<br>van Doorn, Carin; Leeds Teaching Hospitals NHS Trust, Paediatric Cardiac Surgery<br>Dunn, Warwick; University of Birmingham, School of Biosciences; University of Birmingham, Phenome Centre Birmingham<br>Madhani, Melanie; University of Birmingham, Institute of Cardiovascular Sciences<br>Ives, Natalie; University of Birmingham, Birmingham Clinical Trials Unit; University of Birmingham, Institute of Applied Health Research<br>Kirchhof, Paulus; University of Birmingham, Institute of Cardiovascular Sciences; UKE, Cardiology, University Heart and Vascular Center<br>Jones, Timothy; Birmingham Children's Hospital, Paediatric Cardiac Surgery; University of Birmingham, Institute of Cardiovascular Sciences
Keywords:	Congenital heart disease < CARDIOLOGY, Paediatric cardiac surgery < PAEDIATRIC SURGERY, Clinical trials < THERAPEUTICS

SCHOLARONE™
Manuscripts

I, the Submitting Author has the right to grant and does grant on behalf of all authors of the Work (as defined in the below author licence), an exclusive licence and/or a non-exclusive licence for contributions from authors who are: i) UK Crown employees; ii) where BMJ has agreed a CC-BY licence shall apply, and/or iii) in accordance with the terms applicable for US Federal Government officers or employees acting as part of their official duties; on a worldwide, perpetual, irrevocable, royalty-free basis to BMJ Publishing Group Ltd ("BMJ") its licensees and where the relevant Journal is co-owned by BMJ to the co-owners of the Journal, to publish the Work in this journal and any other BMJ products and to exploit all rights, as set out in our licence.

The Submitting Author accepts and understands that any supply made under these terms is made by BMJ to the Submitting Author unless you are acting as an employee on behalf of your employer or a postgraduate student of an affiliated institution which is paying any applicable article publishing charge ("APC") for Open Access articles. Where the Submitting Author wishes to make the Work available on an Open Access basis (and intends to pay the relevant APC), the terms of reuse of such Open Access shall be governed by a Creative Commons licence – details of these licences and which Creative Commons licence will apply to this Work are set out in our licence referred to above.

Other than as permitted in any relevant BMJ Author's Self Archiving Policies, I confirm this Work has not been accepted for publication elsewhere, is not being considered for publication elsewhere and does not duplicate material already published. I confirm all authors consent to publication of this Work and authorise the granting of this licence.

For peer review only

The Bilateral Remote Ischaemic Conditioning in Children (BRICC) trial: protocol for a two-centre, double-blind, randomised controlled trial in young children undergoing cardiac surgery

Nigel E Drury [1,2], Rehana Bi [1,3], Rebecca L Woolley [4,5], John Stickley [1], Kevin P Morris [3,5], James Montgomerie [6], Carin van Doorn [7], Warwick B Dunn [8,9], Melanie Madhani [2], Natalie J Ives [4,5], Paulus Kirchhof [2,10], Timothy J Jones [1,2]

[1] Department of Paediatric Cardiac Surgery, Birmingham Children's Hospital, Birmingham, UK; [2] Institute of Cardiovascular Sciences, University of Birmingham, Birmingham, UK; [3] Department of Paediatric Intensive Care, Birmingham Children's Hospital, Birmingham, UK; [4] Birmingham Clinical Trials Unit, University of Birmingham, Birmingham, UK; [5] Institute of Applied Health Research, University of Birmingham, Birmingham, UK; [6] Department of Paediatric Cardiac Anaesthesia, Birmingham Children's Hospital, Birmingham, UK; [7] Department of Congenital Cardiac Surgery, Leeds Teaching Hospitals NHS Trust, Leeds, UK; [8] School of Biosciences, University of Birmingham, Birmingham, UK; [9] Phenome Centre Birmingham, University of Birmingham, Birmingham, UK; [10] Department of Cardiology, University Heart and Vascular Center, UKE Hamburg, Hamburg, Germany.

Correspondence: Mr Nigel Drury, Department of Paediatric Cardiac Surgery, Birmingham Children's Hospital, Steelhouse Lane, Birmingham, B4 6NH, UK.

tel: +44 121 333 8731, fax: +44 121 333 9441, email: n.e.drury@bham.ac.uk

Word count: 4629 (excluding title page, abstract, references, figure, and table)

Number of figures: 1

Number of tables: 1

Keywords: Remote ischaemic preconditioning, paediatric cardiac surgery, clinical trial, tetralogy of Fallot, ventricular septal defect, cyanosis

For peer review only - http://bmjopen.bmj.com/site/about/guidelines.xhtml

ABSTRACT

Introduction: Myocardial protection against ischaemic-reperfusion injury is a key determinant of heart function and outcome following cardiac surgery in children. However, with current strategies, myocardial injury occurs routinely following aortic cross-clamping, as demonstrated by the ubiquitous rise in circulating troponin. Remote ischaemic preconditioning, the application of brief, non-lethal cycles of ischaemia and reperfusion to a distant organ or tissue, is a simple, low-risk and readily available technique which may improve myocardial protection. The Bilateral Remote Ischaemic Conditioning in Children (BRICC) trial will assess whether remote ischaemic preconditioning, applied to both lower limbs immediately prior to surgery, reduces myocardial injury in cyanotic and acyanotic young children.

Methods and analysis: The BRICC trial is a two-centre, double-blind, randomised controlled trial recruiting up to 120 young children (age 3 months to 3 years) undergoing primary repair of tetralogy of Fallot or surgical closure of an isolated ventricular septal defect. Participants will be randomised in a 1:1 ratio to either bilateral remote ischaemic preconditioning (3 x 5-minute cycles) or sham immediately prior to surgery, with follow-up until discharge from hospital or 30 days, whichever is sooner. The primary outcome is reduction in area under the time-concentration curve for high-sensitivity troponin-T release in the first 24 hours after aortic cross-clamp release. Secondary outcome measures include peak hs-troponin-T, vasoactive inotrope score, arterial lactate, and central venous oxygen saturations in the first 12 hours, and lengths of stay in the paediatric intensive care unit and the hospital.

Ethics and dissemination: The trial was approved by the West Midlands-Solihull NHS Research Ethics Committee (16/WM/0309) on 5 August 2016. Findings will be disseminated to the academic community through peer-reviewed publications and presentation at national & international meetings. Parents will be informed of the results through a newsletter in conjunction with a local charity.

For peer review only

For peer review only - http://bmjopen.bmj.com/site/about/guidelines.xhtml

Trial registration: ISRCTN12923441, registered May 2016.

Strengths and limitations of this study

- This is the first randomised controlled trial to evaluate the efficacy of bilateral remote ischaemic preconditioning, applied simultaneously to both lower limbs to provide a more intense stimulus in young patients undergoing surgery.
- It is also the first multi-centre cardiac surgical trial in children in the UK.
- We will exclude neonates, in whom preconditioning may be harmful, and avoid the use of propofol anaesthesia, which is thought to interfere with the preconditioning pathway.
- A potential limitation is if exposure to cyanosis in those with tetralogy of Fallot has already had a preconditioning effect, this could attenuate the effect of the intervention.
- The effect of the intervention may also be concealed if right ventricular incision, muscle resection, or outflow tract stent removal significantly increase troponin release in patients with tetralogy of Fallot above that associated with ischaemia-reperfusion.

For peer review only

INTRODUCTION

Myocardial protection

During most surgery for congenital heart disease, it is necessary to stop the heart, allowing access to a still and bloodless field to enable repair of intracardiac defects. Cardioplegia and hypothermia have been fundamental to arresting the heart and protecting against ischaemia-reperfusion (IR) injury during surgery for over 40 years and are used in approx. 3,500 cardiac surgical operations in children in the UK & Ireland each year [1]. However, the developing myocardium exhibits marked differences in metabolism from the adult heart [2] and as current techniques for cardioprotection were developed in adults, they may not be optimal for young children [3,4]. Myocardial injury still occurs routinely following aortic cross-clamping in children [2,5], with IR leading to a degree of contractile impairment which may manifest as low cardiac output and require inotropic support in the early postoperative period. This is a major cause of morbidity and death in the early postoperative period [6,7] and children with preoperative cyanosis are more vulnerable to the effects of IR than acyanotic children [8,9]. Postoperative elevation of circulating troponin is a biomarker of myocardial injury and has been shown to strongly correlate with clinical outcomes including level of inotropic support, duration of ventilation, ventricular dysfunction and early death [5,10]; consequently, it is the most common primary outcome measure in clinical trials of cardioprotection in children [11]. Myocardial protection therefore is a key determinant of heart function and outcome following cardiac surgery.

Remote Ischaemic Preconditioning

Remote ischaemic preconditioning (RIPC) involves the application of brief, non-lethal cycles of ischaemia and reperfusion to a distant organ or tissue, such as a limb, to induce protection against subsequent myocardial IR injury [12]. There are thought to be two phases of cardioprotection: a first window with an immediate effect lasting several hours, and a

For peer review only - http://bmjopen.bmj.com/site/about/guidelines.xhtml

second window which appears around 12-24 hours and lasts for 48-72 hours [13]. The stimulus has traditionally been applied to the upper arm (adults) or thigh (children) using a blood pressure cuff inflated to above systolic pressure [14]. The promise of this simple, low-risk, inexpensive and readily available technique as an adjunct to current methods for myocardial protection has prompted numerous trials in adults [15-20] and children [21-28] but with mixed results. A meta-analysis suggested that RIPC reduces myocardial injury in both adult and paediatric cardiac surgery [29], but subsequently two large multi-centre trials in adults failed to show benefit in either composite cardiovascular endpoints or troponin release [19,20]; both have been criticised for using propofol anaesthesia after it had been suggested to interfere with the preconditioning pathway [30,31].

Cheung et al first demonstrated reductions in troponin release and perioperative inotropic requirements in a heterogeneous cohort of children, most of whom had either tetralogy of Fallot or ventricular septal defect (VSD) [21]. Several studies have found improved myocardial protection in infants and young children undergoing tetralogy of Fallot repair [28] or VSD closure [22,23], whilst others have found no benefit [24,25] and suggested that preoperative cyanosis may have already up-regulated pro-survival pathways [25]. The only trial in cyanosed neonates found no benefit, citing young age, myocardial immaturity and chronic hypoxaemia as potential conflicting factors [26]; animal models have also suggested that preconditioning may have no effect [32] or even be harmful [33] to the immature myocardium. To date, no clinical trials have compared the effects of RIPC in patients with or without chronic cyanosis and its impact on preconditioning remains uncertain [34].

In the largest paediatric trial to date, McCrindle et al. found no benefit in clinical outcomes, physiological markers or subgroup analyses in a mixed cohort of 299 children [27] and proposed that better than expected outcomes in the control group, heterogeneity of underlying conditions, and use of propofol may have affected their findings. Failure to elicit a stimulus may also have been a key factor; manual inflation of the cuff to just 15mmHg above systolic pressure may have led to periods of subclinical reperfusion and abolition of any

For peer review only - http://bmjopen.bmj.com/site/about/guidelines.xhtml

preconditioning response. A recent meta-analysis in children determined that RIPC has a cardioprotective effect, with reduced troponin release, lower inotrope scores and reduced paediatric intensive care unit (PICU) stay following surgery [35] but was unable to include the largest trial in most analyses due to a lack of suitable published data.

Rationale

In this trial, we will test the hypothesis that adequately delivered RIPC is cardioprotective in young children undergoing primary repair of tetralogy of Fallot or closure of an isolated VSD, the two most common congenital heart defects requiring surgery [1]. The design will enable evaluation of the effects of RIPC in children with and without preoperative cyanosis [34]; most patients with tetralogy of Fallot have chronic hypoxaemia whilst those with a VSD are not usually cyanotic and both groups undergo surgery at a similar age. We will use a more intensive two cuff technique [18], applying a concurrent stimulus to both lower limbs to compensate for the lower skeletal muscle mass in young children. We will address methodological concerns by using a pressure-controlled tourniquet system set to at least 50mmHg above systolic pressure [27], avoiding propofol anaesthesia [30,31], and not enrolling neonates or other infants less than three months old [26]. We will only seek to exploit the first window of preconditioning, performing the intervention under general anaesthesia prior to sternotomy, as the second window would require RIPC at least 12 hours prior to surgery [13] which may be logistically challenging, distressing to the child and their parents, and lead to incomplete intervention or withdrawal. Finally, this trial will be the first multi-centre cardiac surgical trial in children in the UK [36] and act as a primer for the development of a network for the design and conduct of multi-centre phase III trials in paediatric cardiac surgery in the UK and Ireland.

For peer review only - http://bmjopen.bmj.com/site/about/guidelines.xhtml

METHODS AND ANALYSIS

Design

The Bilateral Remote Ischaemic Conditioning in Children (BRICC) trial is a two-centre, double-blind, parallel arm, randomised controlled trial to investigate the effects of RIPC and the impact of cyanosis on myocardial protection in young children undergoing elective cardiac surgery. It will be conducted through the Birmingham Clinical Trials Unit (BCTU), a UKCRC-registered clinical trials unit with expertise in surgical and paediatric trials.

Inclusion and exclusion criteria

Inclusion criteria: All infants and young children, aged 3 months to 3 years at the time of surgery, undergoing either primary repair of tetralogy of Fallot or surgical closure of a VSD, with or without concomitant atrial septal defect (ASD) closure or pulmonary artery repair/augmentation, at Birmingham Children's Hospital or Leeds Children's Hospital will be included. Only patients with the most common form of tetralogy of Fallot will be included; variants such as absent pulmonary valve syndrome, pulmonary atresia with major aortopulmonary collateral arteries, or with an atrioventricular septal defect will not be included.

Exclusion criteria: The following children will be excluded from the study:

- Those requiring an additional procedure (other than ASD closure or pulmonary artery repair/augmentation) at the time of primary repair eg. mitral repair, aortic arch repair.
- Those with significant airway or parenchymal lung disease, bleeding disorder or a recent ischaemic event.
- Those who have undergone a previous cardiac surgical procedure with cardioplegic arrest.
- Those presenting in a critical condition and requiring emergency surgery.

For peer review only - http://bmjopen.bmj.com/site/about/guidelines.xhtml

- Those for whom the parents are unwilling or unable to give informed consent.

Recruitment

Both tetralogy of Fallot and VSD are congenital heart defects that usually present with gradual onset of symptoms such as failure to thrive, difficulty feeding, dyspnoea or cyanosis. The referral pathway is therefore predictable with most children undergoing elective surgery following a period of medical therapy to allow them to grow; some children may require a palliative procedure prior to repair, notably right ventricular outflow tract (RVOT) stenting for cyanosis in tetralogy of Fallot [37], or pulmonary artery banding to reduce pulmonary overcirculation with an unrestrictive VSD. All eligible patients will be identified from the multi-disciplinary team meeting, surgical clinics or waiting lists and their parents approached to ascertain interest in the trial. They will be provided with a Parent/Guardian Information Sheet (appendix A-B) either in the clinic/ward or sent in the post and given at least 24 hours to consider their child's participation and ask questions. Written informed consent will be obtained by a Consultant Surgeon prior to enrolment (appendix C-D). The participant pathway through the trial is shown in figure 1.

For peer review only

Randomisation and blinding

On the day of surgery, participants will be randomised in a 1:1 ratio to either RIPC or sham procedure using a secure online randomisation system, with a minimisation algorithm incorporating the following factors:

- congenital heart defect: tetralogy of Fallot or VSD,
- presence of an RVOT stent in patients with tetralogy of Fallot, and
- surgical centre: Birmingham or Leeds.

For peer review only - http://bmjopen.bmj.com/site/about/guidelines.xhtml

To avoid any possibility of the allocation becoming predictable, a random element will be included in the algorithm. If online randomisation is unavailable, a telephone helpline with emergency paper randomisation will be used. An independent healthcare professional, trained and competent in delivering the trial intervention, will perform the randomisation and administer the allocated treatment according to a standard operating procedure; the research nurse, surgical, anaesthetic, perfusion and PICU teams involved in the child's care will remain blinded to group allocation throughout the trial.

For peer review only

Treatment arms

Intervention arm: After induction of anaesthesia but prior to sternotomy, the treatment group will receive RIPC induced by three cycles of 5-minutes ischaemia and 5-minutes reperfusion [38]. Ischaemia will be induced simultaneously in both lower limbs using the PTSii system (Delfi Medical Innovations, Vancouver), a state-of-the-art digital tourniquet with precise control of occlusion pressure. Age-appropriate PediFit cuffs, with contour limb protection sleeves, will be placed around both thighs and inflated to at least 50mmHg above systolic pressure measured via the arterial line during the ischaemia phase of each cycle. If one lower limb is unavailable, eg. required for vascular access during the intervention period, one cuff may be placed on the upper arm instead. In addition, a dummy limb will be placed between the patient's legs to maintain blinding (see control arm below). Continual loss of arterial flow will be confirmed by distal pulse oximetry during each limb occlusion cycle, visible only to the person applying the intervention [25]; if the distal trace is not rapidly lost, the cuff will be tightened or the inflation pressure increased to achieve arterial occlusion. If pulse oximetry is not available, a clinical assessment will be made to determine whether there is loss of arterial flow (decreased lower limb temperature to touch, marked prolongation of capillary refill time) and reperfusion (increased lower limb temperature +/- blushing) during each cycle. Once the intervention has begun, each cuff must be kept on the

same limb to ensure repeated doses of IR to the same muscle mass. Blinding will be maintained by covering the child with a surgical drape from the nipples downwards throughout the period of cuff application, intervention, and removal.

Control arm: Contour limb protection sleeves will be placed around both thighs but the PediFit cuffs will be attached to the dummy limb (43x300mm polyethylene tubing) placed between the patient's legs. Three sham inflation-deflation cycles will be performed using the PTSii system. Pulse oximetry monitoring will be reviewed by the person applying the intervention only, but no loss of trace will be observed during the cycles. As above, the child will be covered with a surgical drape to maintain blinding before, during and after the sham intervention.

Adherence to treatment will be defined as receiving the allocated treatment, and in the intervention arm, with loss of arterial flow (pulse oximetry or clinical assessment, if required) during each period of limb ischaemia.

Common aspects of care

Anaesthesia: Anaesthesia will be conducted at the discretion of the consultant anaesthetist and involve a balanced technique using volatile and intravenous anaesthesia and adjuncts, opioid pain relief and muscle relaxants, within the limits of the protocol. Propofol will not be used for induction or maintenance of anaesthesia; isoflurane will be the preferred volatile anaesthetic agent and end-tidal partial pressure will be recorded at the end of RIPC administration. Phenylepherine will be used for vasoconstriction, as required. Routine monitoring will include continuous invasive arterial and central venous pressures, other cardiac output variables, urine output, blood gas analysis and near-patient clotting profile [39]. Systemic anticoagulation will be achieved with heparin prior to institution of cardiopulmonary bypass (CPB) and reversed with protamine after the termination of CPB.

For peer review only - http://bmjopen.bmj.com/site/about/guidelines.xhtml

Surgery & Perfusion: Repair of the congenital heart defect(s) will be performed following best clinical practice. After transfer to the operating room, the surgical checklist will be completed, the patient prepped and draped, and the chest opened through a median sternotomy. Standardised cardiopulmonary bypass will be established between the vena cavae and the ascending aorta with moderate hypothermia. An aortic cross-clamp will be applied to the proximal ascending aorta with intermittent antegrade cold cardioplegia given via the aortic root for myocardial protection; patients undergoing VSD closure will usually receive a single dose, whilst those with tetralogy of Fallot will typically require an additional dose. Removal of the aortic cross-clamp with myocardial reperfusion will be considered as time zero for the recording of postoperative events. Following completion of the repair and rewarming, CPB will be weaned and discontinued. In the event of difficulty separating from bypass or marked haemodynamic instability, subjective and objective measures of ventricular function will be obtained, and inotropic support instituted at the discretion of the blinded operating team. Once haemodynamic stability and haemostasis have been achieved, the chest will be closed at the discretion of the surgical team and the patient transferred to the PICU. Standard postoperative care will proceed with anticipated removal of the arterial line at 12 hours following surgery, removal of the central line at 24 hours, and transfer to the ward once routine PICU discharge criteria have been met. All decisions regarding escalation of therapy will be made by the blinded clinical team responsible for the care of the child without influence from the researchers.

For peer review only

Trial investigations

The schedule for the intervention and collection of outcome data, blood and tissue samples is shown in table 1.

Data collection: Clinical data will be collected by the Research Nurse before, during and after surgery. Inotrope usage in the first 12 hours will be used to generate a vasoactive inotrope

For peer review only - http://bmjopen.bmj.com/site/about/guidelines.xhtml

score (VIS) (μg/kg/min) [40,41]. Arterial lactate and central venous oxygen saturations will be recorded prior to surgery and at 3, 6, 9 and 12 hours. Length of stay in PICU (hours) and hospital (days) following surgery will be documented. Preoperative haematocrit and resting oxygen saturations in air will be used as markers of the degree of exposure to cyanosis. In Birmingham only, cardiac output will be measured over the first 12 hours following reperfusion using ICON (Osypka Medical, Berlin), a non-invasive technique for electrical velocimetry which has been validated in young children [42-44].

Blood samples: Blood will be drawn from indwelling arterial or central venous lines at baseline (after induction of anaesthesia but prior to sternotomy) and at 3, 6, 12 and 24 hours after reperfusion. Plasma samples for hs-troponin-T (Elecsys Tn-T HS, Roche, Basel) will be collected in paediatric lithium heparin tubes, centrifuged, split into two aliquots and stored at -80°C in remotely-monitored freezers at each site until transfer for analysis at one of two core labs (Sandwell General Hospital, Birmingham or Russells Hall Hospital, Dudley). Samples will be analysed in batches approximately every eight months so that data on the primary outcome will be available to the Data Monitoring Committee prior to each meeting.

Tissues samples: In Birmingham only, myocardial biopsies will be obtained for a metabolic sub-study. Right atrial samples will be taken soon after aortic cross-clamping (onset ischaemia) and just before its release (late ischaemia) to assess metabolic changes in the myocardium during the period of ischaemia. In a subset of patients with tetralogy of Fallot, several samples of hypertrophic septoparietal trabeculae of the right ventricular infundibulum will be obtained at various points during ischaemia, whenever routinely resected. Specimens will be briefly washed in saline, promptly snap-frozen in liquid nitrogen and stored at -80°C until transfer to the Phenome Centre Birmingham for metabolic phenotyping. Analysis of these samples is exploratory and will follow a separate analytical plan (see sub-study below).

For peer review only

Outcome measures and follow-up

Primary outcome: Reduction in area under the time-concentration curve (AUC) for high-sensitivity troponin-T release in the first 24 hours after aortic cross-clamp release (reperfusion) as a marker of myocardial injury.

Secondary outcomes

- Peak hs-troponin-T in the first 12 hours
- Total vasoactive inotrope score in the first 12 hours
- Arterial lactate and central venous oxygen saturations in the first 12 hours
- Length of postoperative stay in the PICU
- Length of postoperative stay in the hospital

Exploratory outcome: Cardiac index in the first 12 hours measured using ICON (Birmingham only).

Follow-up: until discharge from hospital or 30 days, whichever is sooner.

Analysis

Sample size: It is hypothesised that RIPC will reduce the AUC for hs-troponin-T release in the first 24 hours compared with controls, but that exposure to hypoxaemia may impact on this reduction. The sample size proposed here will be sufficient to detect a 35% reduction in postoperative troponin release, assuming a mean release of 350 µg/L/h in the control group compared with 228 µg/L/h in the RIPC group (extrapolated from the similarly mixed cohort of hypoxic and non-hypoxic children in Toronto [21]), with a variability of 220 µg/L/h [24]. A sample size of at least 52 children per treatment group is needed to have a power of 80% and a significance level of 0.05 (2-sided). We therefore will recruit at least 104 children (up to 120 children to allow for dropouts) randomised in a 1:1 ratio between RIPC and control.

For peer review only - http://bmjopen.bmj.com/site/about/guidelines.xhtml

For peer review only

Expected recruitment rate: The paediatric cardiac surgery units in Birmingham and Leeds are ideally placed to conduct clinical trials. Over the preceding three years, 99-135 children per annum have undergone surgical repair of either tetralogy of Fallot (mean 50) or VSD (mean 69) across the two sites [1]. The only previous interventional trial in cardiac surgery at Birmingham Children's Hospital recruited 22 (79%) of the 28 patients approached [45]. None of the other UK paediatric cardiac surgery RCTs have reported recruitment rates [36] but our predictions are comparable to those obtained from similar trials in North America which ranged from 62% to 84% [27,36,46]. We will maintain a screening log to document exclusions and reasons given by parents who decline to participate; this will be available to the Trial Management Committee who will monitor recruitment targets and advise on any changes to the protocol.

Statistical analysis: Analysis of the main outcome measures will be performed according to the intention-to-treat principle and any non-adherence to the allocated group documented. The primary analysis will assess whether RIPC reduces AUC for troponin release in the first 24 hours compared with control. The primary outcome measure will be calculated using the trapezoidal method and presented as an adjusted mean difference between groups along with the 95% confidence interval estimated using a linear regression model (adjusting for the minimisation variables and baseline troponin). For the secondary outcomes, continuous data items (eg. peak troponin) will also be analysed using a linear regression model. Continuous outcomes measured across more than three time points (eg. arterial lactate and central venous oxygen saturations) will be analysed using mixed effect repeated measures models. Time to event data outcomes will be analysed using a Cox regression model. Test of interactions will be employed to assess whether there is evidence that the treatment effect differs between cyanotic and acyanotic patients. P-values will be reported from two-sided tests at the 5% significance level. A detailed statistical analysis plan will be developed and approved prior to database lock. The Chief Investigator and trial statisticians will have access to the final trial dataset.

For peer review only - http://bmjopen.bmj.com/site/about/guidelines.xhtml

Monitoring

Assessment and management of risk: No adverse events directly attributed to the application of a tourniquet cuff during RIPC were identified in a meta-analysis of 1,762 adults and children undergoing cardiac surgery in 25 trials [29] nor in any of the notable trials published since [19,20,27,28]. Risk to participants therefore is deemed to be minimal and the trial is categorised as type A: no higher than the risk of standard medical care. In the event of concern, parents will be signposted to their cardiac specialist nurse, their General Practitioner, or the hospital Patient Advice Liaison Service, as appropriate.

Trial Management Committee: The trial will be overseen by a committee meeting approximately every four months during the trial. It will comprise clinicians, trialists and scientists involved in the set-up and running of the trial including representation from both trial sites. During recruitment, the protocol may be reviewed considering achievement of recruitment targets, evidence from new publications, and feedback from parents approached for the trial; ethical approval for amendments to the protocol will be sought, as required.

Data Monitoring Committee: An independent Data Monitoring Committee will meet approximately every eight months during recruitment to review efficacy and safety data, according to a predefined charter (appendix E). Members are an academic consultant cardiac surgeon as chair, a consultant in paediatric cardiac intensive care, and a statistician. Analysis of hs-troponin-T for the primary outcome will be performed in batches prior to each meeting and all unblinded safety and efficacy data made available to the committee.

Safety reporting: Adverse events will be recorded and reported in accordance with the sponsor's Code of Practice for Research. Participants in the study are undergoing open heart surgery and therefore adverse events are anticipated. The following serious adverse events will be reviewed by the Chief Investigator and reported to the sponsor within 48 hours of identification: death; requirement for extracorporeal life support; evidence of a major neurological event; and need for further surgery in the early post-operative period.

For peer review only - http://bmjopen.bmj.com/site/about/guidelines.xhtml

For peer review only

Data collection and management: All data will be entered onto the BRICC trial database, a password protected electronic database held on secure University of Birmingham servers for trial data with access limited to BCTU members of staff working on the trial. All paper case report forms will be stored securely in the Research offices at Birmingham Women's and Children's NHS Foundation Trust and Leeds Teaching Hospitals NHS Trust. Data will be semi-anonymised by removing non-essential potentially identifiable patient information; blood and tissue samples will be labelled with the unique trial ID number, date, and time of collection. Adherence to trial processes will be audited by the independent Clinical Research Compliance team at the University of Birmingham.

Sub-studies

Metabolic phenotyping: No study in children has previously examined the impact of RIPC on myocardial metabolism or its interaction with chronic hypoxaemia. Therefore, building on metabolic phenotyping in animal models of IR injury [47], we will analyse intraoperative biopsies to identify changes in myocardial metabolic pathways that occur during ischaemia. In brief, tissue extracts will be analysed using ultra high performance liquid chromatography-mass spectrometry (UHPLC-MS) in two independent discovery and validation phases. Two complementary assays will be applied, (1) HILIC assay to study water-soluble metabolites, including those present in glycolysis and the TCA cycle, and (2) C_{18} reversed-phase assay to determine changes in lipids during ischaemia [48]. The eluents from UHPLC columns will be introduced directly into an electrospray Q Exactive Mass Spectrometer (Thermo Scientific, UK) and data acquired in the *m/z* range 70-1000. The impact of RIPC on metabolism and how any changes may be attenuated by preoperative cyanosis, will be assessed through robust statistical analysis using correction for multiple testing and pathway enrichment analysis.

For peer review only - http://bmjopen.bmj.com/site/about/guidelines.xhtml

Qualitative: We will explore parents' perspectives on decision-making about their child's participation in a clinical trial as part of their elective cardiac surgery. Parents of children approached to participate in the trial, both consenters and decliners, will be contacted following surgery and asked to participate in semi-structured interviews which, with written informed consent, will be digitally audio-recorded, intelligently transcribed, and thematically analysed. The findings will enhance our understanding of the factors that influence parents' decision-making and be used to inform the design and conduct of future trials. The BRICC trial is a suitable vehicle for this sub-study as the intervention presents minimal risk, the surgery is performed electively, and the operations included have a low predicted mortality (STAT categories 1-2) [49].

Patient and Public Involvement

Patient and Public Involvement (PPI) has been a central component in the development, conduct and planned reporting of this trial since its inception. Parents of children who had previously undergone cardiac surgery at Birmingham Children's Hospital were contacted through *Young at Heart*, the local children's heart charity. Four parents reviewed the draft parent information sheet and consent form for the trial, making suggestions to improve clarity and readability for a lay audience, which were incorporated into the final versions. The parent information sheets, consent forms and protocol for the qualitative sub-study were also reviewed by the Young Person's Steering Group in the West Midlands. The outcomes of the trial will be communicated by individual parent feedback and a charity newsletter, both of which will be produced in collaboration with the charity and parents. Early user involvement was funded by a bursary from the NIHR Research Design Service West Midlands and all PPI was costed using the INVOLVE Calculator according to the NIHR's Budgeting for Involvement [50].

For peer review only - http://bmjopen.bmj.com/site/about/guidelines.xhtml

ETHICS AND DISSEMINATION

This clinical trial was approved by the West Midlands-Solihull NHS Research Ethics Committee (16/WM/0309) on 5 August 2016 and the NHS Health Research Authority (200876) on 19 August 2016. It is sponsored by the University of Birmingham (RG_14-025, email: researchgovernance@contacts.bham.ac.uk, telephone: +44 (0) 121 415 8011), registered on the NIHR Clinical Research Network portfolio (32330), and approved by the NHS Research & Development departments at Birmingham Children's Hospital (1845) and Leeds Children's Hospital (PA17/67348). Regulatory approval from the Medicines and Healthcare products Regulatory Agency (MHRA) was not required as this trial is not a CTIMP. The first patient was randomised on 24 October 2016 and recruitment is currently ongoing.

Changes to the protocol since original ethical approval

Since the original ethical approval, four substantial amendments to the protocol have been sought and approved with the following significant changes:

- Add 'with/without concomitant pulmonary artery repair/augmentation' to the inclusion criteria, to allow inclusion of those with pulmonary artery disease within the spectrum of tetralogy of Fallot and those with VSD who had previous pulmonary artery banding (December 2016).
- Add Leeds Children's Hospital as the second site and extend the duration of recruitment (February 2018).
- Remove 'known major chromosomal defect' as an exclusion criterion; although originally included as per previous paediatric trials [21,27], following discussion with Prof Andrew Redington (Cincinnati, OH), principal investigator of these trials, it became clear that there was no biological reason relating to RIPC to exclude these patients (February 2018).

For peer review only - http://bmjopen.bmj.com/site/about/guidelines.xhtml

- Add Russells Hall Hospital, Dudley as a second core laboratory to maintain internal validity, as Sandwell General Hospital, Birmingham changed their troponin analysis platform during the trial (November 2019).

Dissemination plan

The findings of the clinical trial and sub-studies will be submitted for presentation at national and international meetings and manuscripts prepared for submission to leading journals. The authorship of the final trial report will include all members of the trial management committee and named collaborators. The anonymised individual participant data collected during the trial will be available on request following publication of the study results.

Parents of children participating in the trial will be informed of the results in writing once data analysis is complete. The local charity Young at Heart will also report the outcomes in their newsletter to reach a wider audience of those affected by congenital heart disease. PPI collaborators will be invited to participate in producing both the parent feedback and charity newsletter.

The first author is Chief Investigator of the trial and takes responsibility for the integrity of this protocol report, which adheres to the SPIRIT recommendations [51]. All authors have read and agree to the manuscript as written.

For peer review only

Abbreviations

ASD	Atrial Septal Defect
AUC	Area Under the time-concentration Curve
BCTU	Birmingham Clinical Trials Unit
BRICC	Bilateral Remote Ischaemic Conditioning in Children
CPB	Cardiopulmonary Bypass
CTIMP	Clinical Trial of an Investigational Medicinal Product
IR	Ischaemia-Reperfusion
NHS	National Health Service
NIHR	National Institute for Health Research
PICU	Paediatric Intensive Care Unit
PPI	Patient and Public Involvement
RCT	Randomised Controlled Trial
RIPC	Remote Ischaemic Preconditioning
RVOT	Right Ventricular Outflow Tract
UHPLC-MS	Ultra High Performance Liquid Chromatography-Mass Spectrometry
VIS	Vasoactive Inotrope Score
VSD	Ventricular Septal Defect

For peer review only

Acknowledgements: We are grateful to the members of the independent Data Monitoring Committee for their guidance and oversight throughout the trial: Prof Gavin J Murphy (Chair), University of Leicester, UK; Dr Katherine L Brown, Great Ormond Street Hospital, London, UK; and Dr Peter Nightingale (Statistician), Queen Elizabeth Hospital Birmingham, UK.

We thank Dr John V Pappachan (Southampton, UK) for guidance on the delivery of RIPC, Prof Andrew N Redington (Cincinnati, OH) for advice on RIPC and the exclusion criteria, and Prof Peter Brocklehurst, Director of BCTU, for his support for the trial. We are grateful to our surgical colleagues in Birmingham: Ms Natasha E Khan, Mr Phil Botha, Prof David J Barron & Dr Adrian Crucean, and Leeds: Mr Osama Jaber, Mr Imre Kassai & Mr Guiseppe Pelella for their input to the evolution of the trial. We thank Dr Oliver Stumper and Dr Anna N Seale, Birmingham for their advice on ICON monitoring and echo imaging, respectively. We thank Matt Hill and Alicia Gill at BCTU for programming and statistical support, respectively, and Collette Spencer for setting up the study in Leeds. We are most grateful to Martina Ponsonby and the trustees of *Young at Heart* for their feedback on the study documents.

Author contributions: NED, MM and TJJ conceptualised the trial. NED, RB, KPM, JM, NJI, PK and TJJ designed the trial with additional critical input from RLW, JS, CVD and MM. NED, RLW and NJI developed the statistical analysis. NED and WBD designed the metabolic phenotyping sub-study. All authors contributed to writing of the paper.

Funding: This work is supported by an Intermediate Clinical Research Fellowship from the British Heart Foundation [FS/15/49/31612] awarded to Nigel Drury. Lay review of the parent information sheets and consent forms was funded by a Patient & Public Involvement Bursary Award [RDS/WM-1318] from the NIHR Research Design Service in the West Midlands. The Institute of Cardiovascular Sciences is supported by an Accelerator Award from the British Heart Foundation [AA/18/2/34218]. Phenome Centre Birmingham was constructed through a grant from the Medical Research Council in the UK [MR/M009157/1]. Neither the sponsor nor funders had any role in the design of this study and will not have any role during its

For peer review only

execution, analyses, interpretation of the data, or decision to submit the results for publication.

Competing interests: None declared.

Ethics approval: West Midlands-Solihull National Health Service Research Ethics Committee (16/WM/0309) on 5 August 2016. This manuscript is based on protocol v1.8 dated 22 November 2019, which was approved by the ethics committee on 5 December 2019.

For peer review only

For peer review only - http://bmjopen.bmj.com/site/about/guidelines.xhtml

REFERENCES

1. NICOR National Institute for Cardiovascular Outcomes Research, UCL. Congenital Heart Disease. https://nicor4.nicor.org.uk/CHD/an_paeds.nsf/vwContent/home [accessed March 17, 2020]

2. Doenst T, Schlensak C, Beyersdorf F. Cardioplegia in pediatric cardiac surgery: do we believe in magic? *Ann Thorac Surg* 2003; 75: 1668-77.

3. del Nido PJ, Mickle DA, Wilson GJ, Benson LN, Weisel RD, Coles JG, Trusler GA, Williams WG. Inadequate myocardial protection with cold cardioplegia arrest during repair of tetralogy of Fallot. *J Thorac Cardiovasc Surg* 1988; 95: 223-9.

4. Allen BS. Pediatric myocardial protection: where do we stand? *J Thorac Cardiovasc Surg* 2004; 128: 11-13.

5. Mildh LH, Pettilä V, Sairanen HI, Rautiainen PH. Cardiac Troponin T levels for risk stratification in pediatric open heart surgery. *Ann Thorac Surg* 2006; 82: 1643-9.

6. Ma M, Gauvreau K, Allan CK et al. Causes of death after congenital heart surgery. *Ann Thorac Surg* 2007; 83: 1438-45.

7. Gaies M, Pasquali SK, Donohue JE et al. Seminal postoperative complications and mode of death after pediatric cardiac surgical procedures. *Ann Thorac Surg* 2016; 102: 628-35.

8. Imura H, Caputo M, Parry A, Pawade A, Angelini GD, Suleiman MS. Age-dependent and hypoxia-related differences in myocardial protection during paediatric open-heart surgery. *Circulation* 2001; 103: 1551-6.

9. Najm HK, Wallen WJ, Belanger MP, Williams WG, Coles JG, Van Arsdell GS, Black MD, Boutin C, Wittnich C. Does the degree of cyanosis affect myocardial adenosine triphosphate levels and function in children undergoing surgical procedures for congenital heart disease? *J Thorac Cardiovasc Surg* 2000; 119: 515-24.

For peer review only - http://bmjopen.bmj.com/site/about/guidelines.xhtml

10. Immer FF, Stocker F, Seiler AM et al. Troponin-I for prediction of early postoperative course after pediatric cardiac surgery. *J Am Coll Cardiol* 1999; 33: 1719-23.

11. Drury NE, Yim I, Patel AJ et al. Cardioplegia in paediatric cardiac surgery: a systematic review of randomized controlled trials. *Interact Cardiovasc Thorac Surg* 2019; 28: 144-150.

12. Hausenloy DJ, Yellon DM. Remote ischaemic preconditioning: underlying mechanisms and clinical application. *Cardiovasc Res* 2008; 79: 377-86.

13. Hausenloy DJ, Yellon DM. The Second Window of Preconditioning (SWOP): where are we now? *Cardiovasc Drugs Ther* 2010; 24: 235-54.

14. Kharbanda RK, Mortensen UM, White PA, Kristiansen SB, Schmidt MR, Hoschtitzky JA, Vogel M, Sorensen K, Redington AN, MacAllister R. Transient limb ischemia induces remote ischemic preconditioning in vivo. *Circulation* 2002; 106: 2881-3.

15. Hausenloy DJ, Mwamure PK, Venugopal V, Harris J, Barnard M, Grundy E, Ashley E, Vichare S, Di Salvo C, Kolvekar S, Hayward M, Keogh B, MacAllister RJ, Yellon DM. Effect of remote ischaemic preconditioning on myocardial injury in patients undergoing coronary artery bypass graft surgery: a randomised controlled trial. *Lancet* 2007; 370: 575-9.

16. Rahman IA, Mascaro JG, Steeds RP, Frenneaux MP, Nightingale P, Gosling P, Townsend P, Townend JN, Green D, Bonser RS. Remote ischaemic preconditioning in human coronary artery bypass surgery: from promise to disappointment? *Circulation* 2010; 122 (11 Suppl): S53-9.

17. Thielmann M, Kottenberg E, Kleinbongard P, Wendt D, Gedik N, Pasa S, Price V, Tsagakis K, Neuhauser M, Peters J, Jakob H, Heusch G. Cardioprotective and prognostic effects of remote ischaemic preconditioning in patients undergoing coronary artery bypass surgery: a single-centre randomised, double-blind, controlled trial. *Lancet* 2013; 382: 597-604.

For peer review only

For peer review only - http://bmjopen.bmj.com/site/about/guidelines.xhtml

18. Candilio L, Malik A, Ariti C, Barnard M, Di Salvo C, Lawrence D, Hayward M, Yap J, Roberts N, Sheikh A, Kolvekar S, Hausenloy DJ, Yellon DM. Effect of remote ischaemic preconditioning on clinical outcomes in patients undergoing cardiac bypass surgery: a randomised controlled clinical trial. *Heart* 2015; 101: 185-92.

19. Meybohm P, Bein B, Brosteanu O, Cremer J, Gruenewald M, Stoppe C, Coburn M, Schaelte G, Böning A, Niemann B, Roesner J, Kletzin F, Strouhal U, Reyher C, Laufenberg-Feldmann R, Ferner M, Brandes IF, Bauer M, Stehr SN, Kortgen A, Wittmann M, Baumgarten G, Meyer-Treschan T, Kienbaum P, Heringlake M, Schön J, Sander M, Treskatsch S, Smul T, Wolwender E, Schilling T, Fuernau G, Hasenclever D, Zacharowski K. A multicenter trial of remote ischemic preconditioning for heart surgery. *N Engl J Med* 2015; 373: 1397-407.

20. Hausenloy DJ, Candilio L, Evans R, Ariti C, Jenkins DP, Kolvekar S, Knight R, Kunst G, Laing C, Nicholas J, Pepper J, Robertson S, Xenou M, Clayton T, Yellon DM. Remote ischemic preconditioning and outcomes of cardiac surgery. *N Engl J Med* 2015; 373: 1408-17.

21. Cheung MM, Kharbanda RK, Konstantinov IE, Shimizu M, Frndova J, Li J, Holtby HM, Cox PN, Smallhorn JF, Van Arsdell GS, Redington AN. Randomised controlled trial of the effects of remote ischemic preconditioning on children undergoing cardiac surgery: first clinical application in humans. *J Am Coll Cardiol* 2006; 47: 2277-82.

22. Zhou W, Zeng D, Chen R, Liu J, Yang G, Liu P, Zhou X. Limb ischemic preconditioning reduces heart and lung injury after an open heart operation in infants. *Pediatr Cardiol* 2010; 31: 22-29.

23. Luo W, Zhu M, Huang R, Zhang Y. A comparison of cardiac post-conditioning and remote pre-conditioning in paediatric cardiac surgery. *Cardiol Young* 2011; 21: 266-70.

For peer review only

24. Lee JH, Park YH, Byon HJ, Kim HS, Kim Cs, Kim JT. Effect of remote ischaemic preconditioning on ischaemia-reperfusion injury in pulmonary hypertensive infants receiving ventricular septal defect repair. *Br J Anaesth* 2012; 108: 223-8.

25. Pepe S, Liaw NY, Hepponstall H, Sheeran FL, Yong MS, d'Udekem Y, Cheung MM, Konstantinov IE. Effect of remote ischemic preconditioning on phosphorylated protein signalling in children undergoing Tetralogy of Fallot repair: a randomised controlled trial. *J Am Heart Assoc* 2013; 2: e000095.

26. Jones BO, Pepe S, Sheeran FL, Donath S, Hardy P, Shekerdemian L, Penny DJ, McKenzie I, Horton S, Brizard CP, d'Udekem Y, Konstantinov IE, Cheung MM. Remote ischemic preconditioning in cyanosed neonates undergoing cardiopulmonary bypass: a randomized controlled trial. *J Thorac Cardiovasc Surg* 2013; 146: 1334-40.

27. McCrindle BW, Clarizia NA, Khaikin S, Holtby HM, Manlhiot C, Schwartz SM, Caldarone CA, Coles JG, Van Arsdell G, Scherer SW, Redington AN. Remote ischemic preconditioning in children undergoing cardiac surgery with cardiopulmonary bypass: a single-center double-blinded randomized trial. *J Am Heart Assoc* 2014; 3: e000964.

28. Wu Q, Wang T, Chen S, Zhou Q, Li H, Hu N, Feng Y, Dong N, Yao S, Xia Z. Cardiac protective effects of remote ischaemic preconditioning in children undergoing tetralogy of Fallot repair surgery: a randomized controlled trial. *Eur Heart J* 2018; 39: 1028-37.

29. Haji Mohd Yasin NA, Herbison P, Saxena P, Praporski S, Konstantinov IE. The role of remote ischemic preconditioning in organ protection after cardiac surgery: a meta-analysis. *J Surg Res* 2014; 186: 207-16.

30. Kottenberg E, Musiolik, Thielmann M, Jakob H, Peters J, Heusch G. Interference of propofol with signal transducer and activator of transcription 5 activation and cardioprotection by remote ischemic preconditioning during coronary artery bypass grafting. *J Thorac Cardiovasc Surg* 2014; 147: 376-82.

For peer review only - http://bmjopen.bmj.com/site/about/guidelines.xhtml

31. Heusch G, Gersh BJ. ERICCA and RIPHeart: two nails in the coffin for cardioprotection by remote ischemic conditioning? Probably not! *Eur Heart J* 2016; 37: 200-2.

32. Awad WI, Shattock MJ, Chambers DJ. Ischemic preconditioning in immature myocardium. *Circulation* 1998; 98: II206-13.

33. Schmidt MR, Støttrup NB, Michelsen MM, Contractor H, Sørensen KE, Kharbanda RK, Redington AN, Bøtker HE. Remote ischemic preconditioning impairs ventricular function and increases infarct size after prolonged ischemia in the isolated neonatal rabbit heart. *J Thorac Cardiovasc Surg* 2014; 147: 1049-55.

34. Konstantinov IE. Remote ischemic preconditioning in children with cyanotic heart disease: lost in translation? *J Thorac Cardiovasc Surg* 2013; 145: 613-4.

35. Tan W, Zhang C, Liu J, Li X, Chen Y, Miao Q. Remote Ischemic Preconditioning has a Cardioprotective Effect in Children in the Early Postoperative Phase: A Meta-Analysis of Randomized Controlled Trials. *Pediatr Cardiol* 2018; 39: 617-26.

36. Drury NE, Patel AJ, Oswald NK et al. Randomized controlled trials in children's heart surgery in the 21st century: a systematic review. *Eur J Cardiothorac Surg* 2018; 53: 724-31.

37. Stumper O, Ramchandani B, Noonan P, Mehta C, Bhole V, Reinhardt Z, Dhillon R, Miller P, de Giovanni J. Stenting of the right ventricular outflow tract. *Heart* 2013; 99: 1603-8.

38. Pickard JM, Bøtker HM, Crimi G, Davidson B, Davidson SM, Dutka D, Ferdinandy P, Ganske R, Garcia-Dorado D, Giricz Z, Gourine AV, Heusch G, Kharbanda R, Kleinbongard P, MacAllister R, McIntyre C, Meybohm P, Prunier F, Redington A, Robertson NJ, Suleiman MS, Vanezis A, Walsh S, Yellon DM, Hausenloy DJ. Remote ischemic preconditioning: from experimental observation to clinical application: report from the 8th biennial Hatter Cardiovascular Institute Workshop. *Basic Res Cardiol* 2015; 110: 453.

For peer review only - http://bmjopen.bmj.com/site/about/guidelines.xhtml

39. Checketts MR, Alladi R, Ferguson K, Gemmell L, Handy JM, Klein AA, Love NJ, Misra U, Morris C, Nathanson MH, Rodney GE, Verma R, Pandit JJ. Recommendations for Standards of Monitoring During Anaesthesia and Recovery 2015: Association of Anaesthetists of Great Britain and Ireland. *Anaesthesia* 2016; 71: 85-93.

40. Gaies MG, Gurney JG, Yen AH, et al: Vasoactive-inotropic score as a predictor of morbidity and mortality in infants after cardiopulmonary bypass. *Pediatr Crit Care Med* 2010; 11: 234-8.

41. Gaies MG, Jeffries HE, Niebler RA et al. Vasoactive-inotropic score is associated with outcome after infant cardiac surgery: an analysis from the Pediatric Cardiac Critical Care Consortium and Virtual PICU system registries. *Pediatr Crit Care Med* 2014; 15: 529-37.

42. Norozi K, Beck C, Osthaus WA, Wille I, Wessel A, Bertram H. Electrical velocimetry for measuring cardiac output in children with congenital heart disease. *Br J Anaesth* 2008; 100: 88-94.

43. Schubert S, Schmitz T, Weiss M, Nagdyman N, Huebler M, Alexi-Meskishvili V, Berger F, Stiller B. Continuous, non-invasive techniques to determine cardiac output in children after cardiac surgery: evaluation of transoesophageal Doppler and electric velocimetry. *J Clin Monit Comput* 2008; 22: 299-307.

44. Noonan PM, Viswanathan S, Chambers A, Stumper O. Non-invasive cardiac output monitoring during catheter interventions in patients with cavopulmonary circulations. *Cardiol Young* 2014; 24: 417-21.

45. Swindell CG, Barker TA, McGuirk SP, Jones TJ, Barron DJ, Brawn WJ, Horsburgh A, Willetts RG. Washing of irradiated red blood cells prevents hyperkalaemia during cardiopulmonary bypass in neonates and infants undergoing surgery for complex congenital heart disease. *Eur J Cardiothorac Surg* 2007; 31: 659-64.

For peer review only - http://bmjopen.bmj.com/site/about/guidelines.xhtml

46. Ohye RG, Sleeper LA, Mahony L, Newburger JW, Pearson GD, Lu M, Goldberg CS, Tabbutt S, Frommelt PC, Ghanayem NS, Laussen PC, Rhodes JF, Lewis AB, Mital S, Ravishankar C, Williams IA, Dunbar-Masterson C, Atz AM, Colan S, Minich LL, Pizarro C, Kanter KR, Jaggers J, Jacobs JP, Krawczeski CD, Pike N, McCrindle BW, Virzi L, Gaynor JW for the Pediatric Heart Network Investigators. Comparison of shunt type in the Norwood procedure for single ventricle lesions. *N Engl J Med* 2010; 362: 1980-92.

47. Chouchani ET, Pell VR, Gaude E, Aksentijević D, Sundier SY, Robb EL, Logan A, Nadtochiy SM, Ord EN, Smith AC, Eyassu F, Shirley R, Hu CH, Dare AJ, James AM, Rogatti S, Hartley RC, Eaton S, Costa AS, Brookes PS, Davidson SM, Duchen MR, Saeb-Parsy K, Shattock MJ, Robinson AJ, Work LM, Freeza C, Krieg T, Murphy MP. Ischaemic accumulation of succinate controls reperfusion injury through mitochondrial ROS. *Nature* 2014; 515: 431-5.

48. Gehmlich K, Dodd MS, Allwood JW, Kelly M, Bellahcene M, Lad HV, Stockenhuber A, Hooper C, Ashrafian H, Redwood CS, Carrier L, Dunn WB. Changes in the cardiac metabolome caused by perhexiline treatment in a mouse model of hypertrophic cardiomyopathy. *Mol Biosyst* 2015; 11: 564-73.

49. O'Brien SM, Clarke DR, Jacobs JP, Jacobs ML, Lacour-Gayet FG, Pizarro C, Welke KF, Maruszewski B, Tobota Z, Miller WJ, Hamilton L, Peterson ED, Mavroudis C, Edwards FH. An empirically based tool for analyzing mortality associated with congenital heart surgery. *J Thorac Cardiovasc Surg* 2009; 138: 1139-53.

50. Mental Health Research Network and INVOLVE. *Budgeting for involvement: Practical advice on budgeting for actively involving the public in research studies*. Mental Health Research Network, London and INVOLVE, Eastleigh, 2013.

51. Chan A-W, Tetzlaff JM, Altman DG, Laupacis A, Gøtzsche PC, Krleža-Jerić K, Hróbjartsson A, Mann H, Dickersin K, Berlin J, Doré C, Parulekar W, Summerskill W, Groves

For peer review only - http://bmjopen.bmj.com/site/about/guidelines.xhtml

T, Schulz K, Sox H, Rockhold FW, Rennie D, Moher D. SPIRIT 2013 Statement: Defining standard protocol items for clinical trials. *Ann Intern Med* 2013; 158: 200-7.

For peer review only

Appendices

Appendix A: Parent/guardian information leaflet: Birmingham, v1.9 dated 22 November 2019

Appendix B: Parent/guardian information leaflet: Leeds, v1.9 dated 22 November 2019

Appendix C: Parent/guardian consent form: Birmingham, v1.9 dated 22 November 2019

Appendix D: Parent/guardian consent form: Leeds, v1.9 dated 22 November 2019

Appendix E: Data Monitoring Committee charter, v1.3 dated 8 June 2018

Figure legends

Figure 1. Participant pathway from screening to end of follow-up.

PIS, parent information sheet; RA, right atrium; RIPC, remote ischaemic preconditioning; RV, right ventricle.

For peer review only

For peer review only - http://bmjopen.bmj.com/site/about/guidelines.xhtml

Table 1. Schedule of events: intervention, outcome data, blood, and tissue samples.

	Pre-operative	Pre-sternotomy	Intraoperative			On PICU admission	Time since aortic cross-clamp removal (hours)					Daily until discharge	Hospital discharge
			Onset ischaemia	During ischaemia	Late ischaemia		3	6	9	12	24		
Screening & consent	x												
Randomisation	x												
Clinical baseline data	x												
RIPC or sham intervention		x											
Blood for hs-troponin-T		x					x	x		x	x		
Arterial/venous blood gases		x					x	x	x	x			
Right atrium biopsies			x		x								
Right ventricle biopsies				x									
Clinical outcome data						x				x	x	x	x
Cardiac index (BCH only)						x	x	x	x	x			

BCH, Birmingham Children's Hospital; PICU, paediatric intensive care unit; RIPC, remote ischaemic preconditioning.

For peer review only

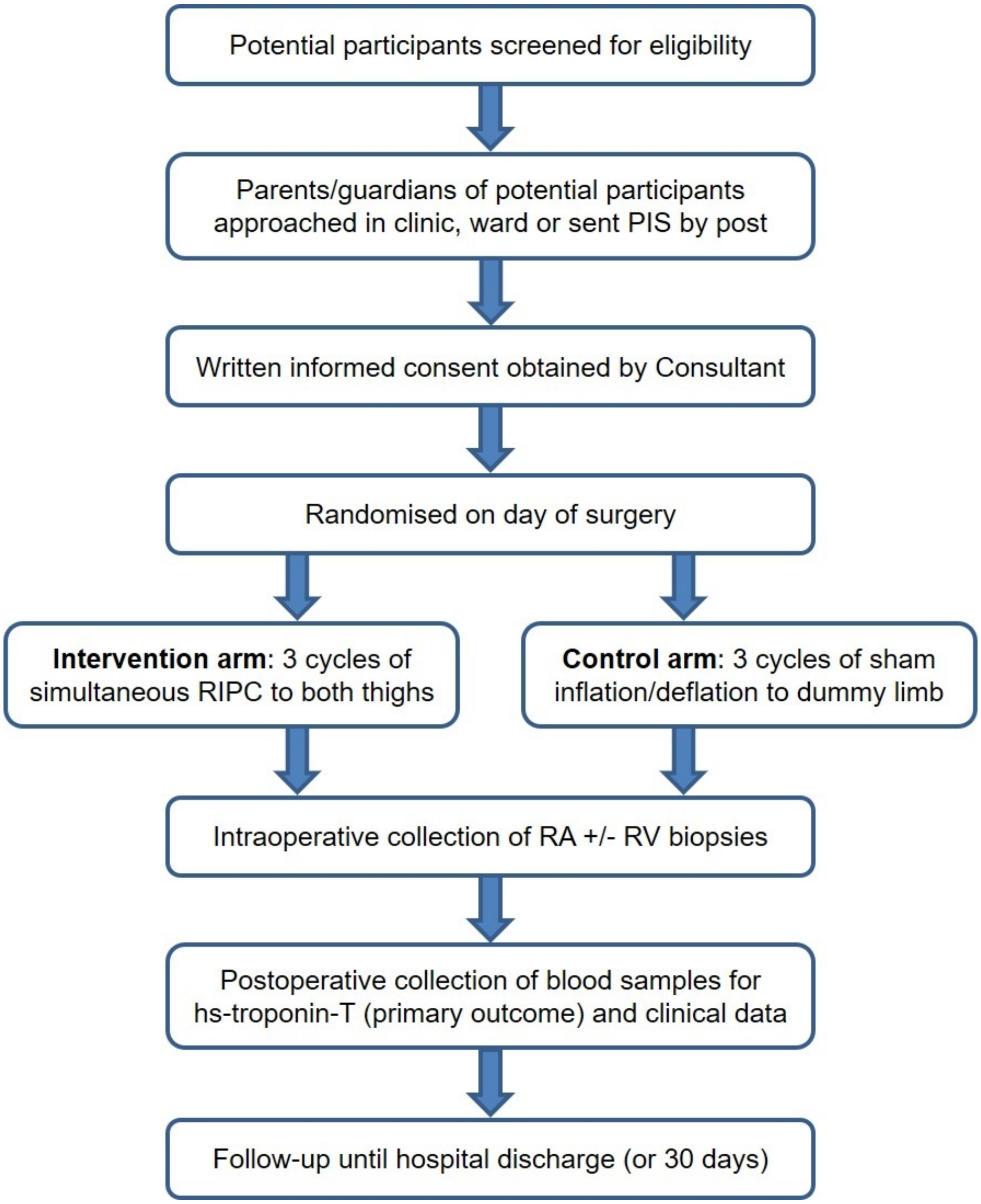

Figure 1. Participant pathway from screening to end of follow-up. PIS, parent information sheet; RA, right atrium; RIPC, remote ischaemic preconditioning; RV, right ventricle.

150x184mm (150 x 150 DPI)

British Heart Foundation

UNIVERSITY OF BIRMINGHAM | BCTU Birmingham Clinical Trials Unit

NHS Birmingham Women's and Children's NHS Foundation Trust

PARENT/GUARDIAN INFORMATION SHEET

The Bilateral Remote Ischaemic Conditioning in Children trial

Chief Investigator: Mr Nigel Drury, Consultant in Paediatric Cardiac Surgery, BCH

An invitation to participate in research: The Heart Surgery team at Birmingham Children's Hospital would like to invite your child to take part in a research study. Before you decide, you need to understand why the research is being done and what it would involve for you and your child. Please read the following information carefully and take time to decide whether or not you would like your child to take part. If there is anything that is not clear or you would like more information, please ask.

Why is my child being invited to take part?

Your child has been referred to the Heart Surgery team for an operation to repair one of two common congenital heart conditions: Tetralogy of Fallot (TOF) or a Ventricular Septal Defect (VSD). We are performing a clinical trial in young children with these conditions and have approached you to see if you would like your child to take part.

What is the aim of the study?

Children like yours are born with congenital heart disease and often need operations to correct the abnormality that they were born with to improve their survival. The surgery is complex and usually involves a period of support on a heart-lung machine (cardiopulmonary bypass) whilst the defect is repaired. The surgery puts a strain on your child's heart and may potentially cause damage (called ischaemia-reperfusion injury) when the blood supply to the heart is interrupted and restored. In this study, we wish to determine if a simple technique of inflating and deflating a blood pressure cuff immediately before the operation can reduce damage to the heart during surgery for two common conditions, to improve the outcomes of children's heart surgery.

For peer review only - http://bmjopen.bmj.com/site/about/guidelines.xhtml

What procedure is being tested in this study?

We are testing whether a simple procedure, the inflation and deflation of blood pressure cuffs on both legs immediately before heart surgery, can help to protect the heart from injury during surgery for different types of congenital heart disease. No drugs are being tested in this study, only the effects of cuff inflation.

How might inflating blood pressure cuffs on the legs help to protect the heart?

It has been shown that reducing the blood flow to the arms or legs for a short period can protect the heart, lung and kidneys from injury in adults and children undergoing different types of surgery. The temporary stoppage of blood to the limb activates a reflex known as remote ischaemic pre-conditioning (RIC). Researchers in several countries, including Canada & Australia, have shown that this may reduce the extent of heart damage in young children after surgery for congenital heart disease. We are performing this study to see whether there is a difference between children who have low oxygen levels in the blood (cyanosis) and those who do not.

Will my child undergo the blood pressure cuff treatment?

This study is a double-blind, randomised controlled trial. This means that if you agree for your child to take part, they will be allocated by a computer with a 50% chance of receiving RIC with the blood pressure cuffs and a 50% chance of not receiving RIC. All other aspects of the anaesthetic, surgery and post-operative care will be the same and *neither* you *nor* the surgical team will know whether your child has received RIC. At the end of the study, the code will be revealed to see which children were in which group. This is a standard technique for preventing those doctors and nurses involved in conducting a clinical trial from potentially influencing the results.

What will happen if I agree for my child to take part?

In addition to the standard operation and post-operative care, if you agree for your child to take part in the study, the following will occur:

- Your child's Paediatric Cardiologist and with your permission, your child's GP will be informed of their participation.
- Your child will be allocated to either the RIC group or the control group by chance.
- Once they are asleep under anaesthesia, if they are in the RIC group, a blood pressure cuff will be placed around each of their upper thighs and inflated to a

For peer review only - http://bmjopen.bmj.com/site/about/guidelines.xhtml

level approximately 50mmHg higher than their own blood pressure – this will not cause any pain. The cuffs will remain inflated for 5 minutes then deflated for 5 minutes and will be repeated two more times. If they are in the control group, the blood pressure cuffs will not be placed on their legs.

- Prior to surgery, all children have small plastic lines (tubes) inserted into their blood vessels to make measurements and take blood samples. Children in the study will have additional blood samples taken from these lines (no extra needles) over the first 24 hours after surgery to detect any injury to the heart.
- During surgery, in order to repair the defect, the heart is opened through a cut in the side of the right atrium. In children in the study, two small biopsies of the atrium will be taken from the edge of the cut by the operating surgeon. Once the heart defect is repaired, the cut will be closed in the normal way; taking the biopsies does not increase the risk of the operation in any way but will allow us to understand how RIC may improve protection of the heart.
- In children with TOF, bands of muscle inside the heart which blocks the flow of blood to the lungs are routinely removed during surgery; if your child is in the study, these bands of muscle will be kept for analysis rather than be thrown away.
- After surgery, your child will be discharged home and kept under regular follow-up in the clinic; you will not need to attend any additional clinic visits for the study.

What are the benefits?

There may not be any benefits for your child. Whilst some previous studies have shown that RIC helps to protect children's hearts from injury during surgery, we do not know whether it is beneficial to all children with all types of congenital heart disease - that is why we are conducting this study. We do not know whether being in the study will make your child's surgery safer but we are conducting it in order to understand how to improve the outcomes of children's heart surgery in the future.

Are there any risks?

Previous studies have shown that RIC is safe. There have been no complications reported related to the use of a blood pressure cuff for RIC in either children or adults undergoing *any* type of surgery. The operation itself carries a risk for your child, as will have been discussed with you by your Surgeon and Cardiologist, but being involved in this study does not increase that risk in any way.

For peer review only - http://bmjopen.bmj.com/site/about/guidelines.xhtml

How many children will be taking part in this study?

We aim to recruit up to 120 children undergoing surgery for congenital heart disease in Birmingham and Leeds to take part in this study over a 4 year period.

Does my child have to take part?

Taking part in the study is entirely voluntary – you decide. This parent information sheet gives you information about the study and we can answer any questions that you may have after reading it. Before your child's surgery, one of the research team will ask you whether you wish your child to participate in this study and if so, to sign a consent form. Your child will only be included in the study if you give your express permission. Indeed, you are free to withdraw your child at any time, without giving a reason – their surgery will proceed as planned, without any additional measurements and tests, and it will not affect the standard of care that your child receives.

What if something goes wrong?

The standard care of children undergoing heart surgery involves intensive monitoring and we do not expect the study itself to cause any problems. Complications of surgery can occur and these will be dealt with in the normal manner, regardless of the research study. Your child's safety during and after surgery is paramount. In the unlikely event that any harm should occur as a result of taking part in this study, we want you to be informed of your rights. There are no special compensation arrangements but you may have the right to claim damages in a court of law; this would require you to prove fault on the part of the NHS Trust, University or any manufacturer involved. The standard NHS complaints mechanisms are available to you; further information can be obtained from the Patient Advice & Liaison Service (PALS) at Birmingham Children's Hospital on 0121 333 8611.

What happens to my child's information and samples?

All information collected on children who participate in this study will be securely stored on Hospital and University computers. Paper copies of the data will be stored in a locked office at the Hospital. The information from the study will be analysed, presented at scientific meetings and published in medical journals to inform other doctors and health professionals of the research findings. All data will be coded and kept confidential, ensuring that your child's identity will not be revealed at any time.

For peer review only - http://bmjopen.bmj.com/site/about/guidelines.xhtml

All necessary measures will be taken to keep your child's data safe and to comply with the Data Protection Act. Following completion of the study, the data will be kept for 25 years then destroyed in accordance with national guidance. All of the blood and tissue samples collected during the study will stored in secure laboratories at the hospital, collaborating hospitals & University in accordance with Human Tissue Act. Once analysed, any remaining samples may be kept and used in future research studies which conform to all relevant legal, governance and ethical requirements.

What happens at the end of the study?

At the end of the study, your child's treatment and follow-up continues as would that of a child who had not been involved in the study.

Will I ever know if the trial worked and which treatment my child received?

Yes – but not until the whole study has finished and we have analysed the results. We will work with *Young at Heart*, a charity that offers help and support to families of babies and children diagnosed with heart defects, to produce a newsletter with the findings of the study to send to the parents of all children involved. At this stage, we will be able to tell you which group your child was in.

Who is organising and funding this research?

This study has been organised & developed by the teams at Birmingham Children's Hospital, Leeds Teaching Hospitals NHS Trust and University of Birmingham. It is funded by the **British Heart Foundation** & sponsored by University of Birmingham.

Who has reviewed this research study and leaflet?

The study has been reviewed by the British Heart Foundation and the Research & Development teams at Birmingham Children's Hospital, Leeds Teaching Hospitals NHS Trust and the University of Birmingham. It has been given a favourable opinion for conduct in the NHS by the West Midlands Solihull Research Ethics Committee. This Parent Information Sheet has been reviewed and revised by the parents of children who have had heart surgery, through the children's charity *Young at Heart*.

Questions? Contact Mr. Nigel Drury, Consultant in Paediatric Cardiac Surgery by email: xxxxx.xxxxx@nhs.net or via the BCH switchboard on xxxx xxx xxxx.

For peer review only

For peer review only - http://bmjopen.bmj.com/site/about/guidelines.xhtml

Flow chart for the Bilateral Remote Ischaemic Conditioning in Children trial

You will have been given this information sheet by your Consultant or a member of the research team in the clinic or been sent it in the post.

↓

Please read it carefully and consider whether you would like your child to take part. If you have any questions, please feel free to ask.

↓

We will contact you either at the pre-operative assessment clinic or when your child is admitted for their operation.

↓

If you are happy for your child to take part, you will be asked to sign a Consent form by a member of the research team.

↓

On the day of surgery, you may go along to theatre with your child as usual. After they have gone to sleep, the computer will allocate them to a group and they will receive *either* RIC *or* no RIC just before their operation. During surgery, the blood & tissue samples will be removed for analysis.

↓

After the operation, they will be transferred to the Paediatric Intensive Care Unit (PICU) as usual and you will be able to see them there.

↓

Whilst they are recovering on PICU, several additional blood tests will be performed using the lines already in place – no new needles required.

↓

When they are well enough, your child will be transferred to the ward and then discharged from hospital once they are ready to go home.

↓

After discharge, they will be seen regularly in the outpatient clinic but there will be no additional follow-up appointment related to the study.

↓

Once the trial has completed, we will send you a newsletter with the results.

Thank you for reading this information & considering your child's participation

For peer review only - http://bmjopen.bmj.com/site/about/guidelines.xhtml

PARENT/GUARDIAN INFORMATION SHEET

The Bilateral Remote Ischaemic Conditioning in Children trial

Principal Investigator: Ms Carin van Doorn, Consultant Paediatric Cardiac Surgeon

An invitation to participate in research: The Heart Surgery team at Leeds Children's Hospital would like to invite your child to take part in a research study. Before you decide, you need to understand why the research is being done and what it would involve for you and your child. Please read the following information carefully and take time to decide whether or not you would like your child to take part. If there is anything that is not clear or you would like more information, please ask.

Why is my child being invited to take part?

Your child has been referred to the Heart Surgery team for an operation to repair one of two common congenital heart conditions: Tetralogy of Fallot (TOF) or a Ventricular Septal Defect (VSD). We are performing a clinical trial in young children with these conditions and have approached you to see if you would like your child to take part.

What is the aim of the study?

Children like yours are born with congenital heart disease and often need operations to correct the abnormality that they were born with to improve their survival. The surgery is complex and usually involves a period of support on a heart-lung machine (cardiopulmonary bypass) whilst the defect is repaired. The surgery puts a strain on your child's heart and may potentially cause damage (called ischaemia-reperfusion injury) when the blood supply to the heart is interrupted and restored. In this study, we wish to determine if a simple technique of inflating and deflating a blood pressure cuff immediately before the operation can reduce damage to the heart during surgery for two common conditions, to improve the outcomes of children's heart surgery.

For peer review only - http://bmjopen.bmj.com/site/about/guidelines.xhtml

What procedure is being tested in this study?

We are testing whether a simple procedure, the inflation and deflation of blood pressure cuffs on both legs immediately before heart surgery, can help to protect the heart from injury during surgery for different types of congenital heart disease. No drugs are being tested in this study, only the effects of cuff inflation.

How might inflating blood pressure cuffs on the legs help to protect the heart?

It has been shown that reducing the blood flow to the arms or legs for a short period can protect the heart, lung and kidneys from injury in adults and children undergoing different types of surgery. The temporary stoppage of blood to the limb activates a reflex known as remote ischaemic pre-conditioning (RIC). Researchers in several countries, including Canada & Australia, have shown that this may reduce the extent of heart damage in young children after surgery for congenital heart disease. We are performing this study to see whether there is a difference between children who have low oxygen levels in the blood (cyanosis) and those who do not.

Will my child undergo the blood pressure cuff treatment?

This study is a double-blind, randomised controlled trial. This means that if you agree for your child to take part, they will be allocated by a computer with a 50% chance of receiving RIC with the blood pressure cuffs and a 50% chance of not receiving RIC. All other aspects of the anaesthetic, surgery and post-operative care will be the same and *neither* you *nor* the surgical team will know whether your child has received RIC. At the end of the study, the code will be revealed to see which children were in which group. This is a standard technique for preventing those doctors and nurses involved in conducting a clinical trial from potentially influencing the results.

What will happen if I agree for my child to take part?

In addition to the standard operation and post-operative care, if you agree for your child to take part in the study, the following will occur:

- Your child's Paediatric Cardiologist and with your permission, your child's GP will be informed of their participation.
- Your child will be allocated to either the RIC group or the control group by chance.
- Once they are asleep under anaesthesia, if they are in the RIC group, a blood pressure cuff will be placed around each of their upper thighs and inflated to a

For peer review only - http://bmjopen.bmj.com/site/about/guidelines.xhtml

level approximately 50mmHg higher than their own blood pressure – this will not cause any pain. The cuffs will remain inflated for 5 minutes then deflated for 5 minutes and will be repeated two more times. If they are in the control group, the blood pressure cuffs will not be placed on their legs.

- Prior to surgery, all children have small plastic lines (tubes) inserted into their blood vessels to make measurements and take blood samples. Children in the study will have additional blood samples taken from these lines (no extra needles) over the first 24 hours after surgery to detect any injury to the heart.
- After surgery, your child will be discharged home and kept under regular follow-up in the clinic; you will not need to attend any additional clinic visits for the study.

What are the benefits?

There may not be any benefits for your child. Whilst some previous studies have shown that RIC helps to protect children's hearts from injury during surgery, we do not know whether it is beneficial to all children with all types of congenital heart disease - that is why we are conducting this study. We do not know whether being in the study will make your child's surgery safer but we are conducting it in order to understand how to improve the outcomes of children's heart surgery in the future.

Are there any risks?

Previous studies have shown that RIC is safe. There have been no complications reported related to the use of a blood pressure cuff for RIC in either children or adults undergoing *any* type of surgery. The operation itself carries a risk for your child, as will have been discussed with you by your Surgeon and Cardiologist, but being involved in this study does not increase that risk in any way.

How many children will be taking part in this study?

We aim to recruit up to 120 children undergoing surgery for congenital heart disease in Birmingham and Leeds to take part in this study over a 4 year period.

Does my child have to take part?

Taking part in the study is entirely voluntary – you decide. This parent information sheet gives you information about the study and we can answer any questions that you may have after reading it. Before your child's surgery, one of the research team

For peer review only - http://bmjopen.bmj.com/site/about/guidelines.xhtml

For peer review only

will ask you whether you wish your child to participate in this study and if so, to sign a consent form. Your child will only be included in the study if you give your express permission. Indeed, you are free to withdraw your child at any time, without giving a reason – their surgery will proceed as planned, without any additional measurements and tests, and it will not affect the standard of care that your child receives.

What if something goes wrong?

The standard care of children undergoing heart surgery involves intensive monitoring and we do not expect the study itself to cause any problems. Complications of surgery can occur and these will be dealt with in the normal manner, regardless of the research study. Your child's safety during and after surgery is paramount. In the unlikely event that any harm should occur as a result of taking part in this study, we want you to be informed of your rights. There are no special compensation arrangements but you may have the right to claim damages in a court of law; this would require you to prove fault on the part of the NHS Trust, University or any manufacturer involved. The standard NHS complaints mechanisms are available to you; further information can be obtained from the Patient Advice & Liaison Service (PALS) at Leeds Teaching Hospitals on 0113 206 6261.

What happens to my child's information and samples?

All information collected on children who participate in this study will be securely stored on Hospital and University computers. Paper copies of the data will be stored in a locked office at the Hospital. The information from the study will be analysed, presented at scientific meetings and published in medical journals to inform other doctors and health professionals of the research findings. All data will be coded and kept confidential, ensuring that your child's identity will not be revealed at any time. All necessary measures will be taken to keep your child's data safe and to comply with the Data Protection Act. Following completion of the study, the data will be kept for 25 years then destroyed in accordance with national guidance. All of the blood and tissue samples collected during the study will stored in secure laboratories at the hospital, collaborating hospitals & University in accordance with Human Tissue Act. Once analysed, any remaining samples may be kept and used in future research studies which conform to all relevant legal, governance and ethical requirements.

For peer review only - http://bmjopen.bmj.com/site/about/guidelines.xhtml

What happens at the end of the study?

At the end of the study, your child's treatment and follow-up continues as would that of a child who had not been involved in the study.

Will I ever know if the trial worked and which treatment my child received?

Yes – but not until the whole study has finished and we have analysed the results. We will work with *Young at Heart*, a charity that offers help and support to families of babies and children diagnosed with heart defects, to produce a newsletter with the findings of the study to send to the parents of all children involved. At this stage, we will be able to tell you which group your child was in.

Who is organising and funding this research?

This study has been organised & developed by the teams at Birmingham Children's Hospital, Leeds Teaching Hospitals NHS Trust and University of Birmingham. It is funded by the **British Heart Foundation** & sponsored by University of Birmingham.

Who has reviewed this research study and leaflet?

The study has been reviewed by the British Heart Foundation and the Research & Development teams at Birmingham Children's Hospital, Leeds Teaching Hospitals NHS Trust and the University of Birmingham. It has been given a favourable opinion for conduct in the NHS by the West Midlands Solihull Research Ethics Committee. This Parent Information Sheet has been reviewed and revised by the parents of children who have had heart surgery, through the children's charity *Young at Heart*.

Questions? Contact Ms Carin van Doorn, Clinical Lead & Consultant Congenital Cardiac Surgeon at Leeds Teaching Hospitals by phone: xxxxx xxxxxx, or Collette Spencer, Research Nurse by email: xxxxxxx@nhs.net or phone: xxxx xxx xxxx.

For peer review only - http://bmjopen.bmj.com/site/about/guidelines.xhtml

Flow chart for the Bilateral Remote Ischaemic Conditioning in Children trial

You will have been given this information sheet by your Consultant or a member of the research team in the clinic or been sent it in the post.

↓

Please read it carefully and consider whether you would like your child to take part. If you have any questions, please feel free to ask.

↓

We will contact you either at the pre-operative assessment clinic or when your child is admitted for their operation.

↓

If you are happy for your child to take part, you will be asked to sign a Consent form by a member of the research team.

↓

On the day of surgery, you may go along to theatre with your child as usual. After they have gone to sleep, the computer will allocate them to a group and they will receive *either* RIC *or* no RIC just before their operation. During surgery, a blood sample will be obtained for analysis.

↓

After the operation, they will be transferred to the Paediatric Intensive Care Unit (PICU) as usual and you will be able to see them there.

↓

Whilst they are recovering on PICU, several additional blood tests will be performed using the lines already in place – no new needles required.

↓

When they are well enough, your child will be transferred to the ward and then discharged from hospital once they are ready to go home.

↓

After discharge, they will be seen regularly in the outpatient clinic but there will be no additional follow-up appointment related to the study.

↓

Once the trial has completed, we will send you a newsletter with the results.

Thank you for reading this information & considering your child's participation

For peer review only

For peer review only - http://bmjopen.bmj.com/site/about/guidelines.xhtml

| 1 | | | |

BRICC trial – ISRCTN12923441 IRAS: 200876 Version 1.9a: 22/11/2019

PARENT/GUARDIAN CONSENT FORM

The Bilateral Remote Ischaemic Conditioning in Children trial

Chief Investigator: Mr Nigel Drury, Consultant in Paediatric Cardiac Surgery, BCH

Please initial boxes

I confirm that I have read and understand the parent/guardian information sheet (version 1.9a, dated 22/11/2019) for the above study. I have had the opportunity to consider the study information, ask questions and have had these answered satisfactorily. ☐

I understand that my child's participation is voluntary and that I am free to withdraw at any time, without giving a reason and without my child's care or legal rights being affected. ☐

I understand that relevant sections of any of my child's medical notes and data collected during the study may be looked at by responsible individuals from the NHS Trusts, the University of Birmingham or the regulatory authorities, where it is relevant to my taking part in this research. I give permission for these individuals to have access to my child's records. ☐

I consent to the storage, including electronic, of personal information for the purposes of this study. I understand that any information that could identify me or my child will be kept strictly confidential and that no personal information will be included in the study report or other publication. ☐

I understand that blood and tissue samples will be kept for the purposes of research and I give permission for these samples to be taken and stored. ☐

I understand that any remaining samples may be stored beyond the end of this trial and used in future research which conforms to all relevant legal, governance and ethical requirements. ☐

I agree to my child's GP and/or other doctors involved in their care, being informed of my child's participation in the study. ☐

Name of Child: Date of birth:

Name of Parent/Guardian	Signature of Parent/Guardian	Date signed
Name of Investigator	Signature of Investigator	Date signed

Once completed: 1 for parent(s), 1 for medical notes and 1 (original) for trial site file

For peer review only - http://bmjopen.bmj.com/site/about/guidelines.xhtml

1 | | | | BRICC trial – ISRCTN12923441 IRAS: 200876 Version 1.9b: 22/11/2019

PARENT/GUARDIAN CONSENT FORM

The Bilateral Remote Ischaemic Conditioning in Children trial

Principal Investigator: Ms Carin van Doorn, Consultant Paediatric Cardiac Surgeon

Please initial boxes

I confirm that I have read and understand the parent/guardian information sheet (version 1.9b, dated 22/11/2019) for the above study. I have had the opportunity to consider the study information, ask questions and have had these answered satisfactorily. ☐

I understand that my child's participation is voluntary and that I am free to withdraw at any time, without giving a reason and without my child's care or legal rights being affected. ☐

I understand that relevant sections of any of my child's medical notes and data collected during the study may be looked at by responsible individuals from the NHS Trusts, the University of Birmingham or the regulatory authorities, where it is relevant to my taking part in this research. I give permission for these individuals to have access to my child's records. ☐

I consent to the storage, including electronic, of personal information for the purposes of this study. I understand that any information that could identify me or my child will be kept strictly confidential and that no personal information will be included in the study report or other publication. ☐

I understand that blood samples will be kept for the purposes of research and I give permission for these samples to be taken and stored. ☐

I understand that any remaining samples may be stored beyond the end of this trial and used in future research which conforms to all relevant legal, governance and ethical requirements. ☐

I agree to my child's GP and/or other doctors involved in their care, being informed of my child's participation in the study. ☐

Name of Child: Date of birth:

Name of Parent/Guardian	Signature of Parent/Guardian	Date signed
Name of Investigator	Signature of Investigator	Date signed

Once completed: 1 for parent(s), 1 for medical notes and 1 (original) for trial site file

For peer review only

For peer review only - http://bmjopen.bmj.com/site/about/guidelines.xhtml

Data Monitoring Committee (DMC) Charter

The Bilateral Remote Ischaemic Conditioning in Children trial

For peer review only

1. INTRODUCTION

Title: The Bilateral Remote Ischaemic Conditioning in Children (BRICC) trial: a two-centre, double-blind, randomised controlled trial of remote ischaemic preconditioning in normoxic and hypoxic children undergoing cardiac surgery – ISRCTN12923441.

Chief Investigator: Mr Nigel Drury, Consultant in Paediatric Cardiac Surgery, BCH

TMC Chair: Mr Timothy Jones, Consultant Paediatric Cardiac Surgeon, BCH

IRAS: 200876, protocol: v1.6, 05/02/18, approved by REC: 28/02/18, HRA: 28/02/18.

Sponsor: University of Birmingham (RG_14-025), in partnership with Birmingham Women's and Children's NHS Foundation Trust (1845) and Leeds Teaching Hospitals NHS Trust (PA17/67348).

Funder: British Heart Foundation (FS/15/49/31612)

Objectives: The BRICC trial is a two-centre, double-blind, randomised controlled trial to assess whether adequately delivered remote ischaemic preconditioning is cardioprotective in young children undergoing surgical repair of two common congenital heart defects, through clinical and biochemical endpoints. It will also evaluate the effects of preconditioning in the presence or absence of chronic preoperative hypoxia by evaluating the myocardial metabolic phenotype.

Scope: The purpose of this document is to describe the roles and responsibilities of the independent DMC for the BRICC trial, including the terms of reference, the frequency and format of meetings, methods of providing information to and from the DMC, statistical issues and relationship with the Trial Management Committee.

2. ROLES & RESPONSIBILITIES

Aims: To safeguard the interests of trial participants & their families, assess the safety and efficacy of the interventions during the trial and monitor the overall conduct of the clinical trial.

Terms of reference: The DMC should receive and review the progress and accruing data from the trial and make recommendations on the conduct of the trial to the Trial Management Committee. Meetings will be scheduled approximately every 8 months and key outputs will be made available for review; changes to this schedule may be made by the DMC as required. Review(s) will include updated figures on recruitment, data quality, efficacy outcomes and safety data. Specifically, the DMC will:

- Assess data quality, including completeness
- Monitor recruitment and losses to follow-up
- Monitor compliance with the protocol by participants and investigators
- Monitor evidence for treatment differences in the main efficacy outcomes
- Monitor evidence of treatment harm (limb complications, SAEs)
- Decide whether to recommend that the trial continues or whether recruitment should be terminated for some or all of the treatment groups
- Suggest additional data analyses
- Advise on protocol modifications suggested by the Investigators
- Assess the impact and relevance of external evidence
- Monitor compliance with previous DMC recommendations

Furthermore, members of the DMC will not disclose interim results or use them for financial gain, nor share blinded data with anyone outside of the DMC, including the Chief Investigator. All DMC and TMC members will sign a Conflict of Interest form.

3. BEFORE OR EARLY IN THE TRIAL

Members of the DMC were invited to participate in the committee in the spring of 2016 and therefore were not involved in development of the trial protocol. The DMC were asked to meet before or early in the trial (within 3 months of commencing recruitment) to review this Charter, revise as required and approve the working version; this initial meeting took place on 18th November 2016.

For peer review only - http://bmjopen.bmj.com/site/about/guidelines.xhtml

4. COMPOSITION

The members of the independent DMC for the BRICC trial are:

- Prof Gavin Murphy, BHF Chair of Cardiac Surgery, University of Leicester (Chair)
- Dr Katherine Brown, Consultant in Cardiac Intensive Care, GOSH, London
- Dr Peter Nightingale, Statistician, Queen Elizabeth Hospital Birmingham

At their initial meeting, the members of the DMC nominated Prof Murphy as Chair with responsibility for facilitating discussion and communicating review outcomes.

5. RELATIONSHIPS

In addition to the independent DMC, the conduct of the trial is overseen by members of the Trial Management Committee, who have developed and approved the trial protocol and have executive responsibility for the conduct of the trial; there is no Trial Steering Committee. The DMC has an advisory role and will make recommendations regarding efficacy and the safety of participants to the Trial Management Committee.

6. ORGANISATION

The DMC discussed the first version of this Charter and made recommendations based on best practice which have been implemented. During the trial, they will meet every 8 months to review efficacy and safety data. Analysis of hs-troponin-T for the primary outcome will be performed prior to each meeting and the unblinded data made available. Meetings may be held via teleconference, without the need to meet face-to-face unless deemed necessary, and will employ the following format:

1. *Open session*: Introduction, presentation of progress report
2. *Closed session*: Discussion of closed parts of the report
3. *Open session*: Discussion of any matters arising from previous sessions
4. *Closed session*: Further discussion, if required.

Closed sessions will be attended only by members of the DMC and others whom are specifically invited as discussion will include unblinded efficacy and safety data by treatment group. In open sessions, they will be joined by the Chief Investigator, Trial Statistician and other interested parties which may include Research Nurse(s) and representatives of the R&D Office, sponsor, funder or regulator, as required.

For peer review only - http://bmjopen.bmj.com/site/about/guidelines.xhtml

7. DOCUMENTATION

The Chief Investigator and Trial Statistician will produce a progress report for each DMC review, which should be received by members at least 2 weeks beforehand, documenting the key outputs of the trial including graphs showing expected and observed recruitment, CONSORT diagrams, descriptions of the trial cohort, protocol compliance, procedural and outcome measures, and adverse events reported by group. Specifically it will include:

- *Primary efficacy outcome*: area under the time-concentration curve for hs-troponin-T release in the first 24 hours after aortic cross-clamp release, calculated by the trapezoid rule from samples taken at baseline, 3, 6, 12 and 24 hours.
- *Primary safety outcome*: frequency of expected Serious Adverse Events (SAEs) reported to the Sponsor: death; requirement for extracorporeal life support (ECLS); evidence of a major neurological event; or the need for further surgery in the early post-operative period, such as for bleeding or a residual VSD.
- *Secondary efficacy outcomes*: peak hs-troponin-T in the first 12 hours; vasoactive inotrope score in the first 12 hours; arterial lactate and central venous oxygen saturations in first 12 hours; and lengths of stay in the paediatric intensive care unit and the hospital. Cardiac index in the first 12 hours is an exploratory outcome.

DMC members should store their papers and reports safely after each meeting so that they can cross-check with later reports. After the trial is finally reported, all interim reports should be destroyed.

8. DECISION MAKING

The DMC should consider the following possible recommendations following interim analysis of the conduct of the trial and the safety and efficacy data:

1. No action needed, trial continues as planned.
2. Early stopping due to clear treatment benefit or harm, futility or external evidence.
3. Stopping recruitment within a subgroup.
4. Extending recruitment (based on actual control arm response rates being different to predicted rather than on emerging differences) or extending follow-up.
5. Sanctioning and/or proposing protocol changes.

For peer review only - http://bmjopen.bmj.com/site/about/guidelines.xhtml

The DMC agreed that no firm statistical criteria (often called *stopping rules*) would be defined, rather DMC recommendations would be based on the ability of the trial to test its primary hypothesis and the overall interpretation of the safety data and secondary outcomes that include important safety endpoints. The DMC will consider the balance of primary risks and benefits, the internal consistency of results, the consistence with and nature of external evidence, and the likelihood that results would affect clinical practice. Decisions should be achieved by consensus and be unanimous when possible, using both informal and formal decision-making strategies, as required.

If the DMC recommends stopping recruitment, an urgent face-to-face meeting should be arranged between the DMC Chair, Chief Investigator, key members of the Trial Management Committee and the Sponsor to reach a decision on continuing the trial.

9. REPORTING

The DMC will report its recommendations in writing to the Trial Management Committee and Sponsor. A formal record should also be made of both closed and open sessions, documenting the major points of discussion, any decisions and actions and their reasons, and any additional information needed for future meetings; however, names do not need to be attributed to all comments.

10. AFTER THE TRIAL

Responsibility for reporting the findings of the trial lies with the Chief Investigator and Trial Management Committee, although the DMC should encourage timely reporting. The DMC Chair should approve the accuracy of trial manuscripts submitted for publication in which all members of the DMC will be acknowledged.

REFERENCES

- DAMOCLES Study Group. A proposed charter for clinical trial data monitoring committees: helping them to do their job well. *Lancet* 2005; 365: 711-22.
- National Research Ethics Service. Data Monitoring Committees in Clinical Trials. Guidance for Research Ethics Committees. NPSA, May 2010

For peer review only - http://bmjopen.bmj.com/site/about/guidelines.xhtml

STANDARD PROTOCOL ITEMS: RECOMMENDATIONS FOR INTERVENTIONAL TRIALS

SPIRIT 2013 Checklist: Recommended items to address in a clinical trial protocol and related documents*

Section/item	Item No	Description	Addressed on page number
**Administrative information**			
Title	1	Descriptive title identifying the study design, population, interventions, and, if applicable, trial acronym	____1______
Trial registration	2a	Trial identifier and registry name. If not yet registered, name of intended registry	____2______
	2b	All items from the World Health Organization Trial Registration Data Set	__ISRCTN__
Protocol version	3	Date and version identifier	____22_____
Funding	4	Sources and types of financial, material, and other support	____21_____
Roles and responsibilities	5a	Names, affiliations, and roles of protocol contributors	____1,21___
	5b	Name and contact information for the trial sponsor	____18_____
	5c	Role of study sponsor and funders, if any, in study design; collection, management, analysis, and interpretation of data; writing of the report; and the decision to submit the report for publication, including whether they will have ultimate authority over any of these activities	____21-22___
	5d	Composition, roles, and responsibilities of the coordinating centre, steering committee, endpoint adjudication committee, data management team, and other individuals or groups overseeing the trial, if applicable (see Item 21a for data monitoring committee)	____15_____

For peer review only

Introduction

Background and rationale	6a	Description of research question and justification for undertaking the trial, including summary of relevant studies (published and unpublished) examining benefits and harms for each intervention	____4-6____
	6b	Explanation for choice of comparators	____6______
Objectives	7	Specific objectives or hypotheses	____6______
Trial design	8	Description of trial design including type of trial (eg, parallel group, crossover, factorial, single group), allocation ratio, and framework (eg, superiority, equivalence, noninferiority, exploratory)	____7-8____

Methods: Participants, interventions, and outcomes

Study setting	9	Description of study settings (eg, community clinic, academic hospital) and list of countries where data will be collected. Reference to where list of study sites can be obtained	____7______
Eligibility criteria	10	Inclusion and exclusion criteria for participants. If applicable, eligibility criteria for study centres and individuals who will perform the interventions (eg, surgeons, psychotherapists)	____7______
Interventions	11a	Interventions for each group with sufficient detail to allow replication, including how and when they will be administered	____9-10____
	11b	Criteria for discontinuing or modifying allocated interventions for a given trial participant (eg, drug dose change in response to harms, participant request, or improving/worsening disease)	____9______
	11c	Strategies to improve adherence to intervention protocols, and any procedures for monitoring adherence (eg, drug tablet return, laboratory tests)	____10_____
	11d	Relevant concomitant care and interventions that are permitted or prohibited during the trial	____10-11___
Outcomes	12	Primary, secondary, and other outcomes, including the specific measurement variable (eg, systolic blood pressure), analysis metric (eg, change from baseline, final value, time to event), method of aggregation (eg, median, proportion), and time point for each outcome. Explanation of the clinical relevance of chosen efficacy and harm outcomes is strongly recommended	____12-13___
Participant timeline	13	Time schedule of enrolment, interventions (including any run-ins and washouts), assessments, and visits for participants. A schematic diagram is highly recommended (see Figure)	___Figure 1___

For peer review only - http://bmjopen.bmj.com/site/about/guidelines.xhtml

Sample size	14	Estimated number of participants needed to achieve study objectives and how it was determined, including clinical and statistical assumptions supporting any sample size calculations	____13____
Recruitment	15	Strategies for achieving adequate participant enrolment to reach target sample size	____13-15__

Methods: Assignment of interventions (for controlled trials)

Allocation:

Sequence generation	16a	Method of generating the allocation sequence (eg, computer-generated random numbers), and list of any factors for stratification. To reduce predictability of a random sequence, details of any planned restriction (eg, blocking) should be provided in a separate document that is unavailable to those who enrol participants or assign interventions	____8-9____
Allocation concealment mechanism	16b	Mechanism of implementing the allocation sequence (eg, central telephone; sequentially numbered, opaque, sealed envelopes), describing any steps to conceal the sequence until interventions are assigned	____8-9____
Implementation	16c	Who will generate the allocation sequence, who will enrol participants, and who will assign participants to interventions	____8-9____
Blinding (masking)	17a	Who will be blinded after assignment to interventions (eg, trial participants, care providers, outcome assessors, data analysts), and how	____9______
	17b	If blinded, circumstances under which unblinding is permissible, and procedure for revealing a participant's allocated intervention during the trial	____N/A____

Methods: Data collection, management, and analysis

Data collection methods	18a	Plans for assessment and collection of outcome, baseline, and other trial data, including any related processes to promote data quality (eg, duplicate measurements, training of assessors) and a description of study instruments (eg, questionnaires, laboratory tests) along with their reliability and validity, if known. Reference to where data collection forms can be found, if not in the protocol	____11-13___
	18b	Plans to promote participant retention and complete follow-up, including list of any outcome data to be collected for participants who discontinue or deviate from intervention protocols	____N/A____

Data management	19	Plans for data entry, coding, security, and storage, including any related processes to promote data quality (eg, double data entry; range checks for data values). Reference to where details of data management procedures can be found, if not in the protocol	____16_____
Statistical methods	20a	Statistical methods for analysing primary and secondary outcomes. Reference to where other details of the statistical analysis plan can be found, if not in the protocol	____14_____
	20b	Methods for any additional analyses (eg, subgroup and adjusted analyses)	____14_____
	20c	Definition of analysis population relating to protocol non-adherence (eg, as randomised analysis), and any statistical methods to handle missing data (eg, multiple imputation)	____14_____
**Methods: Monitoring**			
Data monitoring	21a	Composition of data monitoring committee (DMC); summary of its role and reporting structure; statement of whether it is independent from the sponsor and competing interests; and reference to where further details about its charter can be found, if not in the protocol. Alternatively, an explanation of why a DMC is not needed	__15, App E__
	21b	Description of any interim analyses and stopping guidelines, including who will have access to these interim results and make the final decision to terminate the trial	____App E__
Harms	22	Plans for collecting, assessing, reporting, and managing solicited and spontaneously reported adverse events and other unintended effects of trial interventions or trial conduct	____14-15__
Auditing	23	Frequency and procedures for auditing trial conduct, if any, and whether the process will be independent from investigators and the sponsor	____16_____
**Ethics and dissemination**			
Research ethics approval	24	Plans for seeking research ethics committee/institutional review board (REC/IRB) approval	____18_____
Protocol amendments	25	Plans for communicating important protocol modifications (eg, changes to eligibility criteria, outcomes, analyses) to relevant parties (eg, investigators, REC/IRBs, trial participants, trial registries, journals, regulators)	____18_____

Consent or assent	26a	Who will obtain informed consent or assent from potential trial participants or authorised surrogates, and how (see Item 32)	____8______
	26b	Additional consent provisions for collection and use of participant data and biological specimens in ancillary studies, if applicable	____16_____
Confidentiality	27	How personal information about potential and enrolled participants will be collected, shared, and maintained in order to protect confidentiality before, during, and after the trial	____15-16___
Declaration of interests	28	Financial and other competing interests for principal investigators for the overall trial and each study site	____22_____
Access to data	29	Statement of who will have access to the final trial dataset, and disclosure of contractual agreements that limit such access for investigators	____14_____
Ancillary and post-trial care	30	Provisions, if any, for ancillary and post-trial care, and for compensation to those who suffer harm from trial participation	___App A/B__
Dissemination policy	31a	Plans for investigators and sponsor to communicate trial results to participants, healthcare professionals, the public, and other relevant groups (eg, via publication, reporting in results databases, or other data sharing arrangements), including any publication restrictions	____19_____
	31b	Authorship eligibility guidelines and any intended use of professional writers	____19_____
	31c	Plans, if any, for granting public access to the full protocol, participant-level dataset, and statistical code	____19_____
**Appendices**			
Informed consent materials	32	Model consent form and other related documentation given to participants and authorised surrogates	_Appendices_
Biological specimens	33	Plans for collection, laboratory evaluation, and storage of biological specimens for genetic or molecular analysis in the current trial and for future use in ancillary studies, if applicable	__12,15-16__

*It is strongly recommended that this checklist be read in conjunction with the SPIRIT 2013 Explanation & Elaboration for important clarification on the items. Amendments to the protocol should be tracked and dated. The SPIRIT checklist is copyrighted by the SPIRIT Group under the Creative Commons "Attribution-NonCommercial-NoDerivs 3.0 Unported" license.

For peer review only - http://bmjopen.bmj.com/site/about/guidelines.xhtml

BMJ Open

The Bilateral Remote Ischaemic Conditioning in Children (BRICC) trial: protocol for a two-centre, double-blind, randomised controlled trial in young children undergoing cardiac surgery

Journal:	*BMJ Open*
Manuscript ID	bmjopen-2020-042176.R1
Article Type:	Protocol
Date Submitted by the Author:	12-Aug-2020
Complete List of Authors:	Drury, Nigel ; Birmingham Children's Hospital, Paediatric Cardiac Surgery; University of Birmingham, Institute of Cardiovascular Sciences<br>Bi, Rehana; Birmingham Children's Hospital, Paediatric Cardiac Surgery; Birmingham Children's Hospital, Paediatric Intensive Care<br>Woolley, Rebecca; University of Birmingham, Birmingham Clinical Trials Unit; University of Birmingham, Institute of Applied Health Research<br>Stickley, John; Birmingham Children's Hospital, Paediatric Cardiac Surgery<br>Morris, Kevin; Birmingham Children's Hospital, Paediatric Intensive Care; University of Birmingham, Institute of Applied Health Research<br>Montgomerie, James; Birmingham Children's Hospital, Paediatric Cardiac Anaesthesia<br>van Doorn, Carin; Leeds Teaching Hospitals NHS Trust, Congenital Cardiac Surgery<br>Dunn, Warwick; University of Birmingham, School of Biosciences; University of Birmingham, Phenome Centre Birmingham<br>Madhani, Melanie; University of Birmingham, Institute of Cardiovascular Sciences<br>Ives, Natalie; University of Birmingham, Birmingham Clinical Trials Unit; University of Birmingham, Institute of Applied Health Research<br>Kirchhof, Paulus; University of Birmingham, Institute of Cardiovascular Sciences; UKE, Cardiology, University Heart and Vascular Center<br>Jones, Timothy; Birmingham Children's Hospital, Paediatric Cardiac Surgery; University of Birmingham, Institute of Cardiovascular Sciences
**Primary Subject Heading**:	Cardiovascular medicine
Secondary Subject Heading:	Paediatrics, Surgery
Keywords:	Congenital heart disease < CARDIOLOGY, Paediatric cardiac surgery < PAEDIATRIC SURGERY, Clinical trials < THERAPEUTICS

SCHOLARONE™
Manuscripts

I, the Submitting Author has the right to grant and does grant on behalf of all authors of the Work (as defined in the below author licence), an exclusive licence and/or a non-exclusive licence for contributions from authors who are: i) UK Crown employees; ii) where BMJ has agreed a CC-BY licence shall apply, and/or iii) in accordance with the terms applicable for US Federal Government officers or employees acting as part of their official duties; on a worldwide, perpetual, irrevocable, royalty-free basis to BMJ Publishing Group Ltd ("BMJ") its licensees and where the relevant Journal is co-owned by BMJ to the co-owners of the Journal, to publish the Work in this journal and any other BMJ products and to exploit all rights, as set out in our licence.

The Submitting Author accepts and understands that any supply made under these terms is made by BMJ to the Submitting Author unless you are acting as an employee on behalf of your employer or a postgraduate student of an affiliated institution which is paying any applicable article publishing charge ("APC") for Open Access articles. Where the Submitting Author wishes to make the Work available on an Open Access basis (and intends to pay the relevant APC), the terms of reuse of such Open Access shall be governed by a Creative Commons licence – details of these licences and which Creative Commons licence will apply to this Work are set out in our licence referred to above.

Other than as permitted in any relevant BMJ Author's Self Archiving Policies, I confirm this Work has not been accepted for publication elsewhere, is not being considered for publication elsewhere and does not duplicate material already published. I confirm all authors consent to publication of this Work and authorise the granting of this licence.

For peer review only

The Bilateral Remote Ischaemic Conditioning in Children (BRICC) trial: protocol for a two-centre, double-blind, randomised controlled trial in young children undergoing cardiac surgery

Nigel E Drury [1,2], Rehana Bi [1,3], Rebecca L Woolley [4,5], John Stickley [1], Kevin P Morris [3,5], James Montgomerie [6], Carin van Doorn [7], Warwick B Dunn [8,9], Melanie Madhani [2], Natalie J Ives [4,5], Paulus Kirchhof [2,10], Timothy J Jones [1,2]

[1] Department of Paediatric Cardiac Surgery, Birmingham Children's Hospital, Birmingham, UK; [2] Institute of Cardiovascular Sciences, University of Birmingham, Birmingham, UK; [3] Department of Paediatric Intensive Care, Birmingham Children's Hospital, Birmingham, UK; [4] Birmingham Clinical Trials Unit, University of Birmingham, Birmingham, UK; [5] Institute of Applied Health Research, University of Birmingham, Birmingham, UK; [6] Department of Paediatric Cardiac Anaesthesia, Birmingham Children's Hospital, Birmingham, UK; [7] Department of Congenital Cardiac Surgery, Leeds Teaching Hospitals NHS Trust, Leeds, UK; [8] School of Biosciences, University of Birmingham, Birmingham, UK; [9] Phenome Centre Birmingham, University of Birmingham, Birmingham, UK; [10] Department of Cardiology, University Heart and Vascular Center, UKE Hamburg, Hamburg, Germany.

Correspondence: Mr Nigel Drury, Department of Paediatric Cardiac Surgery, Birmingham Children's Hospital, Steelhouse Lane, Birmingham, B4 6NH, UK.

tel: +44 121 333 8731, fax: +44 121 333 9441, email: n.e.drury@bham.ac.uk

Word count: 4649 (excluding title page, abstract, references, figure, and table)

Number of figures: 1

Number of tables: 1

Keywords: Remote ischaemic preconditioning, paediatric cardiac surgery, clinical trial, tetralogy of Fallot, ventricular septal defect, cyanosis

For peer review only

ABSTRACT

Introduction: Myocardial protection against ischaemic-reperfusion injury is a key determinant of heart function and outcome following cardiac surgery in children. However, with current strategies, myocardial injury occurs routinely following aortic cross-clamping, as demonstrated by the ubiquitous rise in circulating troponin. Remote ischaemic preconditioning, the application of brief, non-lethal cycles of ischaemia and reperfusion to a distant organ or tissue, is a simple, low-risk and readily available technique which may improve myocardial protection. The Bilateral Remote Ischaemic Conditioning in Children (BRICC) trial will assess whether remote ischaemic preconditioning, applied to both lower limbs immediately prior to surgery, reduces myocardial injury in cyanotic and acyanotic young children.

Methods and analysis: The BRICC trial is a two-centre, double-blind, randomised controlled trial recruiting up to 120 young children (age 3 months to 3 years) undergoing primary repair of tetralogy of Fallot or surgical closure of an isolated ventricular septal defect. Participants will be randomised in a 1:1 ratio to either bilateral remote ischaemic preconditioning (3 x 5-minute cycles) or sham immediately prior to surgery, with follow-up until discharge from hospital or 30 days, whichever is sooner. The primary outcome is reduction in area under the time-concentration curve for high-sensitivity troponin-T release in the first 24 hours after aortic cross-clamp release. Secondary outcome measures include peak hs-troponin-T, vasoactive inotrope score, arterial lactate, and central venous oxygen saturations in the first 12 hours, and lengths of stay in the paediatric intensive care unit and the hospital.

Ethics and dissemination: The trial was approved by the West Midlands-Solihull NHS Research Ethics Committee (16/WM/0309) on 5 August 2016. Findings will be disseminated to the academic community through peer-reviewed publications and presentation at national & international meetings. Parents will be informed of the results through a newsletter in conjunction with a local charity.

For peer review only

For peer review only - http://bmjopen.bmj.com/site/about/guidelines.xhtml

Trial registration: ISRCTN12923441, registered May 2016.

Strengths and limitations of this study

- This is the first randomised controlled trial to evaluate the efficacy of bilateral remote ischaemic preconditioning, applied simultaneously to both lower limbs to provide a more intense stimulus in young patients undergoing surgery.
- It is also the first multi-centre cardiac surgical trial in children in the UK.
- We will exclude neonates, in whom preconditioning may be harmful, and avoid the use of propofol anaesthesia, which is thought to interfere with the preconditioning pathway.
- A potential limitation is if exposure to cyanosis in those with tetralogy of Fallot has already had a preconditioning effect, this could attenuate the effect of the intervention.
- The effect of the intervention may also be concealed if right ventricular incision, muscle resection, or outflow tract stent removal significantly increase troponin release in patients with tetralogy of Fallot above that associated with ischaemia-reperfusion.

For peer review only

INTRODUCTION

Myocardial protection

During most surgery for congenital heart disease, it is necessary to stop the heart, allowing access to a still and bloodless field to enable repair of intracardiac defects. Cardioplegia and hypothermia have been fundamental to arresting the heart and protecting against ischaemia-reperfusion (IR) injury during surgery for over 40 years and are used in approx. 3,500 cardiac surgical operations in children in the UK & Ireland each year [1]. However, the developing myocardium exhibits marked differences in metabolism from the adult heart [2] and as current techniques for cardioprotection were developed in adults, they may not be optimal for young children [3,4]. Myocardial injury still occurs routinely following aortic cross-clamping in children [2,5], with IR leading to a degree of contractile impairment which may manifest as low cardiac output and require inotropic support in the early postoperative period. This is a major cause of morbidity and death in the early postoperative period [6,7] and children with preoperative cyanosis are more vulnerable to the effects of IR than acyanotic children [8,9]. Postoperative elevation of circulating troponin is a biomarker of myocardial injury and has been shown to strongly correlate with clinical outcomes including level of inotropic support, duration of ventilation, ventricular dysfunction and early death [5,10]; consequently, it is the most common primary outcome measure in clinical trials of cardioprotection in children [11]. Myocardial protection therefore is a key determinant of heart function and outcome following cardiac surgery.

Remote Ischaemic Preconditioning

Remote ischaemic preconditioning (RIPC) involves the application of brief, non-lethal cycles of ischaemia and reperfusion to a distant organ or tissue, such as a limb, to induce protection against subsequent myocardial IR injury [12]. There are thought to be two phases of cardioprotection: a first window with an immediate effect lasting several hours, and a

For peer review only - http://bmjopen.bmj.com/site/about/guidelines.xhtml

second window which appears around 12-24 hours and lasts for 48-72 hours [13]. The stimulus has traditionally been applied to the upper arm (adults) or thigh (children) using a blood pressure cuff inflated to above systolic pressure [14]. The promise of this simple, low-risk, inexpensive and readily available technique as an adjunct to current methods for myocardial protection has prompted numerous trials in adults [15-20] and children [21-28] but with mixed results. A meta-analysis suggested that RIPC reduces myocardial injury in both adult and paediatric cardiac surgery [29], but subsequently two large multi-centre trials in adults failed to show benefit in either composite cardiovascular endpoints or troponin release [19,20]; both have been criticised for using propofol anaesthesia after it had been suggested to interfere with the preconditioning pathway [30,31].

Cheung et al first demonstrated reductions in troponin release and perioperative inotropic requirements in a heterogeneous cohort of children, most of whom had either tetralogy of Fallot or ventricular septal defect (VSD) [21]. Several studies have found improved myocardial protection in infants and young children undergoing tetralogy of Fallot repair [28] or VSD closure [22,23], whilst others have found no benefit [24,25] and suggested that preoperative cyanosis may have already up-regulated pro-survival pathways [25]. The only trial in cyanosed neonates found no benefit, citing young age, myocardial immaturity and chronic hypoxaemia as potential conflicting factors [26]; animal models have also suggested that preconditioning may have no effect [32] or even be harmful [33] to the immature myocardium. To date, no clinical trials have compared the effects of RIPC in patients with or without chronic cyanosis and its impact on preconditioning remains uncertain [34].

In the largest paediatric trial to date, McCrindle et al. found no benefit in clinical outcomes, physiological markers or subgroup analyses in a mixed cohort of 299 children [27] and proposed that better than expected outcomes in the control group, heterogeneity of underlying conditions, and use of propofol may have affected their findings. Failure to elicit a stimulus may also have been a key factor; manual inflation of the cuff to just 15mmHg above systolic pressure may have led to periods of subclinical reperfusion and abolition of any

For peer review only - http://bmjopen.bmj.com/site/about/guidelines.xhtml

preconditioning response. A recent meta-analysis in children determined that RIPC has a cardioprotective effect, with reduced troponin release, lower inotrope scores and reduced paediatric intensive care unit (PICU) stay following surgery [35] but was unable to include the largest trial in most analyses due to a lack of suitable published data.

Rationale

In this trial, we will test whether in young children undergoing primary repair of tetralogy of Fallot or closure of an isolated VSD, the two most common congenital heart defects requiring surgery [1], adequately delivered RIPC, compared with sham inflation-deflation cycles, improves myocardial protection. The design will enable evaluation of the effects of RIPC in children with and without preoperative cyanosis [34]; most patients with tetralogy of Fallot have chronic hypoxaemia whilst those with a VSD are not usually cyanotic and both groups undergo surgery at a similar age. We will use a more intensive two cuff technique [18], applying a concurrent stimulus to both lower limbs to compensate for the lower skeletal muscle mass in young children. We will address methodological concerns by using a pressure-controlled tourniquet system set to at least 50mmHg above systolic pressure [27], avoiding propofol anaesthesia [30,31], and not enrolling neonates or other infants less than three months old [26]. We will only seek to exploit the first window of preconditioning, performing the intervention under general anaesthesia prior to sternotomy, as the second window would require RIPC at least 12 hours prior to surgery [13] which may be logistically challenging, distressing to the child and their parents, and lead to incomplete intervention or withdrawal. Finally, this trial will be the first multi-centre cardiac surgical trial in children in the UK [36] and act as a primer for the development of a network for the design and conduct of multi-centre phase III trials in paediatric cardiac surgery in the UK and Ireland.

For peer review only

METHODS AND ANALYSIS

Design

The Bilateral Remote Ischaemic Conditioning in Children (BRICC) trial is a two-centre, double-blind, parallel arm, randomised controlled trial to investigate the effects of RIPC and the impact of cyanosis on myocardial protection in young children undergoing elective cardiac surgery. It will be conducted through the Birmingham Clinical Trials Unit (BCTU), a UKCRC-registered clinical trials unit with expertise in surgical and paediatric trials.

Inclusion and exclusion criteria

Inclusion criteria: All infants and young children, aged 3 months to 3 years at the time of surgery, undergoing either primary repair of tetralogy of Fallot or surgical closure of a VSD, with or without concomitant atrial septal defect (ASD) closure or pulmonary artery repair/augmentation, at Birmingham Children's Hospital or Leeds Children's Hospital will be included. Only patients with the most common form of tetralogy of Fallot will be included; variants such as absent pulmonary valve syndrome, pulmonary atresia with major aortopulmonary collateral arteries, or with an atrioventricular septal defect will not be included.

Exclusion criteria: The following children will be excluded from the study:

- Those requiring an additional procedure (other than ASD closure or pulmonary artery repair/augmentation) at the time of primary repair eg. mitral repair, aortic arch repair.
- Those with significant airway or parenchymal lung disease, bleeding disorder or a recent ischaemic event.
- Those who have undergone a previous cardiac surgical procedure with cardioplegic arrest.
- Those presenting in a critical condition and requiring emergency surgery.

For peer review only - http://bmjopen.bmj.com/site/about/guidelines.xhtml

- Those for whom the parents are unwilling or unable to give informed consent.

Recruitment

Both tetralogy of Fallot and VSD are congenital heart defects that usually present with gradual onset of symptoms such as failure to thrive, difficulty feeding, dyspnoea or cyanosis. The referral pathway is therefore predictable with most children undergoing elective surgery following a period of medical therapy to allow them to grow; some children may require a palliative procedure prior to repair, notably right ventricular outflow tract (RVOT) stenting for cyanosis in tetralogy of Fallot [37], or pulmonary artery banding to reduce pulmonary overcirculation with an unrestrictive VSD. All eligible patients will be identified from the multi-disciplinary team meeting, surgical clinics or waiting lists by the principal investigators at each site, and their parents approached to ascertain interest in the trial. They will be provided with a Parent/Guardian Information Sheet (appendix A-B) either in the clinic/ward or sent in the post and given at least 24 hours to consider their child's participation and ask questions. Written informed consent will be obtained by a Consultant Surgeon prior to enrolment (appendix C-D). The participant pathway through the trial is shown in figure 1.

For peer review only

Randomisation and blinding

On the day of surgery, participants will be randomised in a 1:1 ratio to either RIPC or sham procedure using a secure online randomisation system, with a minimisation algorithm incorporating the following factors:

- congenital heart defect: tetralogy of Fallot or VSD,
- presence of an RVOT stent in patients with tetralogy of Fallot, and
- surgical centre: Birmingham or Leeds.

For peer review only - http://bmjopen.bmj.com/site/about/guidelines.xhtml

To avoid any possibility of the allocation becoming predictable, a random element will be included in the algorithm. If online randomisation is unavailable, a telephone helpline with emergency paper randomisation will be used. An independent healthcare professional, trained and competent in delivering the trial intervention, will perform the randomisation and administer the allocated treatment according to a standard operating procedure; the research nurse, surgical, anaesthetic, perfusion and PICU teams involved in the child's care will remain blinded to group allocation throughout the trial.

For peer review only

Treatment arms

Intervention arm: After induction of anaesthesia but prior to sternotomy, the treatment group will receive RIPC induced by three cycles of 5-minutes ischaemia and 5-minutes reperfusion [38]. Ischaemia will be induced simultaneously in both lower limbs using the PTSii system (Delfi Medical Innovations, Vancouver), a state-of-the-art digital tourniquet with precise control of occlusion pressure. Age-appropriate PediFit cuffs, with contour limb protection sleeves, will be placed around both thighs and inflated to at least 50mmHg above systolic pressure measured in real-time via the arterial line during the ischaemia phase of each cycle. If one lower limb is unavailable, eg. required for vascular access during the intervention period, one cuff may be placed on the upper arm instead. In addition, a dummy limb will be placed between the patient's legs to maintain blinding (see control arm below). Continual loss of arterial flow will be confirmed by distal pulse oximetry during each limb occlusion cycle, visible only to the person applying the intervention [25]; if the distal trace is not rapidly lost, the cuff will be tightened or the inflation pressure increased to achieve arterial occlusion. If pulse oximetry is not available, a clinical assessment will be made to determine whether there is loss of arterial flow (decreased lower limb temperature to touch, marked prolongation of capillary refill time) and reperfusion (increased lower limb temperature +/- blushing) during each cycle. Once the intervention has begun, each cuff

For peer review only - http://bmjopen.bmj.com/site/about/guidelines.xhtml

must be kept on the same limb to ensure repeated doses of IR to the same muscle mass. Blinding will be maintained by covering the child with a surgical drape from above the nipples downwards including all four limbs throughout the period of cuff application, intervention, and removal.

Control arm: Contour limb protection sleeves will be placed around both thighs but the PediFit cuffs will be attached to the dummy limb (43x300mm polyethylene tubing) placed between the patient's legs. Three sham inflation-deflation cycles will be performed using the PTSii system. Pulse oximetry monitoring will be reviewed by the person applying the intervention only, but no loss of trace will be observed during the cycles. As above, the child will be covered with a surgical drape to maintain blinding before, during and after the sham intervention.

Adherence to treatment will be defined as receiving the allocated treatment, and in the intervention arm, with loss of arterial flow (pulse oximetry or clinical assessment, if required) during each period of limb ischaemia.

Common aspects of care

Anaesthesia: Anaesthesia will be conducted at the discretion of the consultant anaesthetist and involve a balanced technique using volatile and intravenous anaesthesia and adjuncts, opioid pain relief and muscle relaxants, within the limits of the protocol. Propofol will not be used for induction or maintenance of anaesthesia; isoflurane will be the preferred volatile anaesthetic agent and end-tidal partial pressure will be recorded at the end of RIPC administration. Phenylepherine will be used for vasoconstriction, as required. Routine monitoring will include continuous invasive arterial and central venous pressures, other cardiac output variables, urine output, blood gas analysis and near-patient clotting profile [39]. Systemic anticoagulation will be achieved with heparin prior to institution of cardiopulmonary bypass (CPB) and reversed with protamine after the termination of CPB.

For peer review only - http://bmjopen.bmj.com/site/about/guidelines.xhtml

Surgery & Perfusion: Repair of the congenital heart defect(s) will be performed following best clinical practice. After transfer to the operating room, the surgical checklist will be completed, the patient prepped and draped, and the chest opened through a median sternotomy. Standardised cardiopulmonary bypass will be established between the vena cavae and the ascending aorta with moderate hypothermia. An aortic cross-clamp will be applied to the proximal ascending aorta with intermittent antegrade cold cardioplegia given via the aortic root for myocardial protection; patients undergoing VSD closure will usually receive a single dose, whilst those with tetralogy of Fallot will typically require an additional dose. Removal of the aortic cross-clamp with myocardial reperfusion will be considered as time zero for the recording of postoperative events. Following completion of the repair and rewarming, CPB will be weaned and discontinued. In the event of difficulty separating from bypass or marked haemodynamic instability, subjective and objective measures of ventricular function will be obtained, and inotropic support instituted at the discretion of the blinded operating team. Once haemodynamic stability and haemostasis have been achieved, the chest will be closed at the discretion of the surgical team and the patient transferred to the PICU. Standard postoperative care will proceed with anticipated removal of the arterial line at 12 hours following surgery, removal of the central line at 24 hours, and transfer to the ward once routine PICU discharge criteria have been met. All decisions regarding escalation of therapy will be made by the blinded clinical team responsible for the care of the child without influence from the researchers.

Trial investigations

The schedule for the intervention and collection of outcome data, blood and tissue samples is shown in table 1.

Data collection: Clinical data will be collected by the Research Nurse before, during and after surgery. Inotrope usage in the first 12 hours will be used to generate a vasoactive inotrope

For peer review only - http://bmjopen.bmj.com/site/about/guidelines.xhtml

score (VIS) (μg/kg/min) [40,41]. Arterial lactate and central venous oxygen saturations will be recorded prior to surgery and at 3, 6, 9 and 12 hours. Length of stay in PICU (hours) and hospital (days) following surgery will be documented. Preoperative haematocrit and resting oxygen saturations in air will be used as markers of the degree of exposure to cyanosis. In Birmingham only, cardiac output will be measured over the first 12 hours following reperfusion using ICON (Osypka Medical, Berlin), a non-invasive technique for electrical velocimetry which has been validated in young children [42-44].

Blood samples: Blood will be drawn from indwelling arterial or central venous lines at baseline (after induction of anaesthesia but prior to sternotomy) and at 3, 6, 12 and 24 hours after reperfusion. Plasma samples for hs-troponin-T (Elecsys Tn-T HS, Roche, Basel) will be collected in paediatric lithium heparin tubes, centrifuged, split into two aliquots and stored at -80°C in remotely-monitored freezers at each site until transfer for analysis at one of two core labs (Sandwell General Hospital, Birmingham or Russells Hall Hospital, Dudley). Samples will be analysed in batches approximately every eight months so that data on the primary outcome will be available to the Data Monitoring Committee prior to each meeting.

Tissues samples: In Birmingham only, myocardial biopsies will be obtained for a metabolic sub-study. Right atrial samples will be taken soon after aortic cross-clamping (onset ischaemia) and just before its release (late ischaemia) to assess metabolic changes in the myocardium during the period of ischaemia. In a subset of patients with tetralogy of Fallot, several samples of hypertrophic septoparietal trabeculae of the right ventricular infundibulum will be obtained at various points during ischaemia, whenever routinely resected. Specimens will be briefly washed in saline, promptly snap-frozen in liquid nitrogen and stored at -80°C until transfer to the Phenome Centre Birmingham for metabolic phenotyping. Analysis of these samples is exploratory and will follow a separate analytical plan (see sub-study below).

For peer review only

Outcome measures and follow-up

Primary outcome: Reduction in area under the time-concentration curve (AUC) for high-sensitivity troponin-T release in the first 24 hours after aortic cross-clamp release (reperfusion) as a marker of myocardial injury.

Secondary outcomes

- Peak hs-troponin-T in the first 12 hours
- Total vasoactive inotrope score in the first 12 hours
- Arterial lactate and central venous oxygen saturations in the first 12 hours
- Length of postoperative stay in the PICU
- Length of postoperative stay in the hospital

Exploratory outcome: Cardiac index in the first 12 hours measured using ICON (Birmingham only).

Follow-up: until discharge from hospital or 30 days, whichever is sooner.

Analysis

Sample size: It is hypothesised that RIPC will reduce the AUC for hs-troponin-T release in the first 24 hours compared with controls, but that exposure to hypoxaemia may impact on this reduction. The sample size proposed here will be sufficient to detect a 35% reduction in postoperative troponin release, assuming a mean release of 350 µg/L/h in the control group compared with 228 µg/L/h in the RIPC group (extrapolated from the similarly mixed cohort of hypoxic and non-hypoxic children in Toronto [21]), with a variability of 220 µg/L/h [24]. A sample size of at least 52 children per treatment group is needed to have a power of 80% and a significance level of 0.05 (2-sided). We therefore will recruit at least 104 children (up to 120 children to allow for dropouts) randomised in a 1:1 ratio between RIPC and control.

For peer review only - http://bmjopen.bmj.com/site/about/guidelines.xhtml

Expected recruitment rate: The paediatric cardiac surgery units in Birmingham and Leeds are ideally placed to conduct clinical trials. Over the preceding three years, 99-135 children per annum have undergone surgical repair of either tetralogy of Fallot (mean 50) or VSD (mean 69) across the two sites [1]. The only previous interventional trial in cardiac surgery at Birmingham Children's Hospital recruited 22 (79%) of the 28 patients approached [45]. None of the other UK paediatric cardiac surgery RCTs have reported recruitment rates [36] but our predictions are comparable to those obtained from similar trials in North America which ranged from 62% to 84% [27,36,46]. We will maintain a screening log to document exclusions and reasons given by parents who decline to participate; this will be available to the Trial Management Committee who will monitor recruitment targets and advise on any changes to the protocol.

Statistical analysis: Analysis of the main outcome measures will be performed according to the intention-to-treat principle and any non-adherence to the allocated group documented. The primary analysis will assess whether RIPC reduces AUC for troponin release in the first 24 hours compared with control. The primary outcome measure will be calculated using the trapezoidal method and presented as an adjusted mean difference between groups along with the 95% confidence interval estimated using a linear regression model (adjusting for the minimisation variables and baseline troponin). For the secondary outcomes, continuous data items (eg. peak troponin) will also be analysed using a linear regression model. Continuous outcomes measured across more than three time points (eg. arterial lactate and central venous oxygen saturations) will be analysed using mixed effect repeated measures models. Time to event data outcomes will be analysed using a Cox regression model. Test of interactions will be employed to assess whether there is evidence that the treatment effect differs between cyanotic and acyanotic patients. P-values will be reported from two-sided tests at the 5% significance level. A detailed statistical analysis plan is under development and will be approved prior to database lock. The Chief Investigator and trial statisticians will have access to the final trial dataset.

For peer review only - http://bmjopen.bmj.com/site/about/guidelines.xhtml

Monitoring

Assessment and management of risk: No adverse events directly attributed to the application of a tourniquet cuff during RIPC were identified in a meta-analysis of 1,762 adults and children undergoing cardiac surgery in 25 trials [29] nor in any of the notable trials published since [19,20,27,28]. Risk to participants therefore is deemed to be minimal and the trial is categorised as type A: no higher than the risk of standard medical care. In the event of concern, parents will be signposted to their cardiac specialist nurse, their General Practitioner, or the hospital Patient Advice Liaison Service, as appropriate.

Trial Management Committee: The trial will be overseen by a committee meeting approximately every four months during the trial. It will comprise clinicians, trialists and scientists involved in the set-up and running of the trial including representation from both trial sites. During recruitment, the protocol may be reviewed considering achievement of recruitment targets, evidence from new publications, and feedback from parents approached for the trial; ethical approval for amendments to the protocol will be sought, as required.

Data Monitoring Committee: An independent Data Monitoring Committee will meet approximately every eight months during recruitment to review efficacy and safety data, according to a predefined charter (appendix E). Members are an academic consultant cardiac surgeon as chair, a consultant in paediatric cardiac intensive care, and a statistician. Analysis of hs-troponin-T for the primary outcome will be performed in batches prior to each meeting and all unblinded safety and efficacy data made available to the committee.

Safety reporting: Adverse events will be recorded and reported in accordance with the sponsor's Code of Practice for Research. Participants in the study are undergoing open heart surgery and therefore adverse events are anticipated. The following serious adverse events will be reviewed by the Chief Investigator and reported to the sponsor within 48 hours of identification: death; requirement for extracorporeal life support; evidence of a major neurological event; and need for further surgery in the early post-operative period.

For peer review only - http://bmjopen.bmj.com/site/about/guidelines.xhtml

Data collection and management: All data will be entered onto the BRICC trial database, a password protected electronic database held on secure University of Birmingham servers for trial data with access limited to BCTU members of staff working on the trial. All paper case report forms will be stored securely in the Research offices at Birmingham Women's and Children's NHS Foundation Trust and Leeds Teaching Hospitals NHS Trust. Data will be semi-anonymised by removing non-essential potentially identifiable patient information; blood and tissue samples will be labelled with the unique trial ID number, date, and time of collection. Adherence to trial processes will be audited by the independent Clinical Research Compliance team at the University of Birmingham.

For peer review only

Sub-studies

Metabolic phenotyping: No study in children has previously examined the impact of RIPC on myocardial metabolism or its interaction with chronic hypoxaemia. Therefore, building on metabolic phenotyping in animal models of IR injury [47], we will analyse intraoperative biopsies to identify changes in myocardial metabolic pathways that occur during ischaemia. In brief, tissue extracts will be analysed using ultra high performance liquid chromatography-mass spectrometry (UHPLC-MS) in two independent discovery and validation phases. Two complementary assays will be applied, (1) HILIC assay to study water-soluble metabolites, including those present in glycolysis and the TCA cycle, and (2) C_{18} reversed-phase assay to determine changes in lipids during ischaemia [48]. The eluents from UHPLC columns will be introduced directly into an electrospray Q Exactive Mass Spectrometer (Thermo Scientific, UK) and data acquired in the *m/z* range 70-1000. The impact of RIPC on metabolism and how any changes may be attenuated by preoperative cyanosis, will be assessed through robust statistical analysis using correction for multiple testing and pathway enrichment analysis.

For peer review only - http://bmjopen.bmj.com/site/about/guidelines.xhtml

Qualitative: We will explore parents' perspectives on decision-making about their child's participation in a clinical trial as part of their elective cardiac surgery. Parents of children approached to participate in the trial, both consenters and decliners, will be contacted following surgery and asked to participate in semi-structured interviews which, with written informed consent, will be digitally audio-recorded, intelligently transcribed, and thematically analysed. The findings will enhance our understanding of the factors that influence parents' decision-making and be used to inform the design and conduct of future trials. The BRICC trial is a suitable vehicle for this sub-study as the intervention presents minimal risk, the surgery is performed electively, and the operations included have a low predicted mortality (STAT categories 1-2) [49].

Patient and Public Involvement

Patient and Public Involvement (PPI) has been a central component in the development, conduct and planned reporting of this trial since its inception. Parents of children who had previously undergone cardiac surgery at Birmingham Children's Hospital were contacted through *Young at Heart*, the local children's heart charity. Four parents reviewed the draft parent information sheet and consent form for the trial, making suggestions to improve clarity and readability for a lay audience, which were incorporated into the final versions. The parent information sheets, consent forms and protocol for the qualitative sub-study were also reviewed by the Young Person's Steering Group in the West Midlands. The outcomes of the trial will be communicated by individual parent feedback and a charity newsletter, both of which will be produced in collaboration with the charity and parents. Early user involvement was funded by a bursary from the NIHR Research Design Service West Midlands and all PPI was costed using the INVOLVE Calculator according to the NIHR's Budgeting for Involvement [50].

For peer review only

ETHICS AND DISSEMINATION

This clinical trial was approved by the West Midlands-Solihull NHS Research Ethics Committee (16/WM/0309) on 5 August 2016 and the NHS Health Research Authority (200876) on 19 August 2016. It is sponsored by the University of Birmingham (RG_14-025, email: researchgovernance@contacts.bham.ac.uk, telephone: +44 (0) 121 415 8011), registered on the NIHR Clinical Research Network portfolio (32330), and approved by the NHS Research & Development departments at Birmingham Children's Hospital (1845) and Leeds Children's Hospital (PA17/67348). Regulatory approval from the Medicines and Healthcare products Regulatory Agency (MHRA) was not required as this trial is not a CTIMP. The first patient was randomised on 24 October 2016 and recruitment is currently ongoing.

Changes to the protocol since original ethical approval

Since the original ethical approval, four substantial amendments to the protocol have been sought and approved with the following significant changes:

- Add 'with/without concomitant pulmonary artery repair/augmentation' to the inclusion criteria, to allow inclusion of those with pulmonary artery disease within the spectrum of tetralogy of Fallot and those with VSD who had previous pulmonary artery banding (December 2016).
- Add Leeds Children's Hospital as the second site and extend the duration of recruitment (February 2018).
- Remove 'known major chromosomal defect' as an exclusion criterion; although originally included as per previous paediatric trials [21,27], following discussion with Prof Andrew Redington (Cincinnati, OH), principal investigator of these trials, it became clear that there was no biological reason relating to RIPC to exclude these patients (February 2018).

For peer review only - http://bmjopen.bmj.com/site/about/guidelines.xhtml

- Add Russells Hall Hospital, Dudley as a second core laboratory to maintain internal validity, as Sandwell General Hospital, Birmingham changed their troponin analysis platform during the trial (November 2019).

Dissemination plan

The findings of the clinical trial and sub-studies will be submitted for presentation at national and international meetings and manuscripts prepared for submission to leading journals. The authorship of the final trial report will include all members of the trial management committee and named collaborators. The anonymised individual participant data collected during the trial will be available on request following publication of the study results.

Parents of children participating in the trial will be informed of the results in writing once data analysis is complete. The local charity Young at Heart will also report the outcomes in their newsletter to reach a wider audience of those affected by congenital heart disease. PPI collaborators will be invited to participate in producing both the parent feedback and charity newsletter.

The first author is Chief Investigator of the trial and takes responsibility for the integrity of this protocol report, which adheres to the SPIRIT recommendations [51]. All authors have read and agree to the manuscript as written.

For peer review only

For peer review only - http://bmjopen.bmj.com/site/about/guidelines.xhtml

Abbreviations

ASD	Atrial Septal Defect
AUC	Area Under the time-concentration Curve
BCTU	Birmingham Clinical Trials Unit
BRICC	Bilateral Remote Ischaemic Conditioning in Children
CPB	Cardiopulmonary Bypass
CTIMP	Clinical Trial of an Investigational Medicinal Product
IR	Ischaemia-Reperfusion
NHS	National Health Service
NIHR	National Institute for Health Research
PICU	Paediatric Intensive Care Unit
PPI	Patient and Public Involvement
RCT	Randomised Controlled Trial
RIPC	Remote Ischaemic Preconditioning
RVOT	Right Ventricular Outflow Tract
UHPLC-MS	Ultra High Performance Liquid Chromatography-Mass Spectrometry
VIS	Vasoactive Inotrope Score
VSD	Ventricular Septal Defect

For peer review only

Acknowledgements: We are grateful to the members of the independent Data Monitoring Committee for their guidance and oversight throughout the trial: Prof Gavin J Murphy (Chair), University of Leicester, UK; Dr Katherine L Brown, Great Ormond Street Hospital, London, UK; and Dr Peter Nightingale (Statistician), Queen Elizabeth Hospital Birmingham, UK.

We thank Dr John V Pappachan (Southampton, UK) for guidance on the delivery of RIPC, Prof Andrew N Redington (Cincinnati, OH) for advice on RIPC and the exclusion criteria, and Prof Peter Brocklehurst, Director of BCTU, for his support for the trial. We are grateful to our surgical colleagues in Birmingham: Ms Natasha E Khan, Mr Phil Botha, Prof David J Barron & Dr Adrian Crucean, and Leeds: Mr Osama Jaber, Mr Imre Kassai & Mr Guiseppe Pelella for their input to the evolution of the trial. We thank Dr Oliver Stumper and Dr Anna N Seale, Birmingham for their advice on ICON monitoring and echo imaging, respectively. We thank Matt Hill and Alicia Gill at BCTU for programming and statistical support, respectively, and Collette Spencer for setting up the study in Leeds. We are most grateful to Martina Ponsonby and the trustees of *Young at Heart* for their feedback on the study documents.

Author contributions: NED, MM and TJJ conceptualised the trial. NED, RB, KPM, JM, NJI, PK and TJJ designed the trial with additional critical input from RLW, JS, CVD and MM. NED, RLW and NJI developed the statistical analysis. NED and WBD designed the metabolic phenotyping sub-study. All authors contributed to writing of the paper.

Funding: This work is supported by an Intermediate Clinical Research Fellowship from the British Heart Foundation [FS/15/49/31612] awarded to Nigel Drury. Lay review of the parent information sheets and consent forms was funded by a Patient & Public Involvement Bursary Award [RDS/WM-1318] from the NIHR Research Design Service in the West Midlands. The Institute of Cardiovascular Sciences is supported by an Accelerator Award from the British Heart Foundation [AA/18/2/34218]. Phenome Centre Birmingham was constructed through a grant from the Medical Research Council in the UK [MR/M009157/1]. Neither the sponsor nor funders had any role in the design of this study and will not have any role during its

For peer review only - http://bmjopen.bmj.com/site/about/guidelines.xhtml

execution, analyses, interpretation of the data, or decision to submit the results for publication.

Competing interests: None declared.

Ethics approval: West Midlands-Solihull National Health Service Research Ethics Committee (16/WM/0309) on 5 August 2016. This manuscript is based on protocol v1.8 dated 22 November 2019, which was approved by the ethics committee on 5 December 2019.

For peer review only

For peer review only - http://bmjopen.bmj.com/site/about/guidelines.xhtml

REFERENCES

1. NICOR National Institute for Cardiovascular Outcomes Research, UCL. Congenital Heart Disease. https://nicor4.nicor.org.uk/CHD/an_paeds.nsf/vwContent/home [accessed March 17, 2020]

2. Doenst T, Schlensak C, Beyersdorf F. Cardioplegia in pediatric cardiac surgery: do we believe in magic? *Ann Thorac Surg* 2003; 75: 1668-77.

3. del Nido PJ, Mickle DA, Wilson GJ, Benson LN, Weisel RD, Coles JG, Trusler GA, Williams WG. Inadequate myocardial protection with cold cardioplegia arrest during repair of tetralogy of Fallot. *J Thorac Cardiovasc Surg* 1988; 95: 223-9.

4. Allen BS. Pediatric myocardial protection: where do we stand? *J Thorac Cardiovasc Surg* 2004; 128: 11-13.

5. Mildh LH, Pettilä V, Sairanen HI, Rautiainen PH. Cardiac Troponin T levels for risk stratification in pediatric open heart surgery. *Ann Thorac Surg* 2006; 82: 1643-9.

6. Ma M, Gauvreau K, Allan CK et al. Causes of death after congenital heart surgery. *Ann Thorac Surg* 2007; 83: 1438-45.

7. Gaies M, Pasquali SK, Donohue JE et al. Seminal postoperative complications and mode of death after pediatric cardiac surgical procedures. *Ann Thorac Surg* 2016; 102: 628-35.

8. Imura H, Caputo M, Parry A, Pawade A, Angelini GD, Suleiman MS. Age-dependent and hypoxia-related differences in myocardial protection during paediatric open-heart surgery. *Circulation* 2001; 103: 1551-6.

9. Najm HK, Wallen WJ, Belanger MP, Williams WG, Coles JG, Van Arsdell GS, Black MD, Boutin C, Wittnich C. Does the degree of cyanosis affect myocardial adenosine triphosphate levels and function in children undergoing surgical procedures for congenital heart disease? *J Thorac Cardiovasc Surg* 2000; 119: 515-24.

For peer review only - http://bmjopen.bmj.com/site/about/guidelines.xhtml

10. Immer FF, Stocker F, Seiler AM et al. Troponin-I for prediction of early postoperative course after pediatric cardiac surgery. *J Am Coll Cardiol* 1999; 33: 1719-23.

11. Drury NE, Yim I, Patel AJ et al. Cardioplegia in paediatric cardiac surgery: a systematic review of randomized controlled trials. *Interact Cardiovasc Thorac Surg* 2019; 28: 144-150.

12. Hausenloy DJ, Yellon DM. Remote ischaemic preconditioning: underlying mechanisms and clinical application. *Cardiovasc Res* 2008; 79: 377-86.

13. Hausenloy DJ, Yellon DM. The Second Window of Preconditioning (SWOP): where are we now? *Cardiovasc Drugs Ther* 2010; 24: 235-54.

14. Kharbanda RK, Mortensen UM, White PA, Kristiansen SB, Schmidt MR, Hoschtitzky JA, Vogel M, Sorensen K, Redington AN, MacAllister R. Transient limb ischemia induces remote ischemic preconditioning in vivo. *Circulation* 2002; 106: 2881-3.

15. Hausenloy DJ, Mwamure PK, Venugopal V, Harris J, Barnard M, Grundy E, Ashley E, Vichare S, Di Salvo C, Kolvekar S, Hayward M, Keogh B, MacAllister RJ, Yellon DM. Effect of remote ischaemic preconditioning on myocardial injury in patients undergoing coronary artery bypass graft surgery: a randomised controlled trial. *Lancet* 2007; 370: 575-9.

16. Rahman IA, Mascaro JG, Steeds RP, Frenneaux MP, Nightingale P, Gosling P, Townsend P, Townend JN, Green D, Bonser RS. Remote ischaemic preconditioning in human coronary artery bypass surgery: from promise to disappointment? *Circulation* 2010; 122 (11 Suppl): S53-9.

17. Thielmann M, Kottenberg E, Kleinbongard P, Wendt D, Gedik N, Pasa S, Price V, Tsagakis K, Neuhauser M, Peters J, Jakob H, Heusch G. Cardioprotective and prognostic effects of remote ischaemic preconditioning in patients undergoing coronary artery bypass surgery: a single-centre randomised, double-blind, controlled trial. *Lancet* 2013; 382: 597-604.

For peer review only

18. Candilio L, Malik A, Ariti C, Barnard M, Di Salvo C, Lawrence D, Hayward M, Yap J, Roberts N, Sheikh A, Kolvekar S, Hausenloy DJ, Yellon DM. Effect of remote ischaemic preconditioning on clinical outcomes in patients undergoing cardiac bypass surgery: a randomised controlled clinical trial. *Heart* 2015; 101: 185-92.

19. Meybohm P, Bein B, Brosteanu O, Cremer J, Gruenewald M, Stoppe C, Coburn M, Schaelte G, Böning A, Niemann B, Roesner J, Kletzin F, Strouhal U, Reyher C, Laufenberg-Feldmann R, Ferner M, Brandes IF, Bauer M, Stehr SN, Kortgen A, Wittmann M, Baumgarten G, Meyer-Treschan T, Kienbaum P, Heringlake M, Schön J, Sander M, Treskatsch S, Smul T, Wolwender E, Schilling T, Fuernau G, Hasenclever D, Zacharowski K. A multicenter trial of remote ischemic preconditioning for heart surgery. *N Engl J Med* 2015; 373: 1397-407.

20. Hausenloy DJ, Candilio L, Evans R, Ariti C, Jenkins DP, Kolvekar S, Knight R, Kunst G, Laing C, Nicholas J, Pepper J, Robertson S, Xenou M, Clayton T, Yellon DM. Remote ischemic preconditioning and outcomes of cardiac surgery. *N Engl J Med* 2015; 373: 1408-17.

21. Cheung MM, Kharbanda RK, Konstantinov IE, Shimizu M, Frndova J, Li J, Holtby HM, Cox PN, Smallhorn JF, Van Arsdell GS, Redington AN. Randomised controlled trial of the effects of remote ischemic preconditioning on children undergoing cardiac surgery: first clinical application in humans. *J Am Coll Cardiol* 2006; 47: 2277-82.

22. Zhou W, Zeng D, Chen R, Liu J, Yang G, Liu P, Zhou X. Limb ischemic preconditioning reduces heart and lung injury after an open heart operation in infants. *Pediatr Cardiol* 2010; 31: 22-29.

23. Luo W, Zhu M, Huang R, Zhang Y. A comparison of cardiac post-conditioning and remote pre-conditioning in paediatric cardiac surgery. *Cardiol Young* 2011; 21: 266-70.

For peer review only

24. Lee JH, Park YH, Byon HJ, Kim HS, Kim Cs, Kim JT. Effect of remote ischaemic preconditioning on ischaemia-reperfusion injury in pulmonary hypertensive infants receiving ventricular septal defect repair. *Br J Anaesth* 2012; 108: 223-8.

25. Pepe S, Liaw NY, Hepponstall H, Sheeran FL, Yong MS, d'Udekem Y, Cheung MM, Konstantinov IE. Effect of remote ischemic preconditioning on phosphorylated protein signalling in children undergoing Tetralogy of Fallot repair: a randomised controlled trial. *J Am Heart Assoc* 2013; 2: e000095.

26. Jones BO, Pepe S, Sheeran FL, Donath S, Hardy P, Shekerdemian L, Penny DJ, McKenzie I, Horton S, Brizard CP, d'Udekem Y, Konstantinov IE, Cheung MM. Remote ischemic preconditioning in cyanosed neonates undergoing cardiopulmonary bypass: a randomized controlled trial. *J Thorac Cardiovasc Surg* 2013; 146: 1334-40.

27. McCrindle BW, Clarizia NA, Khaikin S, Holtby HM, Manlhiot C, Schwartz SM, Caldarone CA, Coles JG, Van Arsdell G, Scherer SW, Redington AN. Remote ischemic preconditioning in children undergoing cardiac surgery with cardiopulmonary bypass: a single-center double-blinded randomized trial. *J Am Heart Assoc* 2014; 3: e000964.

28. Wu Q, Wang T, Chen S, Zhou Q, Li H, Hu N, Feng Y, Dong N, Yao S, Xia Z. Cardiac protective effects of remote ischaemic preconditioning in children undergoing tetralogy of Fallot repair surgery: a randomized controlled trial. *Eur Heart J* 2018; 39: 1028-37.

29. Haji Mohd Yasin NA, Herbison P, Saxena P, Praporski S, Konstantinov IE. The role of remote ischemic preconditioning in organ protection after cardiac surgery: a meta-analysis. *J Surg Res* 2014; 186: 207-16.

30. Kottenberg E, Musiolik, Thielmann M, Jakob H, Peters J, Heusch G. Interference of propofol with signal transducer and activator of transcription 5 activation and cardioprotection by remote ischemic preconditioning during coronary artery bypass grafting. *J Thorac Cardiovasc Surg* 2014; 147: 376-82.

For peer review only - http://bmjopen.bmj.com/site/about/guidelines.xhtml

31. Heusch G, Gersh BJ. ERICCA and RIPHeart: two nails in the coffin for cardioprotection by remote ischemic conditioning? Probably not! *Eur Heart J* 2016; 37: 200-2.

32. Awad WI, Shattock MJ, Chambers DJ. Ischemic preconditioning in immature myocardium. *Circulation* 1998; 98: II206-13.

33. Schmidt MR, Støttrup NB, Michelsen MM, Contractor H, Sørensen KE, Kharbanda RK, Redington AN, Bøtker HE. Remote ischemic preconditioning impairs ventricular function and increases infarct size after prolonged ischemia in the isolated neonatal rabbit heart. *J Thorac Cardiovasc Surg* 2014; 147: 1049-55.

34. Konstantinov IE. Remote ischemic preconditioning in children with cyanotic heart disease: lost in translation? *J Thorac Cardiovasc Surg* 2013; 145: 613-4.

35. Tan W, Zhang C, Liu J, Li X, Chen Y, Miao Q. Remote Ischemic Preconditioning has a Cardioprotective Effect in Children in the Early Postoperative Phase: A Meta-Analysis of Randomized Controlled Trials. *Pediatr Cardiol* 2018; 39: 617-26.

36. Drury NE, Patel AJ, Oswald NK et al. Randomized controlled trials in children's heart surgery in the 21st century: a systematic review. *Eur J Cardiothorac Surg* 2018; 53: 724-31.

37. Stumper O, Ramchandani B, Noonan P, Mehta C, Bhole V, Reinhardt Z, Dhillon R, Miller P, de Giovanni J. Stenting of the right ventricular outflow tract. *Heart* 2013; 99: 1603-8.

38. Pickard JM, Bøtker HM, Crimi G, Davidson B, Davidson SM, Dutka D, Ferdinandy P, Ganske R, Garcia-Dorado D, Giricz Z, Gourine AV, Heusch G, Kharbanda R, Kleinbongard P, MacAllister R, McIntyre C, Meybohm P, Prunier F, Redington A, Robertson NJ, Suleiman MS, Vanezis A, Walsh S, Yellon DM, Hausenloy DJ. Remote ischemic preconditioning: from experimental observation to clinical application: report from the 8th biennial Hatter Cardiovascular Institute Workshop. *Basic Res Cardiol* 2015; 110: 453.

For peer review only - http://bmjopen.bmj.com/site/about/guidelines.xhtml

39. Checketts MR, Alladi R, Ferguson K, Gemmell L, Handy JM, Klein AA, Love NJ, Misra U, Morris C, Nathanson MH, Rodney GE, Verma R, Pandit JJ. Recommendations for Standards of Monitoring During Anaesthesia and Recovery 2015: Association of Anaesthetists of Great Britain and Ireland. *Anaesthesia* 2016; 71: 85-93.

40. Gaies MG, Gurney JG, Yen AH, et al: Vasoactive-inotropic score as a predictor of morbidity and mortality in infants after cardiopulmonary bypass. *Pediatr Crit Care Med* 2010; 11: 234-8.

41. Gaies MG, Jeffries HE, Niebler RA et al. Vasoactive-inotropic score is associated with outcome after infant cardiac surgery: an analysis from the Pediatric Cardiac Critical Care Consortium and Virtual PICU system registries. *Pediatr Crit Care Med* 2014; 15: 529-37.

42. Norozi K, Beck C, Osthaus WA, Wille I, Wessel A, Bertram H. Electrical velocimetry for measuring cardiac output in children with congenital heart disease. *Br J Anaesth* 2008; 100: 88-94.

43. Schubert S, Schmitz T, Weiss M, Nagdyman N, Huebler M, Alexi-Meskishvili V, Berger F, Stiller B. Continuous, non-invasive techniques to determine cardiac output in children after cardiac surgery: evaluation of transoesophageal Doppler and electric velocimetry. *J Clin Monit Comput* 2008; 22: 299-307.

44. Noonan PM, Viswanathan S, Chambers A, Stumper O. Non-invasive cardiac output monitoring during catheter interventions in patients with cavopulmonary circulations. *Cardiol Young* 2014; 24: 417-21.

45. Swindell CG, Barker TA, McGuirk SP, Jones TJ, Barron DJ, Brawn WJ, Horsburgh A, Willetts RG. Washing of irradiated red blood cells prevents hyperkalaemia during cardiopulmonary bypass in neonates and infants undergoing surgery for complex congenital heart disease. *Eur J Cardiothorac Surg* 2007; 31: 659-64.

For peer review only

46. Ohye RG, Sleeper LA, Mahony L, Newburger JW, Pearson GD, Lu M, Goldberg CS, Tabbutt S, Frommelt PC, Ghanayem NS, Laussen PC, Rhodes JF, Lewis AB, Mital S, Ravishankar C, Williams IA, Dunbar-Masterson C, Atz AM, Colan S, Minich LL, Pizarro C, Kanter KR, Jaggers J, Jacobs JP, Krawczeski CD, Pike N, McCrindle BW, Virzi L, Gaynor JW for the Pediatric Heart Network Investigators. Comparison of shunt type in the Norwood procedure for single ventricle lesions. *N Engl J Med* 2010; 362: 1980-92.

47. Chouchani ET, Pell VR, Gaude E, Aksentijević D, Sundier SY, Robb EL, Logan A, Nadtochiy SM, Ord EN, Smith AC, Eyassu F, Shirley R, Hu CH, Dare AJ, James AM, Rogatti S, Hartley RC, Eaton S, Costa AS, Brookes PS, Davidson SM, Duchen MR, Saeb-Parsy K, Shattock MJ, Robinson AJ, Work LM, Freeza C, Krieg T, Murphy MP. Ischaemic accumulation of succinate controls reperfusion injury through mitochondrial ROS. *Nature* 2014; 515: 431-5.

48. Gehmlich K, Dodd MS, Allwood JW, Kelly M, Bellahcene M, Lad HV, Stockenhuber A, Hooper C, Ashrafian H, Redwood CS, Carrier L, Dunn WB. Changes in the cardiac metabolome caused by perhexiline treatment in a mouse model of hypertrophic cardiomyopathy. *Mol Biosyst* 2015; 11: 564-73.

49. O'Brien SM, Clarke DR, Jacobs JP, Jacobs ML, Lacour-Gayet FG, Pizarro C, Welke KF, Maruszewski B, Tobota Z, Miller WJ, Hamilton L, Peterson ED, Mavroudis C, Edwards FH. An empirically based tool for analyzing mortality associated with congenital heart surgery. *J Thorac Cardiovasc Surg* 2009; 138: 1139-53.

50. Mental Health Research Network and INVOLVE. *Budgeting for involvement: Practical advice on budgeting for actively involving the public in research studies*. Mental Health Research Network, London and INVOLVE, Eastleigh, 2013.

51. Chan A-W, Tetzlaff JM, Altman DG, Laupacis A, Gøtzsche PC, Krleža-Jerić K, Hróbjartsson A, Mann H, Dickersin K, Berlin J, Doré C, Parulekar W, Summerskill W, Groves

For peer review only - http://bmjopen.bmj.com/site/about/guidelines.xhtml

T, Schulz K, Sox H, Rockhold FW, Rennie D, Moher D. SPIRIT 2013 Statement: Defining standard protocol items for clinical trials. *Ann Intern Med* 2013; 158: 200-7.

For peer review only

For peer review only - http://bmjopen.bmj.com/site/about/guidelines.xhtml

Appendices

Appendix A: Parent/guardian information leaflet: Birmingham, v1.9 dated 22 November 2019

Appendix B: Parent/guardian information leaflet: Leeds, v1.9 dated 22 November 2019

Appendix C: Parent/guardian consent form: Birmingham, v1.9 dated 22 November 2019

Appendix D: Parent/guardian consent form: Leeds, v1.9 dated 22 November 2019

Appendix E: Data Monitoring Committee charter, v1.3 dated 8 June 2018

Figure legends

Figure 1. Participant pathway from screening to end of follow-up.

PIS, parent information sheet; RA, right atrium; RIPC, remote ischaemic preconditioning; RV, right ventricle.

For peer review only

For peer review only - http://bmjopen.bmj.com/site/about/guidelines.xhtml

Table 1. Schedule of events: intervention, outcome data, blood, and tissue samples.

	Pre-operative	Pre-sternotomy	Intraoperative			On PICU admission	Time since aortic cross-clamp removal (hours)					Daily until discharge	Hospital discharge
			Onset ischaemia	During ischaemia	Late ischaemia		3	6	9	12	24		
Screening & consent	x												
Randomisation	x												
Clinical baseline data	x												
RIPC or sham intervention		x											
Blood for hs-troponin-T		x					x	x		x	x		
Arterial/venous blood gases		x					x	x	x	x			
Right atrium biopsies			x		x								
Right ventricle biopsies				x									
Clinical outcome data						x				x	x	x	x
Cardiac index (BCH only)						x	x	x	x	x			

BCH, Birmingham Children's Hospital; PICU, paediatric intensive care unit; RIPC, remote ischaemic preconditioning.

For peer review only

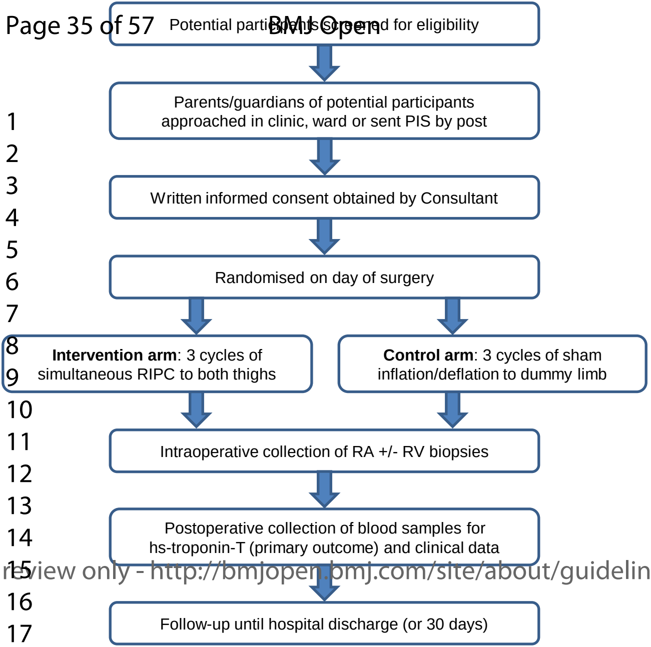

er review only - http://bmjopen.bmj.com/site/about/guideline

PARENT/GUARDIAN INFORMATION SHEET

The Bilateral Remote Ischaemic Conditioning in Children trial

Chief Investigator: Mr Nigel Drury, Consultant in Paediatric Cardiac Surgery, BCH

For peer review only

An invitation to participate in research: The Heart Surgery team at Birmingham Children's Hospital would like to invite your child to take part in a research study. Before you decide, you need to understand why the research is being done and what it would involve for you and your child. Please read the following information carefully and take time to decide whether or not you would like your child to take part. If there is anything that is not clear or you would like more information, please ask.

Why is my child being invited to take part?

Your child has been referred to the Heart Surgery team for an operation to repair one of two common congenital heart conditions: Tetralogy of Fallot (TOF) or a Ventricular Septal Defect (VSD). We are performing a clinical trial in young children with these conditions and have approached you to see if you would like your child to take part.

What is the aim of the study?

Children like yours are born with congenital heart disease and often need operations to correct the abnormality that they were born with to improve their survival. The surgery is complex and usually involves a period of support on a heart-lung machine (cardiopulmonary bypass) whilst the defect is repaired. The surgery puts a strain on your child's heart and may potentially cause damage (called ischaemia-reperfusion injury) when the blood supply to the heart is interrupted and restored. In this study, we wish to determine if a simple technique of inflating and deflating a blood pressure cuff immediately before the operation can reduce damage to the heart during surgery for two common conditions, to improve the outcomes of children's heart surgery.

For peer review only - http://bmjopen.bmj.com/site/about/guidelines.xhtml

What procedure is being tested in this study?

We are testing whether a simple procedure, the inflation and deflation of blood pressure cuffs on both legs immediately before heart surgery, can help to protect the heart from injury during surgery for different types of congenital heart disease. No drugs are being tested in this study, only the effects of cuff inflation.

How might inflating blood pressure cuffs on the legs help to protect the heart?

It has been shown that reducing the blood flow to the arms or legs for a short period can protect the heart, lung and kidneys from injury in adults and children undergoing different types of surgery. The temporary stoppage of blood to the limb activates a reflex known as remote ischaemic pre-conditioning (RIC). Researchers in several countries, including Canada & Australia, have shown that this may reduce the extent of heart damage in young children after surgery for congenital heart disease. We are performing this study to see whether there is a difference between children who have low oxygen levels in the blood (cyanosis) and those who do not.

Will my child undergo the blood pressure cuff treatment?

This study is a double-blind, randomised controlled trial. This means that if you agree for your child to take part, they will be allocated by a computer with a 50% chance of receiving RIC with the blood pressure cuffs and a 50% chance of not receiving RIC. All other aspects of the anaesthetic, surgery and post-operative care will be the same and *neither* you *nor* the surgical team will know whether your child has received RIC. At the end of the study, the code will be revealed to see which children were in which group. This is a standard technique for preventing those doctors and nurses involved in conducting a clinical trial from potentially influencing the results.

What will happen if I agree for my child to take part?

In addition to the standard operation and post-operative care, if you agree for your child to take part in the study, the following will occur:

- Your child's Paediatric Cardiologist and with your permission, your child's GP will be informed of their participation.
- Your child will be allocated to either the RIC group or the control group by chance.
- Once they are asleep under anaesthesia, if they are in the RIC group, a blood pressure cuff will be placed around each of their upper thighs and inflated to a

For peer review only - http://bmjopen.bmj.com/site/about/guidelines.xhtml

level approximately 50mmHg higher than their own blood pressure – this will not cause any pain. The cuffs will remain inflated for 5 minutes then deflated for 5 minutes and will be repeated two more times. If they are in the control group, the blood pressure cuffs will not be placed on their legs.

- Prior to surgery, all children have small plastic lines (tubes) inserted into their blood vessels to make measurements and take blood samples. Children in the study will have additional blood samples taken from these lines (no extra needles) over the first 24 hours after surgery to detect any injury to the heart.
- During surgery, in order to repair the defect, the heart is opened through a cut in the side of the right atrium. In children in the study, two small biopsies of the atrium will be taken from the edge of the cut by the operating surgeon. Once the heart defect is repaired, the cut will be closed in the normal way; taking the biopsies does not increase the risk of the operation in any way but will allow us to understand how RIC may improve protection of the heart.
- In children with TOF, bands of muscle inside the heart which blocks the flow of blood to the lungs are routinely removed during surgery; if your child is in the study, these bands of muscle will be kept for analysis rather than be thrown away.
- After surgery, your child will be discharged home and kept under regular follow-up in the clinic; you will not need to attend any additional clinic visits for the study.

What are the benefits?

There may not be any benefits for your child. Whilst some previous studies have shown that RIC helps to protect children's hearts from injury during surgery, we do not know whether it is beneficial to all children with all types of congenital heart disease - that is why we are conducting this study. We do not know whether being in the study will make your child's surgery safer but we are conducting it in order to understand how to improve the outcomes of children's heart surgery in the future.

Are there any risks?

Previous studies have shown that RIC is safe. There have been no complications reported related to the use of a blood pressure cuff for RIC in either children or adults undergoing *any* type of surgery. The operation itself carries a risk for your child, as will have been discussed with you by your Surgeon and Cardiologist, but being involved in this study does not increase that risk in any way.

For peer review only - http://bmjopen.bmj.com/site/about/guidelines.xhtml

How many children will be taking part in this study?

We aim to recruit up to 120 children undergoing surgery for congenital heart disease in Birmingham and Leeds to take part in this study over a 4 year period.

Does my child have to take part?

Taking part in the study is entirely voluntary – you decide. This parent information sheet gives you information about the study and we can answer any questions that you may have after reading it. Before your child's surgery, one of the research team will ask you whether you wish your child to participate in this study and if so, to sign a consent form. Your child will only be included in the study if you give your express permission. Indeed, you are free to withdraw your child at any time, without giving a reason – their surgery will proceed as planned, without any additional measurements and tests, and it will not affect the standard of care that your child receives.

What if something goes wrong?

The standard care of children undergoing heart surgery involves intensive monitoring and we do not expect the study itself to cause any problems. Complications of surgery can occur and these will be dealt with in the normal manner, regardless of the research study. Your child's safety during and after surgery is paramount. In the unlikely event that any harm should occur as a result of taking part in this study, we want you to be informed of your rights. There are no special compensation arrangements but you may have the right to claim damages in a court of law; this would require you to prove fault on the part of the NHS Trust, University or any manufacturer involved. The standard NHS complaints mechanisms are available to you; further information can be obtained from the Patient Advice & Liaison Service (PALS) at Birmingham Children's Hospital on 0121 333 8611.

What happens to my child's information and samples?

All information collected on children who participate in this study will be securely stored on Hospital and University computers. Paper copies of the data will be stored in a locked office at the Hospital. The information from the study will be analysed, presented at scientific meetings and published in medical journals to inform other doctors and health professionals of the research findings. All data will be coded and kept confidential, ensuring that your child's identity will not be revealed at any time.

For peer review only

For peer review only - http://bmjopen.bmj.com/site/about/guidelines.xhtml

All necessary measures will be taken to keep your child's data safe and to comply with the Data Protection Act. Following completion of the study, the data will be kept for 25 years then destroyed in accordance with national guidance. All of the blood and tissue samples collected during the study will stored in secure laboratories at the hospital, collaborating hospitals & University in accordance with Human Tissue Act. Once analysed, any remaining samples may be kept and used in future research studies which conform to all relevant legal, governance and ethical requirements.

What happens at the end of the study?

At the end of the study, your child's treatment and follow-up continues as would that of a child who had not been involved in the study.

Will I ever know if the trial worked and which treatment my child received?

Yes – but not until the whole study has finished and we have analysed the results. We will work with *Young at Heart*, a charity that offers help and support to families of babies and children diagnosed with heart defects, to produce a newsletter with the findings of the study to send to the parents of all children involved. At this stage, we will be able to tell you which group your child was in.

Who is organising and funding this research?

This study has been organised & developed by the teams at Birmingham Children's Hospital, Leeds Teaching Hospitals NHS Trust and University of Birmingham. It is funded by the **British Heart Foundation** & sponsored by University of Birmingham.

Who has reviewed this research study and leaflet?

The study has been reviewed by the British Heart Foundation and the Research & Development teams at Birmingham Children's Hospital, Leeds Teaching Hospitals NHS Trust and the University of Birmingham. It has been given a favourable opinion for conduct in the NHS by the West Midlands Solihull Research Ethics Committee. This Parent Information Sheet has been reviewed and revised by the parents of children who have had heart surgery, through the children's charity *Young at Heart*.

Questions? Contact Mr. Nigel Drury, Consultant in Paediatric Cardiac Surgery by email: xxxxx.xxxxx@nhs.net or via the BCH switchboard on xxxx xxx xxxx.

For peer review only - http://bmjopen.bmj.com/site/about/guidelines.xhtml

Flow chart for the Bilateral Remote Ischaemic Conditioning in Children trial

You will have been given this information sheet by your Consultant or a member of the research team in the clinic or been sent it in the post.

↓

Please read it carefully and consider whether you would like your child to take part. If you have any questions, please feel free to ask.

↓

We will contact you either at the pre-operative assessment clinic or when your child is admitted for their operation.

↓

If you are happy for your child to take part, you will be asked to sign a Consent form by a member of the research team.

↓

On the day of surgery, you may go along to theatre with your child as usual. After they have gone to sleep, the computer will allocate them to a group and they will receive *either* RIC *or* no RIC just before their operation. During surgery, the blood & tissue samples will be removed for analysis.

↓

After the operation, they will be transferred to the Paediatric Intensive Care Unit (PICU) as usual and you will be able to see them there.

↓

Whilst they are recovering on PICU, several additional blood tests will be performed using the lines already in place – no new needles required.

↓

When they are well enough, your child will be transferred to the ward and then discharged from hospital once they are ready to go home.

↓

After discharge, they will be seen regularly in the outpatient clinic but there will be no additional follow-up appointment related to the study.

↓

Once the trial has completed, we will send you a newsletter with the results.

Thank you for reading this information & considering your child's participation

For peer review only

PARENT/GUARDIAN INFORMATION SHEET

The Bilateral Remote Ischaemic Conditioning in Children trial

Principal Investigator: Ms Carin van Doorn, Consultant Paediatric Cardiac Surgeon

An invitation to participate in research: The Heart Surgery team at Leeds Children's Hospital would like to invite your child to take part in a research study. Before you decide, you need to understand why the research is being done and what it would involve for you and your child. Please read the following information carefully and take time to decide whether or not you would like your child to take part. If there is anything that is not clear or you would like more information, please ask.

Why is my child being invited to take part?

Your child has been referred to the Heart Surgery team for an operation to repair one of two common congenital heart conditions: Tetralogy of Fallot (TOF) or a Ventricular Septal Defect (VSD). We are performing a clinical trial in young children with these conditions and have approached you to see if you would like your child to take part.

What is the aim of the study?

Children like yours are born with congenital heart disease and often need operations to correct the abnormality that they were born with to improve their survival. The surgery is complex and usually involves a period of support on a heart-lung machine (cardiopulmonary bypass) whilst the defect is repaired. The surgery puts a strain on your child's heart and may potentially cause damage (called ischaemia-reperfusion injury) when the blood supply to the heart is interrupted and restored. In this study, we wish to determine if a simple technique of inflating and deflating a blood pressure cuff immediately before the operation can reduce damage to the heart during surgery for two common conditions, to improve the outcomes of children's heart surgery.

For peer review only

For peer review only - http://bmjopen.bmj.com/site/about/guidelines.xhtml

What procedure is being tested in this study?

We are testing whether a simple procedure, the inflation and deflation of blood pressure cuffs on both legs immediately before heart surgery, can help to protect the heart from injury during surgery for different types of congenital heart disease. No drugs are being tested in this study, only the effects of cuff inflation.

How might inflating blood pressure cuffs on the legs help to protect the heart?

It has been shown that reducing the blood flow to the arms or legs for a short period can protect the heart, lung and kidneys from injury in adults and children undergoing different types of surgery. The temporary stoppage of blood to the limb activates a reflex known as remote ischaemic pre-conditioning (RIC). Researchers in several countries, including Canada & Australia, have shown that this may reduce the extent of heart damage in young children after surgery for congenital heart disease. We are performing this study to see whether there is a difference between children who have low oxygen levels in the blood (cyanosis) and those who do not.

Will my child undergo the blood pressure cuff treatment?

This study is a double-blind, randomised controlled trial. This means that if you agree for your child to take part, they will be allocated by a computer with a 50% chance of receiving RIC with the blood pressure cuffs and a 50% chance of not receiving RIC. All other aspects of the anaesthetic, surgery and post-operative care will be the same and *neither* you *nor* the surgical team will know whether your child has received RIC. At the end of the study, the code will be revealed to see which children were in which group. This is a standard technique for preventing those doctors and nurses involved in conducting a clinical trial from potentially influencing the results.

What will happen if I agree for my child to take part?

In addition to the standard operation and post-operative care, if you agree for your child to take part in the study, the following will occur:

- Your child's Paediatric Cardiologist and with your permission, your child's GP will be informed of their participation.
- Your child will be allocated to either the RIC group or the control group by chance.
- Once they are asleep under anaesthesia, if they are in the RIC group, a blood pressure cuff will be placed around each of their upper thighs and inflated to a

For peer review only - http://bmjopen.bmj.com/site/about/guidelines.xhtml

level approximately 50mmHg higher than their own blood pressure – this will not cause any pain. The cuffs will remain inflated for 5 minutes then deflated for 5 minutes and will be repeated two more times. If they are in the control group, the blood pressure cuffs will not be placed on their legs.

- Prior to surgery, all children have small plastic lines (tubes) inserted into their blood vessels to make measurements and take blood samples. Children in the study will have additional blood samples taken from these lines (no extra needles) over the first 24 hours after surgery to detect any injury to the heart.
- After surgery, your child will be discharged home and kept under regular follow-up in the clinic; you will not need to attend any additional clinic visits for the study.

What are the benefits?

There may not be any benefits for your child. Whilst some previous studies have shown that RIC helps to protect children's hearts from injury during surgery, we do not know whether it is beneficial to all children with all types of congenital heart disease - that is why we are conducting this study. We do not know whether being in the study will make your child's surgery safer but we are conducting it in order to understand how to improve the outcomes of children's heart surgery in the future.

Are there any risks?

Previous studies have shown that RIC is safe. There have been no complications reported related to the use of a blood pressure cuff for RIC in either children or adults undergoing *any* type of surgery. The operation itself carries a risk for your child, as will have been discussed with you by your Surgeon and Cardiologist, but being involved in this study does not increase that risk in any way.

How many children will be taking part in this study?

We aim to recruit up to 120 children undergoing surgery for congenital heart disease in Birmingham and Leeds to take part in this study over a 4 year period.

Does my child have to take part?

Taking part in the study is entirely voluntary – you decide. This parent information sheet gives you information about the study and we can answer any questions that you may have after reading it. Before your child's surgery, one of the research team

For peer review only - http://bmjopen.bmj.com/site/about/guidelines.xhtml

will ask you whether you wish your child to participate in this study and if so, to sign a consent form. Your child will only be included in the study if you give your express permission. Indeed, you are free to withdraw your child at any time, without giving a reason – their surgery will proceed as planned, without any additional measurements and tests, and it will not affect the standard of care that your child receives.

What if something goes wrong?

The standard care of children undergoing heart surgery involves intensive monitoring and we do not expect the study itself to cause any problems. Complications of surgery can occur and these will be dealt with in the normal manner, regardless of the research study. Your child's safety during and after surgery is paramount. In the unlikely event that any harm should occur as a result of taking part in this study, we want you to be informed of your rights. There are no special compensation arrangements but you may have the right to claim damages in a court of law; this would require you to prove fault on the part of the NHS Trust, University or any manufacturer involved. The standard NHS complaints mechanisms are available to you; further information can be obtained from the Patient Advice & Liaison Service (PALS) at Leeds Teaching Hospitals on 0113 206 6261.

What happens to my child's information and samples?

All information collected on children who participate in this study will be securely stored on Hospital and University computers. Paper copies of the data will be stored in a locked office at the Hospital. The information from the study will be analysed, presented at scientific meetings and published in medical journals to inform other doctors and health professionals of the research findings. All data will be coded and kept confidential, ensuring that your child's identity will not be revealed at any time. All necessary measures will be taken to keep your child's data safe and to comply with the Data Protection Act. Following completion of the study, the data will be kept for 25 years then destroyed in accordance with national guidance. All of the blood and tissue samples collected during the study will stored in secure laboratories at the hospital, collaborating hospitals & University in accordance with Human Tissue Act. Once analysed, any remaining samples may be kept and used in future research studies which conform to all relevant legal, governance and ethical requirements.

For peer review only - http://bmjopen.bmj.com/site/about/guidelines.xhtml

What happens at the end of the study?

At the end of the study, your child's treatment and follow-up continues as would that of a child who had not been involved in the study.

Will I ever know if the trial worked and which treatment my child received?

Yes – but not until the whole study has finished and we have analysed the results. We will work with *Young at Heart*, a charity that offers help and support to families of babies and children diagnosed with heart defects, to produce a newsletter with the findings of the study to send to the parents of all children involved. At this stage, we will be able to tell you which group your child was in.

Who is organising and funding this research?

This study has been organised & developed by the teams at Birmingham Children's Hospital, Leeds Teaching Hospitals NHS Trust and University of Birmingham. It is funded by the **British Heart Foundation** & sponsored by University of Birmingham.

Who has reviewed this research study and leaflet?

The study has been reviewed by the British Heart Foundation and the Research & Development teams at Birmingham Children's Hospital, Leeds Teaching Hospitals NHS Trust and the University of Birmingham. It has been given a favourable opinion for conduct in the NHS by the West Midlands Solihull Research Ethics Committee. This Parent Information Sheet has been reviewed and revised by the parents of children who have had heart surgery, through the children's charity *Young at Heart*.

Questions? Contact Ms Carin van Doorn, Clinical Lead & Consultant Congenital Cardiac Surgeon at Leeds Teaching Hospitals by phone: xxxxx xxxxxx, or Collette Spencer, Research Nurse by email: xxxxxxx@nhs.net or phone: xxxx xxx xxxx.

For peer review only - http://bmjopen.bmj.com/site/about/guidelines.xhtml

Flow chart for the Bilateral Remote Ischaemic Conditioning in Children trial

You will have been given this information sheet by your Consultant or a member of the research team in the clinic or been sent it in the post.

↓

Please read it carefully and consider whether you would like your child to take part. If you have any questions, please feel free to ask.

↓

We will contact you either at the pre-operative assessment clinic or when your child is admitted for their operation.

↓

If you are happy for your child to take part, you will be asked to sign a Consent form by a member of the research team.

↓

On the day of surgery, you may go along to theatre with your child as usual. After they have gone to sleep, the computer will allocate them to a group and they will receive *either* RIC *or* no RIC just before their operation. During surgery, a blood sample will be obtained for analysis.

↓

After the operation, they will be transferred to the Paediatric Intensive Care Unit (PICU) as usual and you will be able to see them there.

↓

Whilst they are recovering on PICU, several additional blood tests will be performed using the lines already in place – no new needles required.

↓

When they are well enough, your child will be transferred to the ward and then discharged from hospital once they are ready to go home.

↓

After discharge, they will be seen regularly in the outpatient clinic but there will be no additional follow-up appointment related to the study.

↓

Once the trial has completed, we will send you a newsletter with the results.

Thank you for reading this information & considering your child's participation

For peer review only - http://bmjopen.bmj.com/site/about/guidelines.xhtml

1 ☐ ☐ ☐ BRICC trial – ISRCTN12923441 IRAS: 200876 Version 1.9a: 22/11/2019

PARENT/GUARDIAN CONSENT FORM

The Bilateral Remote Ischaemic Conditioning in Children trial

Chief Investigator: Mr Nigel Drury, Consultant in Paediatric Cardiac Surgery, BCH

Please initial boxes

I confirm that I have read and understand the parent/guardian information sheet (version 1.9a, dated 22/11/2019) for the above study. I have had the opportunity to consider the study information, ask questions and have had these answered satisfactorily.	☐
I understand that my child's participation is voluntary and that I am free to withdraw at any time, without giving a reason and without my child's care or legal rights being affected.	☐
I understand that relevant sections of any of my child's medical notes and data collected during the study may be looked at by responsible individuals from the NHS Trusts, the University of Birmingham or the regulatory authorities, where it is relevant to my taking part in this research. I give permission for these individuals to have access to my child's records.	☐
I consent to the storage, including electronic, of personal information for the purposes of this study. I understand that any information that could identify me or my child will be kept strictly confidential and that no personal information will be included in the study report or other publication.	☐
I understand that blood and tissue samples will be kept for the purposes of research and I give permission for these samples to be taken and stored.	☐
I understand that any remaining samples may be stored beyond the end of this trial and used in future research which conforms to all relevant legal, governance and ethical requirements.	☐
I agree to my child's GP and/or other doctors involved in their care, being informed of my child's participation in the study.	☐

Name of Child: Date of birth:

Name of Parent/Guardian	Signature of Parent/Guardian	Date signed
Name of Investigator	Signature of Investigator	Date signed

Once completed: 1 for parent(s), 1 for medical notes and 1 (original) for trial site file

For peer review only

1 ☐ ☐ ☐ BRICC trial – ISRCTN12923441 IRAS: 200876 Version 1.9b: 22/11/2019

British Heart Foundation | UNIVERSITY OF BIRMINGHAM | BCTU Birmingham Clinical Trials Unit | The Leeds Teaching Hospitals NHS Trust | Leeds Children's Hospital

PARENT/GUARDIAN CONSENT FORM

The Bilateral Remote Ischaemic Conditioning in Children trial

Principal Investigator: Ms Carin van Doorn, Consultant Paediatric Cardiac Surgeon

Please initial boxes

I confirm that I have read and understand the parent/guardian information sheet (version 1.9b, dated 22/11/2019) for the above study. I have had the opportunity to consider the study information, ask questions and have had these answered satisfactorily. ☐

I understand that my child's participation is voluntary and that I am free to withdraw at any time, without giving a reason and without my child's care or legal rights being affected. ☐

I understand that relevant sections of any of my child's medical notes and data collected during the study may be looked at by responsible individuals from the NHS Trusts, the University of Birmingham or the regulatory authorities, where it is relevant to my taking part in this research. I give permission for these individuals to have access to my child's records. ☐

I consent to the storage, including electronic, of personal information for the purposes of this study. I understand that any information that could identify me or my child will be kept strictly confidential and that no personal information will be included in the study report or other publication. ☐

I understand that blood samples will be kept for the purposes of research and I give permission for these samples to be taken and stored. ☐

I understand that any remaining samples may be stored beyond the end of this trial and used in future research which conforms to all relevant legal, governance and ethical requirements. ☐

I agree to my child's GP and/or other doctors involved in their care, being informed of my child's participation in the study. ☐

Name of Child: Date of birth:

Name of Parent/Guardian	Signature of Parent/Guardian	Date signed
Name of Investigator	Signature of Investigator	Date signed

Once completed: 1 for parent(s), 1 for medical notes and 1 (original) for trial site file

For peer review only

Data Monitoring Committee (DMC) Charter

For peer review only

1. INTRODUCTION

Title: The Bilateral Remote Ischaemic Conditioning in Children (BRICC) trial: a two-centre, double-blind, randomised controlled trial of remote ischaemic preconditioning in normoxic and hypoxic children undergoing cardiac surgery – ISRCTN12923441.

Chief Investigator: Mr Nigel Drury, Consultant in Paediatric Cardiac Surgery, BCH

TMC Chair: Mr Timothy Jones, Consultant Paediatric Cardiac Surgeon, BCH

IRAS: 200876, protocol: v1.6, 05/02/18, approved by REC: 28/02/18, HRA: 28/02/18.

Sponsor: University of Birmingham (RG_14-025), in partnership with Birmingham Women's and Children's NHS Foundation Trust (1845) and Leeds Teaching Hospitals NHS Trust (PA17/67348).

Funder: British Heart Foundation (FS/15/49/31612)

Objectives: The BRICC trial is a two-centre, double-blind, randomised controlled trial to assess whether adequately delivered remote ischaemic preconditioning is cardioprotective in young children undergoing surgical repair of two common congenital heart defects, through clinical and biochemical endpoints. It will also evaluate the effects of preconditioning in the presence or absence of chronic preoperative hypoxia by evaluating the myocardial metabolic phenotype.

Scope: The purpose of this document is to describe the roles and responsibilities of the independent DMC for the BRICC trial, including the terms of reference, the frequency and format of meetings, methods of providing information to and from the DMC, statistical issues and relationship with the Trial Management Committee.

2. ROLES & RESPONSIBILITIES

Aims: To safeguard the interests of trial participants & their families, assess the safety and efficacy of the interventions during the trial and monitor the overall conduct of the clinical trial.

Terms of reference: The DMC should receive and review the progress and accruing data from the trial and make recommendations on the conduct of the trial to the Trial Management Committee. Meetings will be scheduled approximately every 8 months and key outputs will be made available for review; changes to this schedule may be made by the DMC as required. Review(s) will include updated figures on recruitment, data quality, efficacy outcomes and safety data. Specifically, the DMC will:

- Assess data quality, including completeness
- Monitor recruitment and losses to follow-up
- Monitor compliance with the protocol by participants and investigators
- Monitor evidence for treatment differences in the main efficacy outcomes
- Monitor evidence of treatment harm (limb complications, SAEs)
- Decide whether to recommend that the trial continues or whether recruitment should be terminated for some or all of the treatment groups
- Suggest additional data analyses
- Advise on protocol modifications suggested by the Investigators
- Assess the impact and relevance of external evidence
- Monitor compliance with previous DMC recommendations

Furthermore, members of the DMC will not disclose interim results or use them for financial gain, nor share blinded data with anyone outside of the DMC, including the Chief Investigator. All DMC and TMC members will sign a Conflict of Interest form.

3. BEFORE OR EARLY IN THE TRIAL

Members of the DMC were invited to participate in the committee in the spring of 2016 and therefore were not involved in development of the trial protocol. The DMC were asked to meet before or early in the trial (within 3 months of commencing recruitment) to review this Charter, revise as required and approve the working version; this initial meeting took place on 18th November 2016.

For peer review only - http://bmjopen.bmj.com/site/about/guidelines.xhtml

4. COMPOSITION

The members of the independent DMC for the BRICC trial are:

- Prof Gavin Murphy, BHF Chair of Cardiac Surgery, University of Leicester (Chair)
- Dr Katherine Brown, Consultant in Cardiac Intensive Care, GOSH, London
- Dr Peter Nightingale, Statistician, Queen Elizabeth Hospital Birmingham

At their initial meeting, the members of the DMC nominated Prof Murphy as Chair with responsibility for facilitating discussion and communicating review outcomes.

5. RELATIONSHIPS

In addition to the independent DMC, the conduct of the trial is overseen by members of the Trial Management Committee, who have developed and approved the trial protocol and have executive responsibility for the conduct of the trial; there is no Trial Steering Committee. The DMC has an advisory role and will make recommendations regarding efficacy and the safety of participants to the Trial Management Committee.

6. ORGANISATION

The DMC discussed the first version of this Charter and made recommendations based on best practice which have been implemented. During the trial, they will meet every 8 months to review efficacy and safety data. Analysis of hs-troponin-T for the primary outcome will be performed prior to each meeting and the unblinded data made available. Meetings may be held via teleconference, without the need to meet face-to-face unless deemed necessary, and will employ the following format:

1. *Open session*: Introduction, presentation of progress report
2. *Closed session*: Discussion of closed parts of the report
3. *Open session*: Discussion of any matters arising from previous sessions
4. *Closed session*: Further discussion, if required.

Closed sessions will be attended only by members of the DMC and others whom are specifically invited as discussion will include unblinded efficacy and safety data by treatment group. In open sessions, they will be joined by the Chief Investigator, Trial Statistician and other interested parties which may include Research Nurse(s) and representatives of the R&D Office, sponsor, funder or regulator, as required.

For peer review only - http://bmjopen.bmj.com/site/about/guidelines.xhtml

7. DOCUMENTATION

The Chief Investigator and Trial Statistician will produce a progress report for each DMC review, which should be received by members at least 2 weeks beforehand, documenting the key outputs of the trial including graphs showing expected and observed recruitment, CONSORT diagrams, descriptions of the trial cohort, protocol compliance, procedural and outcome measures, and adverse events reported by group. Specifically it will include:

- *Primary efficacy outcome*: area under the time-concentration curve for hs-troponin-T release in the first 24 hours after aortic cross-clamp release, calculated by the trapezoid rule from samples taken at baseline, 3, 6, 12 and 24 hours.
- *Primary safety outcome*: frequency of expected Serious Adverse Events (SAEs) reported to the Sponsor: death; requirement for extracorporeal life support (ECLS); evidence of a major neurological event; or the need for further surgery in the early post-operative period, such as for bleeding or a residual VSD.
- *Secondary efficacy outcomes*: peak hs-troponin-T in the first 12 hours; vasoactive inotrope score in the first 12 hours; arterial lactate and central venous oxygen saturations in first 12 hours; and lengths of stay in the paediatric intensive care unit and the hospital. Cardiac index in the first 12 hours is an exploratory outcome.

DMC members should store their papers and reports safely after each meeting so that they can cross-check with later reports. After the trial is finally reported, all interim reports should be destroyed.

8. DECISION MAKING

The DMC should consider the following possible recommendations following interim analysis of the conduct of the trial and the safety and efficacy data:

1. No action needed, trial continues as planned.
2. Early stopping due to clear treatment benefit or harm, futility or external evidence.
3. Stopping recruitment within a subgroup.
4. Extending recruitment (based on actual control arm response rates being different to predicted rather than on emerging differences) or extending follow-up.
5. Sanctioning and/or proposing protocol changes.

For peer review only - http://bmjopen.bmj.com/site/about/guidelines.xhtml

The DMC agreed that no firm statistical criteria (often called *stopping rules*) would be defined, rather DMC recommendations would be based on the ability of the trial to test its primary hypothesis and the overall interpretation of the safety data and secondary outcomes that include important safety endpoints. The DMC will consider the balance of primary risks and benefits, the internal consistency of results, the consistence with and nature of external evidence, and the likelihood that results would affect clinical practice. Decisions should be achieved by consensus and be unanimous when possible, using both informal and formal decision-making strategies, as required.

If the DMC recommends stopping recruitment, an urgent face-to-face meeting should be arranged between the DMC Chair, Chief Investigator, key members of the Trial Management Committee and the Sponsor to reach a decision on continuing the trial.

9. REPORTING

The DMC will report its recommendations in writing to the Trial Management Committee and Sponsor. A formal record should also be made of both closed and open sessions, documenting the major points of discussion, any decisions and actions and their reasons, and any additional information needed for future meetings; however, names do not need to be attributed to all comments.

10. AFTER THE TRIAL

Responsibility for reporting the findings of the trial lies with the Chief Investigator and Trial Management Committee, although the DMC should encourage timely reporting. The DMC Chair should approve the accuracy of trial manuscripts submitted for publication in which all members of the DMC will be acknowledged.

REFERENCES

- DAMOCLES Study Group. A proposed charter for clinical trial data monitoring committees: helping them to do their job well. *Lancet* 2005; 365: 711-22.
- National Research Ethics Service. Data Monitoring Committees in Clinical Trials. Guidance for Research Ethics Committees. NPSA, May 2010

For peer review only - http://bmjopen.bmj.com/site/about/guidelines.xhtml

STANDARD PROTOCOL ITEMS: RECOMMENDATIONS FOR INTERVENTIONAL TRIALS

SPIRIT 2013 Checklist: Recommended items to address in a clinical trial protocol and related documents*

Section/item	Item No	Description	Addressed on page number
**Administrative information**			
Title	1	Descriptive title identifying the study design, population, interventions, and, if applicable, trial acronym	____1______
Trial registration	2a	Trial identifier and registry name. If not yet registered, name of intended registry	____2______
	2b	All items from the World Health Organization Trial Registration Data Set	__ISRCTN__
Protocol version	3	Date and version identifier	____22_____
Funding	4	Sources and types of financial, material, and other support	____21_____
Roles and responsibilities	5a	Names, affiliations, and roles of protocol contributors	____1,21___
	5b	Name and contact information for the trial sponsor	____18_____
	5c	Role of study sponsor and funders, if any, in study design; collection, management, analysis, and interpretation of data; writing of the report; and the decision to submit the report for publication, including whether they will have ultimate authority over any of these activities	____21-22___
	5d	Composition, roles, and responsibilities of the coordinating centre, steering committee, endpoint adjudication committee, data management team, and other individuals or groups overseeing the trial, if applicable (see Item 21a for data monitoring committee)	____15_____

For peer review only

Introduction

Background and rationale	6a	Description of research question and justification for undertaking the trial, including summary of relevant studies (published and unpublished) examining benefits and harms for each intervention	____4-6____
	6b	Explanation for choice of comparators	____6______
Objectives	7	Specific objectives or hypotheses	____6______
Trial design	8	Description of trial design including type of trial (eg, parallel group, crossover, factorial, single group), allocation ratio, and framework (eg, superiority, equivalence, noninferiority, exploratory)	____7-8____

Methods: Participants, interventions, and outcomes

Study setting	9	Description of study settings (eg, community clinic, academic hospital) and list of countries where data will be collected. Reference to where list of study sites can be obtained	____7______
Eligibility criteria	10	Inclusion and exclusion criteria for participants. If applicable, eligibility criteria for study centres and individuals who will perform the interventions (eg, surgeons, psychotherapists)	____7______
Interventions	11a	Interventions for each group with sufficient detail to allow replication, including how and when they will be administered	____9-10____
	11b	Criteria for discontinuing or modifying allocated interventions for a given trial participant (eg, drug dose change in response to harms, participant request, or improving/worsening disease)	____9______
	11c	Strategies to improve adherence to intervention protocols, and any procedures for monitoring adherence (eg, drug tablet return, laboratory tests)	____10_____
	11d	Relevant concomitant care and interventions that are permitted or prohibited during the trial	____10-11___
Outcomes	12	Primary, secondary, and other outcomes, including the specific measurement variable (eg, systolic blood pressure), analysis metric (eg, change from baseline, final value, time to event), method of aggregation (eg, median, proportion), and time point for each outcome. Explanation of the clinical relevance of chosen efficacy and harm outcomes is strongly recommended	____12-13___
Participant timeline	13	Time schedule of enrolment, interventions (including any run-ins and washouts), assessments, and visits for participants. A schematic diagram is highly recommended (see Figure)	___Figure 1___

For peer review only - http://bmjopen.bmj.com/site/about/guidelines.xhtml

Sample size	14	Estimated number of participants needed to achieve study objectives and how it was determined, including clinical and statistical assumptions supporting any sample size calculations	____13____
Recruitment	15	Strategies for achieving adequate participant enrolment to reach target sample size	____13-15__
**Methods: Assignment of interventions (for controlled trials)**			
Allocation:			
Sequence generation	16a	Method of generating the allocation sequence (eg, computer-generated random numbers), and list of any factors for stratification. To reduce predictability of a random sequence, details of any planned restriction (eg, blocking) should be provided in a separate document that is unavailable to those who enrol participants or assign interventions	____8-9____
Allocation concealment mechanism	16b	Mechanism of implementing the allocation sequence (eg, central telephone; sequentially numbered, opaque, sealed envelopes), describing any steps to conceal the sequence until interventions are assigned	____8-9____
Implementation	16c	Who will generate the allocation sequence, who will enrol participants, and who will assign participants to interventions	____8-9____
Blinding (masking)	17a	Who will be blinded after assignment to interventions (eg, trial participants, care providers, outcome assessors, data analysts), and how	____9______
	17b	If blinded, circumstances under which unblinding is permissible, and procedure for revealing a participant's allocated intervention during the trial	____N/A____
**Methods: Data collection, management, and analysis**			
Data collection methods	18a	Plans for assessment and collection of outcome, baseline, and other trial data, including any related processes to promote data quality (eg, duplicate measurements, training of assessors) and a description of study instruments (eg, questionnaires, laboratory tests) along with their reliability and validity, if known. Reference to where data collection forms can be found, if not in the protocol	____11-13___
	18b	Plans to promote participant retention and complete follow-up, including list of any outcome data to be collected for participants who discontinue or deviate from intervention protocols	____N/A____

Data management	19	Plans for data entry, coding, security, and storage, including any related processes to promote data quality (eg, double data entry; range checks for data values). Reference to where details of data management procedures can be found, if not in the protocol	____16_____
Statistical methods	20a	Statistical methods for analysing primary and secondary outcomes. Reference to where other details of the statistical analysis plan can be found, if not in the protocol	____14_____
	20b	Methods for any additional analyses (eg, subgroup and adjusted analyses)	____14_____
	20c	Definition of analysis population relating to protocol non-adherence (eg, as randomised analysis), and any statistical methods to handle missing data (eg, multiple imputation)	____14_____
**Methods: Monitoring**			
Data monitoring	21a	Composition of data monitoring committee (DMC); summary of its role and reporting structure; statement of whether it is independent from the sponsor and competing interests; and reference to where further details about its charter can be found, if not in the protocol. Alternatively, an explanation of why a DMC is not needed	__15, App E__
	21b	Description of any interim analyses and stopping guidelines, including who will have access to these interim results and make the final decision to terminate the trial	____App E__
Harms	22	Plans for collecting, assessing, reporting, and managing solicited and spontaneously reported adverse events and other unintended effects of trial interventions or trial conduct	____14-15__
Auditing	23	Frequency and procedures for auditing trial conduct, if any, and whether the process will be independent from investigators and the sponsor	____16_____
**Ethics and dissemination**			
Research ethics approval	24	Plans for seeking research ethics committee/institutional review board (REC/IRB) approval	____18_____
Protocol amendments	25	Plans for communicating important protocol modifications (eg, changes to eligibility criteria, outcomes, analyses) to relevant parties (eg, investigators, REC/IRBs, trial participants, trial registries, journals, regulators)	____18_____

Consent or assent	26a	Who will obtain informed consent or assent from potential trial participants or authorised surrogates, and how (see Item 32)	____8______
	26b	Additional consent provisions for collection and use of participant data and biological specimens in ancillary studies, if applicable	____16_____
Confidentiality	27	How personal information about potential and enrolled participants will be collected, shared, and maintained in order to protect confidentiality before, during, and after the trial	____15-16___
Declaration of interests	28	Financial and other competing interests for principal investigators for the overall trial and each study site	____22_____
Access to data	29	Statement of who will have access to the final trial dataset, and disclosure of contractual agreements that limit such access for investigators	____14_____
Ancillary and post-trial care	30	Provisions, if any, for ancillary and post-trial care, and for compensation to those who suffer harm from trial participation	___App A/B__
Dissemination policy	31a	Plans for investigators and sponsor to communicate trial results to participants, healthcare professionals, the public, and other relevant groups (eg, via publication, reporting in results databases, or other data sharing arrangements), including any publication restrictions	____19_____
	31b	Authorship eligibility guidelines and any intended use of professional writers	____19_____
	31c	Plans, if any, for granting public access to the full protocol, participant-level dataset, and statistical code	____19_____
**Appendices**			
Informed consent materials	32	Model consent form and other related documentation given to participants and authorised surrogates	_Appendices_
Biological specimens	33	Plans for collection, laboratory evaluation, and storage of biological specimens for genetic or molecular analysis in the current trial and for future use in ancillary studies, if applicable	__12,15-16__

*It is strongly recommended that this checklist be read in conjunction with the SPIRIT 2013 Explanation & Elaboration for important clarification on the items. Amendments to the protocol should be tracked and dated. The SPIRIT checklist is copyrighted by the SPIRIT Group under the Creative Commons "Attribution-NonCommercial-NoDerivs 3.0 Unported" license.

For peer review only - http://bmjopen.bmj.com/site/about/guidelines.xhtml
